# Trends analysis of cancer incidence, mortality, and survival for the elderly in the United States, 1975–2020

**DOI:** 10.1002/cam4.70062

**Published:** 2024-07-31

**Authors:** Jia Xu, Jingyuan Liao, Qiong Yan, Jiang Jiao, Nan Hu, Wei Zhang, Lei Shi, Mingming Deng, Shu Huang, Xiaowei Tang

**Affiliations:** ^1^ Department of Gastroenterology The Affiliated Hospital of Southwest Medical University Luzhou China; ^2^ Nuclear Medicine and Molecular Imaging Key Laboratory of Sichuan Province Luzhou China; ^3^ Department of Gastroenterology Lianshui County People' Hospital Huaian China; ^4^ Department of Gastroenterology Lianshui People' Hospital of Kangda College Affiliated to Nanjing Medical University Huaian China

**Keywords:** Cancer, Elderly, Epidemiology, SEER

## Abstract

**Background:**

Cancer burden from the elderly has been rising largely due to the aging population. However, research on the long‐term epidemiological trends in cancer of the elderly is lacking.

**Methods:**

Registry data of this population‐based cross‐sectional study were from the Surveillance, Epidemiology, and End Results (SEER) database. The study population aged 65 years or more, from geographically distinct regions. Joinpoint regression and JP Surv method were used to analyze cancer trends and survival.

**Results:**

Mortality rate during 1975–2020 decreased from 995.20 to 824.99 per 100,000 elderly persons, with an average annual decrease of 0.421% (95% CI, 0.378–0.464). While overall incidence increased with no significance. Prostate (29%) and breast (26%) cancer were the most common malignancies, respectively, in elderly males and females, and the mortality for both of the two (prostate 15%, breast 14%) ranked just behind lung and bronchus cancer, which had the highest mortality rates in males (29%) and females (23%). Many cancers showed adverse trends in the latest follow‐up periods (the last period calculated by the Joinpoint method). For intrahepatic cholangiocarcinoma, incidence (male Annual Percentage Change [APC] = 7.4*; female APC = 6.7*) and mortality (male APC = 3.0*; female APC = 3.3*) increased relatively fast, and its survival was also terrible (3‐year survival only 10%). Other cancers with recent increasing mortality included cancer of anus, anal canal and anorectum, retroperitoneum, pleura, peritoneum, etc. Most cancers had favorable trends of survival during the nearest follow‐up period.

**Conclusion:**

Against the background of overall improvement, many cancers showed adverse trends. Further research for the underlying mechanisms and targeted implements towards adverse trends is also urgent.

## 1 INTRODUCTION

Cancer had been the second cause of death just behind heart diseases in the United States (US) during 2015–2020.^1^, ^2^ It is estimated that there will be 1,958,310 new cancer cases and 609,820 cancer deaths in the US in 2023, and over half of the newly diagnosis and over 70% deaths are elderly patients (defined as persons aged 65 years or more in this study).^3^ With the extension of human life expectancy and aging population, the cancer burden would become heavier in this age group, raising growing health concerns and social panic.^4^, ^5^ In addition, elderly patients were more likely to develop cancer complications, which added to the risk of death.^6^ Therefore, it is necessary to monitor cancer epidemiology in the elderly and make accurate strategies to reduce future burdens.

As is well known, age is a strongly positive‐related hazard factor for most cancers' occurrences.^7^, ^8^ And there is evidence that elderly patients were often more advanced at diagnosis and also less likely to receive surgical treatment compared to the younger ones.^9^ Distribution of cancer was also distinct that leading cancers in adolescents and young adults (aged 15–39 years) were thyroid cancer, testicular germ cell tumors, lymphoma, and leukemia, while in the oldest old (aged 85 years or more) were cancer of lung and bronchus, colon and rectum, breast, and prostate.^10^ For the overall population, previous studies elucidated that cancer incidence and mortality declined from 2013 to 2016 in the US. Specifically, incidence of liver and intrahepatic bile duct cancer increased the fastest in both genders during 2012–2016, while mortality of oral cavity and pharynx cancer in male and corpus and uterus cancer in female increased most rapidly during 2013–2017.^11^ And steepest death decline was found in cancer of lung and melanoma (decreased more than 4% per year) during 2015–2019 in total US population.^12^

To explore cancer epidemiology in the elderly with the updated statistics, we analyzed cancer incidence, mortality, and survival during 1975–2020 in the US. Overall and site‐specific cancer data by age, sex, and race/ethnicity were included.

## 2 METHODS

### 2.1 Data acquisition

The elderly in our study were defined as those aged 65 years or more and divided by 5‐year age group (65–69, 70–74, 75–79, 80–84, and 85+ years). Cancer cases diagnosed between January 1, 1975, and December 31, 2020, were extracted from the National Cancer Institute's SEER 8 and SEER 17 (respectively covers 8.3% and 26.5% of the US population) database. Although SEER 8 covers the least population, it has the longest time span from 1975 to 2020, which provides us with data during 1975–1999. And SEER 17 was used for 2000–2020 data.^13^ Mortality data was extracted from SEER*Stat software 8.4.2 and provided by NCHS, which was based on information from all death certificates filed in the 50 states and the District of Columbia.

SEER*Stat software provides data on incidence, mortality and survival by age at diagnosis/death, year of diagnosis/death, sex, race, stage, site, etc. Stage of blood cancers were excluded for different staging system from solid cancer and SEER data blankage.^14^ Incidence cases diagnosed from autopsy or death certificate only were excluded. Both incidence and mortality rates were age adjusted to the 2000 US standard population. Relative survival data excluded the last diagnosis year (2020 in our study), which may miss some of the deaths and survival may be overestimated. Relative survival is a net cancer survival using cohort method in the absence of other causes of death, which is defined as the ratio of the proportion of observed survivors in a cohort of cancer patients to the proportion of expected survivors in a comparable set of cancer‐free individuals.^15^

Incidence cases were screened using site recode ICD‐O‐3 (International Classification of Disease for Oncology, Third Edition)/WHO (World Health Organization) 2008, which was based on ICD‐O‐3 and updated for hematopoietic codes based on the WHO Classification of Tumors of Hematopoietic and Lymphoid Tissues (2008, Table S1). Mortality data was classified by ICD 8–10 (The International Statistical Classification of Diseases and Related Health Problems 8th/10th Revision). A total of 46 types of cancer were included in this study (Table S2). All cancers' frequencies, age‐adjusted incidence/mortality rates, as well as relative survival of the elderly, were extracted, but only 12 cancers with the highest incidence or mortality rates, respectively, in males and females were displayed in the text (data for other cancers were available in Appendix S1).

### 2.2 Statistical analysis

Temporal trends in incidence and mortality were analyzed by the Joinpoint software (version 5.0.2), which were presented as annual percentage change (APC) and average annual percentage change (AAPC). *p*‐Value is not available for the Joinpoint default method, Empirical Quantile. JPSurve method was used to analyze 1‐, 3‐, and 5‐year relative survival rates by calendar year, and trends in survival were represented as average absolute change in survival (AAC_S).^16^ For example, 1‐year relative survival AAC_S of oral cavity and pharynx cancer was 0.09% from 1975 to 2005, which means 1‐year relative survival rate of 1976 = 75.9% (1‐year relative survival rate of 1975) + 0.09%. Both Joinpoint and JPSurv used a maximum joint number of two. Other Figures were produced by GraphPad Prism 9 (version 9.5.1).

## 3 RESULTS

### 3.1 Elderly cancer demographics

Table S3–S6 presented the demographic characteristics of cancer incidence and mortality for the overarching picture of 1975–2020 and the recent picture of 2000–2020. It was shown that male (54.0%) and white (85.4%) patients accounted for large parts of cases diagnosed during 1975–2020. A similar distribution could be seen in the cohort diagnosed from 2000 to 2020. Males had a larger proportion of incidence and death in each age subgroups than females except for those ≥85 years old (1975–2020 incidence: 45.0% male vs. 55.0% female; 1975–2020 death: 45.2% male vs. 54.8% female).

**FIGURE 1:**
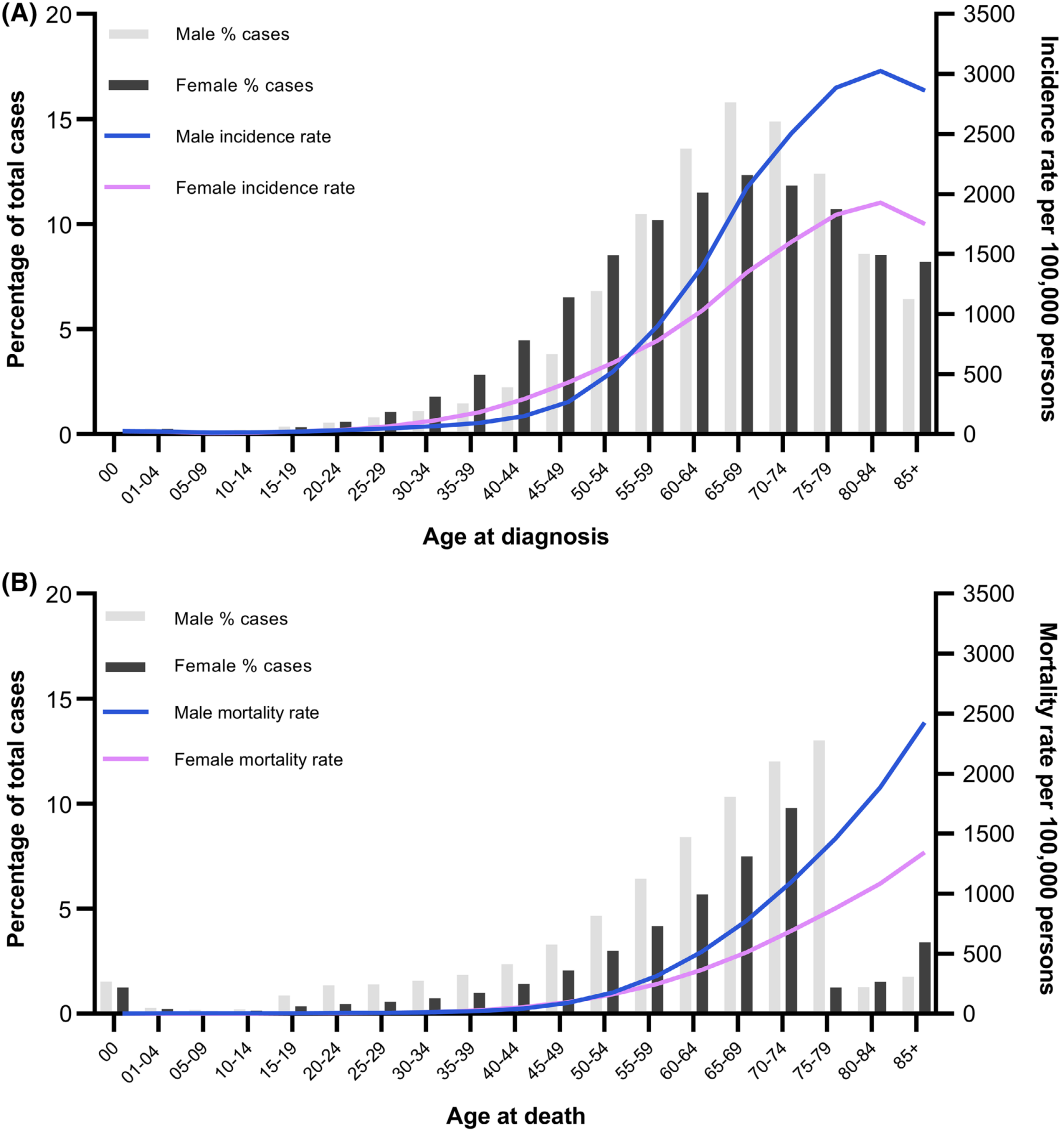
Overall cancer case distribution and average annual incidence (A) and death (B) rate per 100,000 persons by age and sex in the elderly population, United States, 1975–2020.

The proportion of cancer patients incidence peaked at 65–69 year age group (25.7% in 65–69 years; 24.4% in 70–74 years; 21.1% in 75–79 years; 15.6% in 80–84 years; 13.3% in 85+ years; Figure 1). The proportion of cancer patients' deaths increased with increasing age and met a plunge in females aged 75 years and males aged 80 years. Age‐adjusted incidence rates peaked at 80–84 years and then decreased as patients aged, while age‐adjusted mortality rates always kept a positive correlation with age.

### 3.2 Elderly cancer distribution

Prostate cancer (29%) and breast cancer (26%) were the most common cancers in males and females, respectively, in while cancer of lung and bronchus was the most common cancer‐related cause of death in both men (29%) and women (23%, Figure S1). Table 1 and Table S7 showed 12 cancers' distribution by age and sex with the highest incidence or mortality rate. As people get older, although the proportion of overall incidence decreased, incidence of female cancers of colon and rectum, pancreas, urinary bladder, stomach, and leukemia increased. Prostate cancer (*n* = 3,411,06) surpassed lung and bronchus (*n* = 269,768) cancer to be the leading cancerous cause of death in males aged 85+ years. Cancer of colon and rectum (*n* = 284,128) surpassed cancer of lung and bronchus (*n* = 255,017) and breast (*n* = 236,849) in mortality for females aged 85+ years. Cancers of digestive system (20.7%), respiratory system (16.1%), and male genital system (15.8%, Table S8) accounted for the majority of cancer incidence. Respiratory cancers (31.5%) ranked first in cancer mortality (Table S9).

**TABLE 1:** Cancer incidence distribution by sex and age, United States, 1975–2020.^a^

Cancer subtype^b^	Age group, No. (%)					
	65–69 years	70–74 years	75–79 years	80–84 years	85+ years	Total
Male						
All sites	808,905 (27.2)	761,126 (25.6)	633,845 (21.3)	438,850 (21.3)	328,698 (11.0)	2,971,424 (100.0)
Prostate	284,947 (33.2)	244,128 (28.4)	174,236 (20.3)	96,034 (20.3)	60,095 (7.0)	859,440 (100.0)
Lung and bronchus	116,166 (25.5)	119,728 (26.2)	104,798 (23.0)	70,770 (23.0)	44,915 (9.0)	456,377 (100.0)
Colon and rectum	72,770 (24.2)	72,138 (24.0)	64,873 (21.6)	50,262 (21.6)	40,942 (13.6)	300.985 (100.0)
Urinary bladder	47,688 (20.2)	53,531 (22.7)	53,298 (22.6)	43,478 (22.6)	37,884 (16.1)	235,879 (100.0)
Melanoma of the skin	33,177 (24.9)	32,001 (24.0)	27,879 (20.9)	21,582 (20.9)	18,749 (14.1)	133,388 (100.0)
Non‐Hodgkin lymphoma	27,841 (24.0)	27,554 (23.7)	25,714 (22.2)	19,503 (22.2)	15,420 (13.3)	116,032 (100.0)
Kidney and Renal pelvis	29,202 (30.7)	25,411 (26.7)	19,706 (20.7)	12,570 (20.7)	8226 (8.0)	95,115 (100.0)
Leukemia	19,987 (22.3)	20,927 (23.4)	19,795 (22.1)	15,640 (22.1)	13,227 (14.8)	89,576 (100.0)
Pancreas	20,222 (24.4)	20,244 (24.4)	17,891 (21.6)	13,724 (21.6)	10,760 (13.0)	82,841 (100.0)
Oral cavity and pharynx	25,092 (33.0)	19,889 (26.2)	14,214 (18.7)	9547 (18.7)	7228 (9.0)	75,970 (100.0)
Stomach	14,533 (22.9)	15,072 (23.8)	13,855 (21.9)	10,855 (21.9)	9013 (14.2)	63,328 (100.0)
Liver^c^	16,034 (34.3)	11,929 (25.5)	9106 (19.5)	5870 (19.5)	3771 (8.0)	46,710 (100.0)
Female						
All sites	603,971 (23.9)	579,604 (22.9)	524,477 (20.7)	417,510 (20.7)	402,356 (15.9)	2,527,918 (100.0)
Breast	185,007 (28.6)	160,606 (24.8)	129,852 (20.1)	92,002 (20.1)	78,859 (12.2)	646,326 (100.0)
Lung and bronchus	88,844 (23.3)	95,816 (25.1)	87,323 (22.9)	62,650 (22.9)	46,655 (12.2)	381,288 (100.0)
Colon and rectum	56,924 (18.0)	61,707 (19.5)	65,739 (20.8)	61,809 (20.8)	69,737 (22.1)	315,916 (100.0)
Corpus uteri	45,417 (34.1)	35,092 (26.4)	24,860 (18.7)	16,014 (18.7)	11,711 (8.0)	133,094 (100.0)
Non‐Hodgkin lymphoma	23,074 (20.9)	24,083 (21.8)	23,990 (21.7)	20,149 (21.7)	19,216 (17.4)	110,512 (100.0)
Pancreas	16,949 (18.4)	18,751 (20.4)	19,504 (21.2)	17,413 (21.2)	19,304 (21.0)	91,921 (100.0)
Urinary bladder	13,670 (17.7)	15,453 (20.0)	16,293 (21.1)	14,987 (21.1)	16,786 (21.7)	77,189 (100.0)
Melanoma of the skin	17,997 (25.5)	15,841 (22.4)	13,514 (19.1)	11,186 (19.1)	12,152 (17.2)	70,690 (100.0)
Leukemia	12,989 (19.0)	13,801 (20.2)	14,245 (20.8)	12,869 (20.8)	14,587 (21.3)	68,491 (100.0)
Ovary	17,311 (25.5)	15,962 (23.5)	13,857 (20.4)	10,881 (20.4)	9822 (14.5)	67,833 (100.0)
Kidney and renal pelvis	15,816 (26.2)	14,639 (24.2)	12,807 (21.2)	9267 (21.2)	7914 (13.1)	60,443 (100.0)
Stomach	7483 (17.4)	8402 (19.6)	8921 (20.8)	8377 (20.8)	9732 (22.7)	42,915 (100.0)

^a^ Incidence data for 1975–1999 are from the Surveillance, Epidemiology and End Results (SEER) program: Incidence—SEER Research Data, 8 Registries, Nov 2022 Sub (1975–2020)—Linked To County Attributes. Incidence data for 2000–2020 are from the SEER program: Incidence—SEER Research Data, 17 Registries, Nov 2022 Sub (2000–2020)—Linked To County Attributes.

^b^ Ranked by decreasing incidence for males and females. The 12 sites listed respectively for male and female are those with the highest incidence (1975–2020).

^c^ Liver excludes intrahepatic bile duct.

### 3.3 Elderly cancer incidence, mortality, and survival trends

**FIGURE 2:**
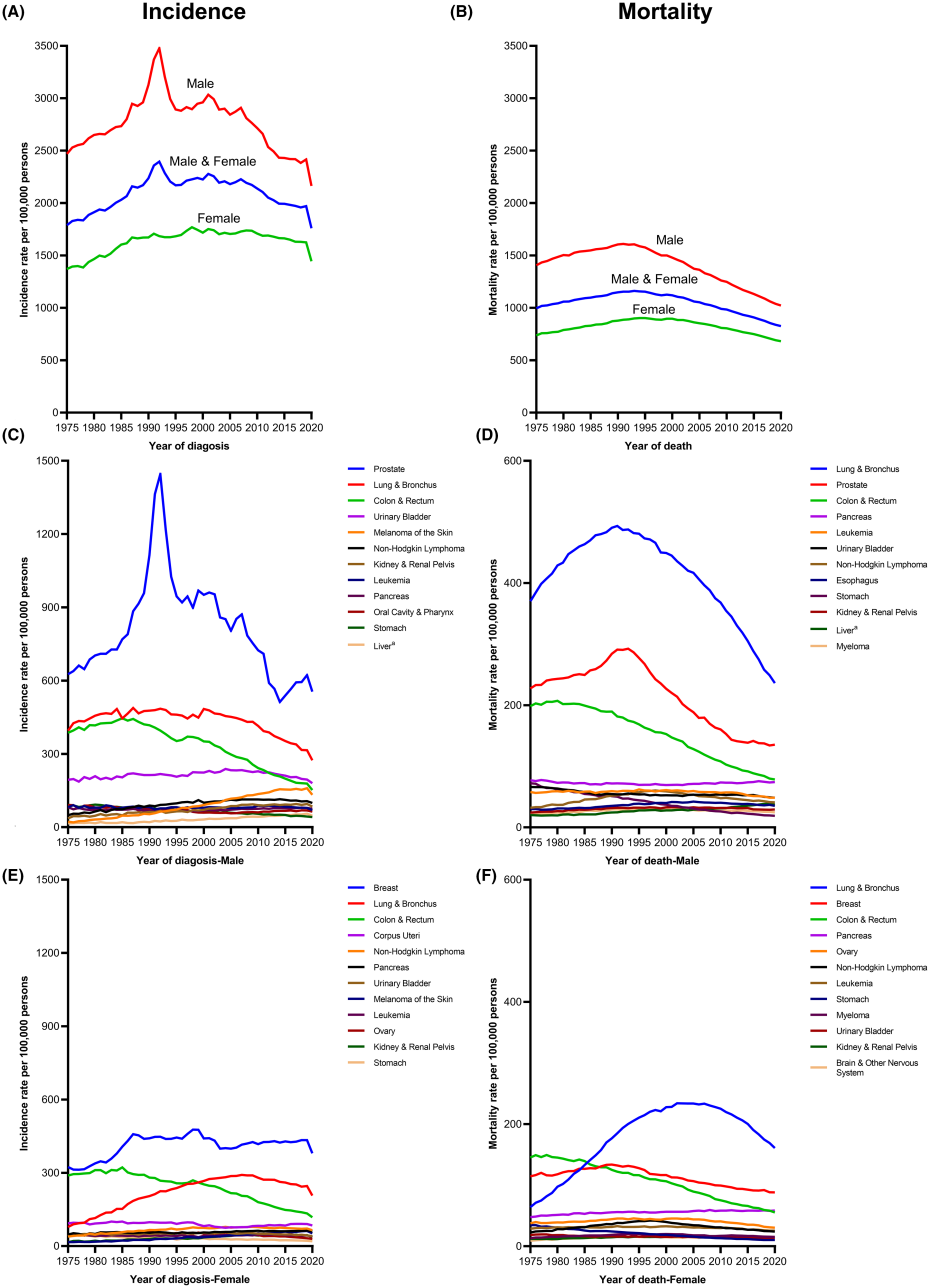
Trends in incidence and mortality rates per 100,000 persons (1975–2020, United States, Age‐Adjusted to the 2000 US standard population): (A) Overall cancer incidence trend by sex; (B) Overall cancer mortality trend by sex; (C) Incidence trend for cancers of the top 12 incidence in male; (D) Mortality trend for cancers of the top 12 mortality in male; (E) Incidence trend for cancers of the top 12 incidence in female; (F) Mortality trend for cancers of the top 12 mortality in female.

Overall cancer incidence rate increased by 1.617% per year during 1975–1991 and decreased by 1.341% per year during 2008–2020, from 1788.31 in 1975 to 1758.65 in 2020 per 100,000 elderly persons. While overall mortality rate decreased by 0.421% per year between 1975 and 2020, from 995.20 to 824.99 per 100,000 elderly persons (Tables S10 and S11). Total cancer incidence decreased between 2008 and 2020 (APC = −1.34*, 95% CI −1.61–1.07) and mortality decreased between 2002 and 2020 (APC = −1.55*, 95% CI −1.60–1.50), while men always had higher incidence and mortality than women (Figure 2).

**FIGURE 3:**
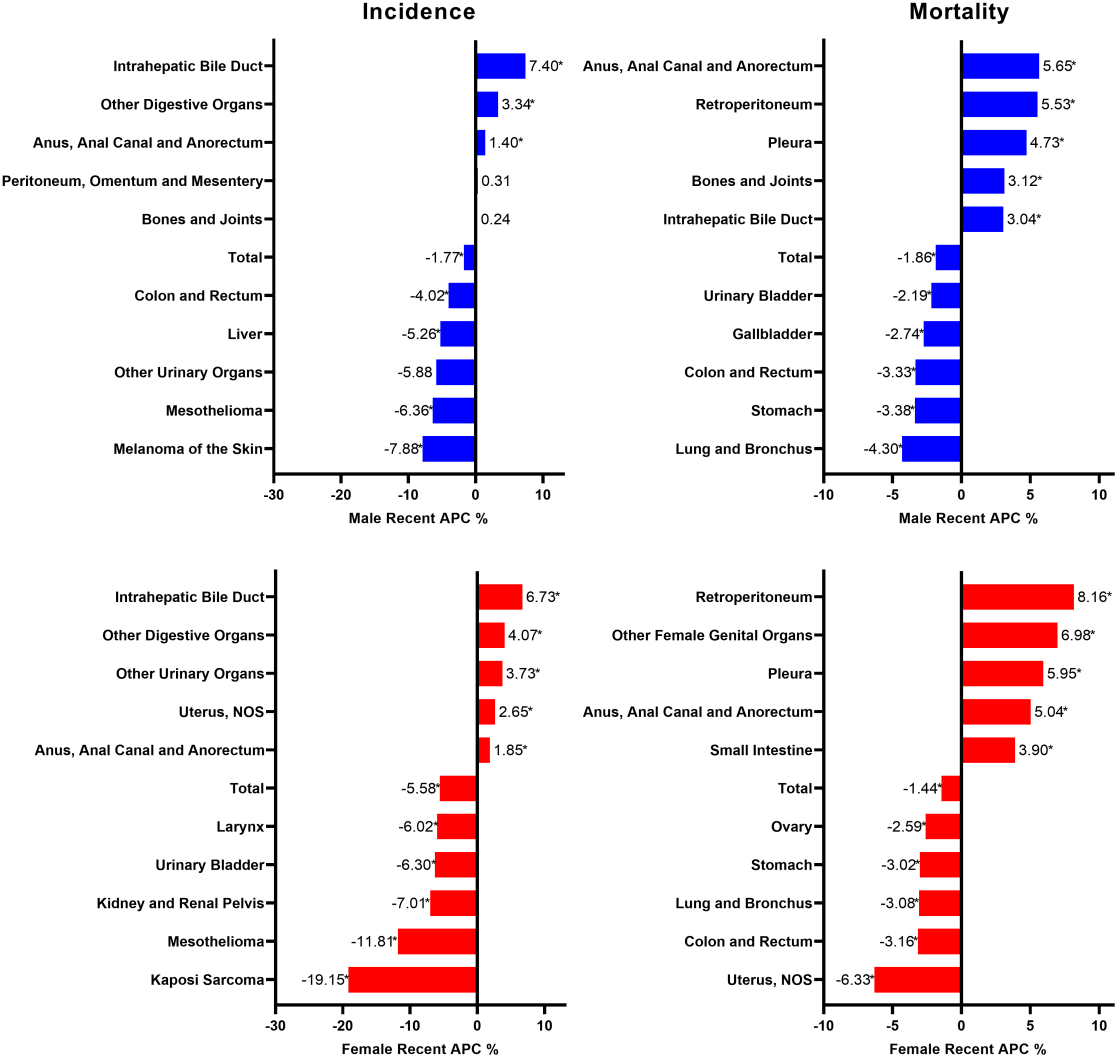
Annual Percentage Change (APC) of the cancers exhibiting the most rapid shifts in incidence or mortality rates during the recent follow‐up period.

Most cancers' incidence and mortality rates peaked during follow‐up time and declined in recent years (Tables 2 and 3, Tables S12 and S13). However, incidence for cancers of intrahepatic bile duct (male [2004–2020 APC = 7.4*, 95% CI 6.4–8.7]; female [2003–2020 APC = 6.7*, 95% CI 6.1–7.5]) and other digestive organs (male APC = 3.34*, 95% CI 2.77–4.39, 1996–2020; female APC = 4.07*, 95% CI 3.12–6.79, 2004–2020) increased fastest, and cancer of annus, anal canal, and anorectum in male (APC = 5.65*, 95% CI 4.34–8.02, 2008–2020) and retroperitoneum cancer in female (ACP = 8.16*, 95% CI 3.23–19.76, 2013–2020) were the malignancies with the highest increasing speed of mortality (Figure 3, Figures S2–S5).

**TABLE 2:** Annual Percentage Change (APC) and Average Annual Percentage Change (AAPC) for 12 cancers of the leading incidence rates by sex, United States, 1975–2020.

	Trend 1		Trend 2		Trend 3		Total
	Years	APC	Years	APC	Years	APC	1975–2020 AAPC
Overall							
Male & female	1975–1991	1.6174*	1991–2008	−0.2091	2008–2020	−1.3408*	0.1315
Male	1975–1991	1.6675*	1991–2007	−0.6313*	2007–2020	−1.7719*	−0.1535
Female	1975–1996	1.1193*	1996–2018	−0.2870*	2018–2020	−5.5803*	0.1238
Male							
Prostate	1975–1992	5.1326*	1992–2015	−3.2434*	2015–2020	0.1627	0.2239
Lung & bronchus	1975–2000	0.3024*	2000–2009	−1.1785*	2009–2020	−3.3687*	−0.9029*
Colon & rectum	1975–1985	1.2620*	1985–2002	−1.5601*	2002–2020	−4.0193*	−1.9372*
Urinary bladder	1975–2007	0.5857*	2007–2017	−1.2957*	2017–2020	−3.8683*	−0.1374*
Melanoma of the skin	1975–2007	5.1623*	2007–2018	2.1937*	2018–2020	−7.8809*	3.8160*
Non‐Hodgkin lymphoma	1975–1994	3.2592*	1994–2012	0.8998*	2012–2020	−1.5208*	1.4504*
Kidney & renal pelvis	1975–2009	2.3599*	2009–2020	−0.156			1.7391*
Leukemia	1975–2007	−0.1434	2007–2012	2.4849*	2012–2020	−2.2881*	−0.2405*
Pancreas	1975–1996	−0.6336*	1996–2018	0.8590*	2018–2020	−2.9182	−0.0101
Oral cavity & pharynx	1975–2005	−1.4963*	2005–2018	1.4652*	2018–2020	−3.6600*	−0.7477*
Stomach	1975–1980	0.3785	1980–2014	−1.6655*	2014–2020	−3.0203*	−1.6226*
Liver^a^	1975–2018	3.0195*	2018–2020	−5.2599*			2.6366*
Female							
Breast	1975–1990	3.0583*	1990–2005	−0.7518*	2005–2020	0.1249	0.7974*
Lung & bronchus	1975–1990	6.6032*	1990–2007	1.8905*	2007–2020	−1.9731*	2.2890*
Colon & rectum	1975–1983	0.8023	1983–2002	−1.2224*	2002–2020	−3.6909*	−1.8640*
Corpus uteri	1975–1997	0.0613	1997–2003	−3.8532*	2003–2020	1.0650*	−0.0937
Non‐Hodgkin lymphoma	1975–1991	3.1217*	1991–2008	0.9018*	2008–2020	−1.1451*	1.1314*
Pancreas	1975–2001	0.2750*	2001–2008	1.3512*	2008–2020	0.0334	0.3771*
Urinary bladder	1975–2004	0.4546*	2004–2018	−1.1307*	2018–2020	−6.3027*	−0.3502*
Melanoma of the skin	1975–2007	3.4500*	2007–2018	2.5513*	2018–2020	−5.8627*	2.7977*
Leukemia	1975–1999	−0.2889	1999–2013	1.0499*	2013–2020	−2.1110*	−0.1612*
Ovary	1975–1990	1.4116*	1990–2009	−0.9924*	2009–2020	−3.4260*	−0.8028*
Kidney & renal pelvis	1975–2007	2.8085*	2007–2018	0.1258	2018–2020	−7.0103*	1.6915*
Stomach	1975–2016	−1.3981*	2016–2020	−3.8355*			−1.6172*

* Indicate that the Annual Percentage Change (APC) and Average Annual Percentage Change (AAPC) are significantly different from zero at the alpha = 0.05 level (*p* value is not available for the Empirical Quantile method).

**TABLE 3:** Annual Percentage Change (APC) and Average Annual Percentage Change (AAPC) for 12 cancers of the leading mortality rates by sex, United States, 1975–2020.

	Trend 1		Trend 2		Trend 3		Total
	Years	APC	Years	APC	Years	APC	1975–2020 AAPC
Overall							
Male & female	1975–1992	0.8554*	1992–2002	−0.5376*	2002–2020	−1.5477*	−0.4210*
Male	1975–1992	0.7355*	1992–2002	−1.2109*	2002–2020	−1.8626*	−0.7432*
Female	1975–1993	1.0796*	1993–2003	−0.2268*	2003–2020	−1.4410*	−0.1691*
Male							
Lung & bronchus	1975–1991	1.6886*	1991–2009	−1.4565*	2009–2020	−4.2972*	−1.0595*
Prostate	1975–1993	1.5081*	1993–2013	−3.5756*	2013–2020	−0.6626*	−1.1166*
Colon & rectum	1975–1985	0.029	1985–2000	−1.9107*	2000–2020	−3.3310*	−2.1195*
Pancreas	1975–1983	−0.8661*	1983–2000	−0.2127	2000–2020	0.3618*	−0.0745*
Leukemia	1975–2000	0.2184*	2000–2012	−0.5290*	2012–2020	−2.0737*	−0.3920*
Urinary bladder	1975–1989	−1.6515*	1989–2016	−0.0815*	2016–2020	−2.1918*	−0.7611*
Non‐Hodgkin lymphoma	1975–1990	3.3364*	1990–1998	1.9371*	1998–2020	−1.7799*	0.5586*
Esophagus	1975–2001	1.4378*	2001–2006	0.221	2006–2020	−1.0555*	0.5206*
Stomach	1975–1988	−2.5194*	1988–1991	−0.5701	1991–2020	−3.3782*	−2.9457*
Kidney & renal pelvis	1975–1991	1.7624*	1991–2011	0.0046	2011–2020	−1.3229*	0.3575*
Liver^a^	1975–1997	1.9094*	1997–2006	0.3424	2006–2020	2.3609*	1.7339*
Myeloma	1975–1994	1.7957*	1994–2020	−0.7023*			0.3449*
Female							
Lung & bronchus	1975–1992	6.6271*	1992–2008	1.0674*	2008–2020	−3.0819*	1.9863*
Breast	1975–1991	1.0258*	1991–2009	−1.6419*	2009–2020	−1.1153*	−0.5719*
Colon & rectum	1975–1984	−0.7145*	1984–2000	−1.7412*	2000–2020	−3.1583*	−2.1704*
Pancreas	1975–1984	1.2764*	1984–2010	0.2906*	2010–2020	0.0126	0.4251*
Ovary	1975–1992	1.0952*	1992–2006	−0.0105	2006–2020	−2.5943*	−0.4087*
Non‐Hodgkin lymphoma	1975–1989	3.2905*	1989–1997	1.9785*	1997–2020	−2.4438*	0.0910*
Leukemia	1975–2000	0.2965*	2000–2013	−0.8118*	2013–2020	−2.0216*	−0.3880*
Stomach	1975–1986	−2.9815*	1986–1991	−0.9936	1991–2020	−3.0201*	−2.7876*
Myeloma	1975–1992	2.0149*	1992–2000	−0.0409	2000–2020	−1.1271*	0.2429*
Urinary bladder	1975–1986	−1.5182*	1986–2016	−0.4122	2016–2020	−2.3897*	−0.8606*
Kidney & renal pelvis	1975–1992	1.6746*	1992–2006	0.1705	2006–2020	−1.5531*	0.1936*
Brain & other nervous system	1975–1991	2.8225*	1991–2006	−0.5845*	2006–2020	0.5071*	0.9562*

* Indicate that the Annual Percentage Change (APC) and Average Annual Percentage Change (AAPC) are significantly different from zero at the alpha = 0.05 level (*p* value is not available for the Empirical Quantile method).

**FIGURE 4:**
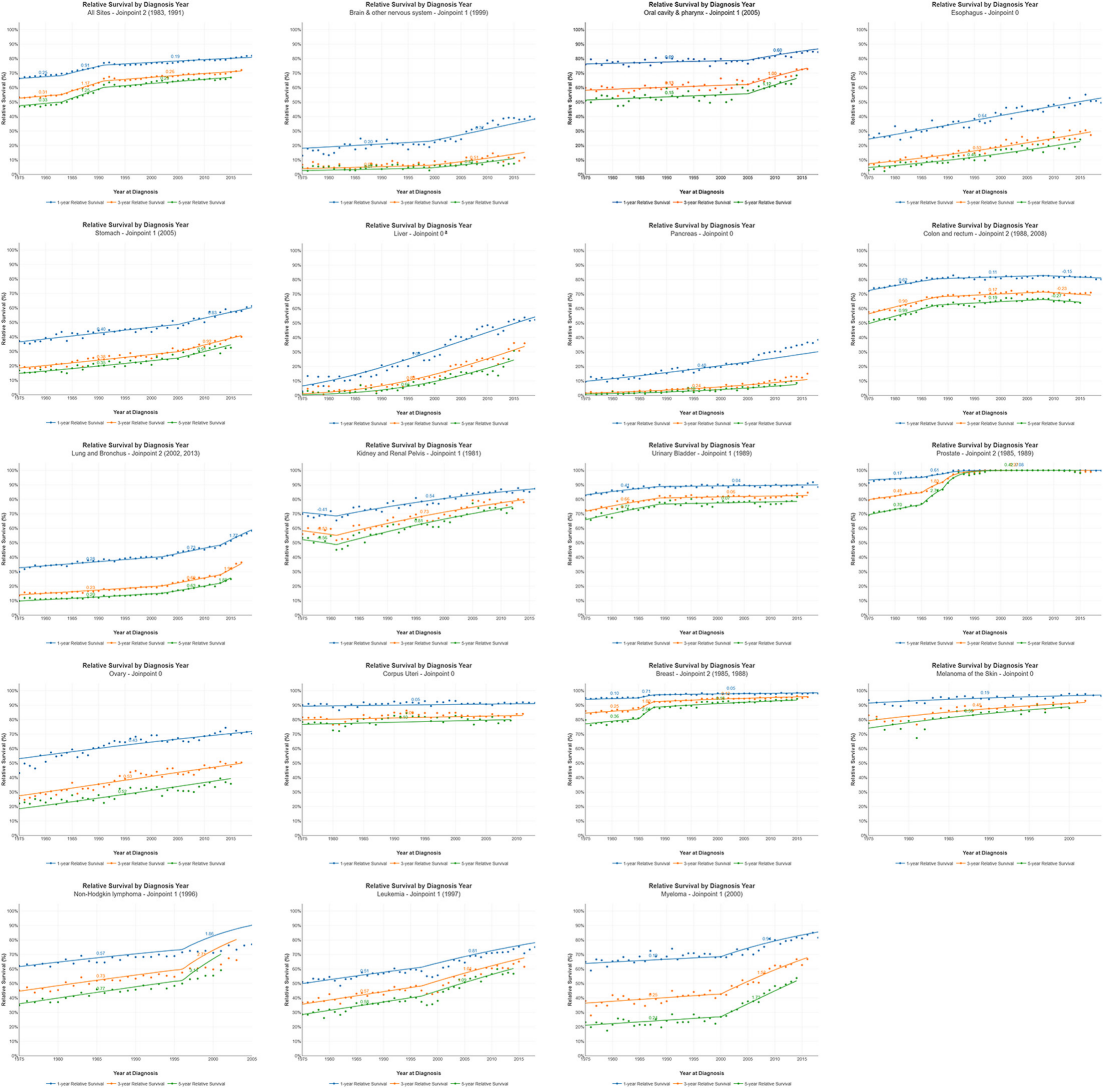
Trends in 1‐, 3‐ and 5‐year relative survival for all‐sites and 18 cancers with top 12 incidence or mortality in male or female, United States, 1975–2019.

The 18 cancers with the highest incidence or mortality rates showed similar trends in 1‐, 3‐, and 5‐year relative survival, all of which improved during 1975–2020 (Figure 4). Note that survival for cancer of colon and rectum peaked in 2008 and then decreased during 2008–2019 (1‐year AAC_S = −0.15*; 3‐year AAC_S = −0.23*; 5‐year AAC_S = −0.27*; Figure S6 and Table S14). And survival for cancer of other female genital organs had decreased since 1991 (1‐year AAC_S = −0.31*; 3‐year AAC_S = −0.51*; 5‐year AAC_S = −0.58*), for ureter cancer since 1975 (1‐year AAC_S = −0.27*; 3‐year AAC_S = −0.42*; 5‐year AAC_S = −0.46*), and for cancers of other urinary organs since 2014 (1‐year AAC_S = −3.96*; 3‐year AAC_S = −4.95*; 5‐year AAC_S = −5.01*).

### 3.4 Elderly cancer stage distribution

**FIGURE 5:**
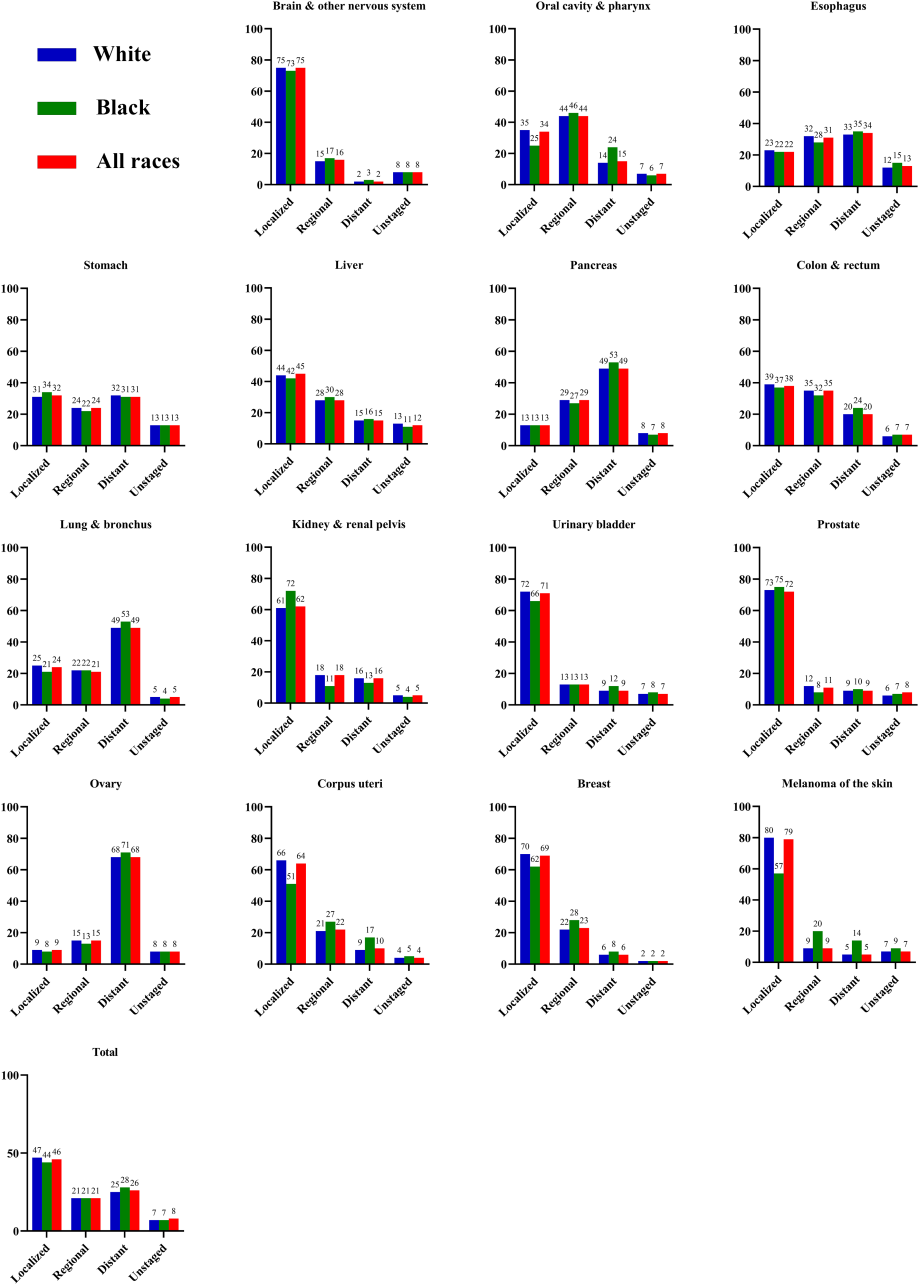
Stage distribution for all‐sites and cancers with top 12 incidence or mortality in male or female, United States, 2010–2020, excluding blood cancers.

Cancers of brain and other nervous system (75%), liver (45%), colon and rectum (38%), kidney and renal pelvis (62%), urinary bladder (71%), prostate (72%), corpus uteri (64%), breast (69%), and melanoma of the skin (79%) had higher proportion of localized stage at diagnosis during 2010–2020 (Figure 5). While cancers of pancreas (49%), lung and bronchus (49%), and ovary (68%) had a higher proportion of distant stage. And Black patients had a higher proportion of distant‐stage cancers of oral cavity and pharynx (24%), pancreas (53%), colon and rectum (24%), lung and bronchus (53%), and corpus uteri (17%).

## 4 DISCUSSION

In this cross‐sectional study, we found that cancers affecting the elderly were considerably discrepancy from the younger ones during 1975–2020.^17^ Overall cancer incidence rate increased during 1975–1991 and decreased during 2008–2020. While overall mortality rate had decreased since 1975. And relative survival of most cancers increased steadily during 1975–2019. Though overarching trends were favorable, some cancers turned adversely. These findings are important for early cancer detection strategy‐making and selection of government investment priorities in the elderly.^18^

Most previous studies focused on cancer trends in general or more specific populations.^18^, ^19^ This study was conducted on the background that persons aged 65 years or more were the majority of cancer patients.^3^ The results in the early follow‐up periods of our study were in line with previous studies, which were conducted using data from more limited periods,^20^, ^21^, ^22^ and updated data was additionally analyzed in this present study.

Compared with the studies from Global Burden of Disease Study 2019 database, which is the largest and most comprehensive effort to quantify health loss across places and over time,^23^ lung cancer had the highest disability‐adjusted life years globally, and it was the most common cancer death in the US.^24^ For colorectal cancer, incidence and mortality for all‐age population increased since 1990 globally. But in this study, incidence and death of colorectal cancer had decreased since 1990 in the elderly people in the US.^25^

Cancer is a complex disease related to many factors, including genetics, lifestyle choices, environmental exposures, and infections. Social determinants of health on cancer risk include socioeconomic status, education, neighborhood and physical environment, employment, and social support networks, as well as access to healthcare.^26^ The trends in cancer epidemiology often reflect variations in these risk factors. For instance, the incidence of liver cancer rose at first and then began to decline, influenced by advancements in the treatment of hepatitis C virus (HCV). After the World Health Organization (WHO) adopted a strategy in 2014 to eliminate viral hepatitis as a global health threat by 2030, significant progress was made, contributing to the reduction in liver cancer rates.^27^

The cancer incidence rate in the elderly peaked at the age of 80–84 years, then declined after 85 years old (usually defined as oldest‐old). As the poor health conditions and limited life expectancy, physicians usually avoid complicated detection strategies, which may lead to the underestimation of cancer diagnosis in the oldest old.^28^, ^29^ Elderly males always had a higher proportion of cancer incidence than females, except for those aged 85 years or more, which was largely due to the increased cancer of colon and rectum, urinary bladder, stomach, and leukemia in females. The mortality rate in the elderly increased as people aged. Males also had a higher proportion of mortality than females except for those aged 80 years or more, which were attributed to the increased deaths in many cancer types after 85 years old in female, including cancer of breast, colon and rectum, pancreas, etc. Populations aged 85 years or more showed unique cancer distribution, which resulted in them being classified as a special research group.^9^, ^30^

The cancer incidence rate of elderly males was approximately 1.5 times that of females, similar for mortality, which was greatly attributed to gender differences in genetic predispositions and hazardous lifestyle choices (e.g., tobacco smoking, alcohol consumption).^31^ Sex disparities became prominent as people got older. Diagnoses of 13 cancers in males and 18 cancers in females, and mortality of 14 cancers in men and 15 cancers in women increased significantly during 1975–2020. Males showed a steeper decrease in both overall cancer incidence and mortality. The gender of patients with non‐productive cancers had not been adequately considered during cancer curing until 2016 when the US National Institutes of Health introduced a policy changes towards genders for medical research.^32^ It is time to optimize cancer treatment strategies in men and women.

Cancers most frequent in the elderly were prostate (15.6%), lung and bronchus (15.2%), and breast (11.8%) malignancies during 1975–2020. Cancers with the highest incidence for the elderly were prostate (14.8%), lung and bronchus (13.6%), and breast (12.4%) in 2020, while the sequence was breast (15.3%), lung and bronchus (12.7%), and prostate (10.6%) cancers in the general population.^33^, ^34^ Cancers caused most deaths in the elderly were lung and bronchus (26.6%), colon and rectum (11.2%), and prostate (7.3%) during 1975–2020. And cancer of lung and bronchus (23.5%), pancreas (8.0%), and colon and rectm (8.0%) had the top three mortality in the elderly in 2020, compared with lung and bronchus (22.7%), colon and rectum (8.8%), and breast (7.0%) in the general population.^34^ These changes in cancer distribution could provide reference to detection strategies and challenge healthcare institutions in future.

Although the overall elderly cancer incidence rate increased with no significance during 1975–2020, many cancer types showed significant increasing trends, including cancer of small intestinal, kidney and renal pelvis, endocrine system, melanoma, non‐Hodgkin lymphoma, etc. Females had 18 cancer types with increasing incidence compared to 13 in males. Cancer of peritoneum, omentum, and mesentery in elderly females increased fastest but could play little role in the overall incidence due to its low absolute incidence. Most peritoneum, omentum, and mesentery cancer was metastatic, and most primary sites were ovary and fallopian tubes, which may lead to the increasing incidence in females.^35^ Our study also found high metastasis rate in ovary cancer, with 68% distant stage at diagnosis.^36^ Therefore, physicians should attach importance to peritoneum, omentum, and mesentery screening after discovering primary malignancies.

Most cancer incidence and mortality rates decreased in the latest follow‐up time. While for intrahepatic cholangiocarcinoma (iCCA), incidence increased fast during 2004–2020, and mortality and survival was also relatively terrible (3‐year survival only 10%), which calls for earlier detection of the disease.^37^, ^38^, ^39^ Previous studies also found a greater increase in iCCA versus extrahepatic cholangiocarcinoma during 2001–2017.^40^ However, misclassification of perihilar cholangiocarcinoma as iCCA should not be ignored in the ICD system, which may cause overestimation of the increasing incidence for iCCA.^40^, ^41^

Cancer of anus, anal canal, and anorectum had similar increasing incidence and mortality rates as iCCA, but survival was relatively well.^42^ That may attribute to the increasing infection of human papillomavirus due to adverse sexuality (e.g., anal intercourse) and HIV‐related immunosuppression.^43^ Other cancers with increasing incidence in the latest follow‐up period include cancer of other digestive organs in both genders and cancer of corpus uteri, other urinary organs, and pancreas in females.

Overall cancer mortality in the elderly decreased in the past two decades. Cancer in males decreased faster than females in the nearest research time. But a total of 18 cancers in elderly males (e.g., liver, anus, bile duct, retroperitoneum, pleura, peritoneum, bone and joints, etc.) and 16 cancers in elderly females (e.g., small intestine, anus, intrahepatic bile duct, retroperitoneum, pleura, bone and joints, other genital organs, etc.) showed upward mortality trend in the latest follow‐up periods. The increasing incidence and mortality were partly attributed to more depression, anxiety, obesity, hazardous lifestyles, and higher levels of Human Development Index of contemporary people, especially in high‐income countries such as the United States.^44^, ^45^, ^46^, ^47^

A steep drop was observed in cancer incidence in 2020, and many cancers showed similar trends, such as cancer of prostate, lung and bronchus, breast, melanoma, etc. Coronavirus disease 2019 (COVID‐19) broke out in China at the end of 2019 and immediately spread worldwide. Previous studies indicated that the restriction measure of COVID‐19 may lead to the limitation of cancer diagnosis or diagnostic tests.^48^ Studies also found that cancer patients had a higher risk of COVID‐19 infection and developing severe events.^49^, ^50^ However, we did not observe an increase in mortality or a decrease in survival, likely due to the short interval between COVID‐19 diagnosis and the end of the cohort observation period.

Black individuals had a 3% higher proportion of distant stage at diagnosis than White in the elderly during 2010–2020, especially in cancer of oral cavity and pharynx (24% vs. 14%), corpus uteri (17% vs. 9%), and melanoma (14% vs. 5%). Previous research found that African American/Black individuals had the highest mortality and lowest survival for most cancers compared with any racial/ethnic group, which was driven by the lower socioeconomic status of Blacks.^51^, ^52^ The elderly cancer patients diagnosed with unstaged stage were similar between White and Black individuals, which partly revealed the efforts made by the US government to reduce racial inequalities.^53^

With advances in cancer diagnosis and treatment, the detection of cancers at an early stage has improved, subsequently enhancing survival rates. For instance, endoscopy has become a crucial tool in identifying early‐stage cancers of the esophagus and stomach. It is advised that individuals over the age of 40 should undergo biennial endoscopic examinations.^54^ Additionally, in Japan, insurance policies now cover screening and treatment for H. pylori, a known risk factor for stomach cancer, demonstrating a proactive approach in public health management and cancer prevention.^55^

There are several limitations of this study. At first, we used the database covering larger population to obtain as accurate statistics as possible, and the mortality data covered a wider US population than incidence data; therefore, there were more deaths than diagnoses during the follow‐up periods. Secondly, SEER is a large population‐based database, and misclassification of race/ethnicity is unavoidable. Thirdly, death included data from autopsy or death certificate, which can lead to the misclassification of cancer death, particularly among the elderly population with multiple comorbidities. Forthly, we set the maximum number of joinpoints to 2 instead of the recommending number of 7, because that may cause the difficulty of trend description based on so many types of cancer, and deviation was unavoidable due to this adjustment. And SEER database lacks detailed information such as molecular pathological characteristics, socioeconomic status, and treatment (needs request), which limited our findings.

## 5 CONCLUSIONS

The elderly populations are the majority of cancer patients, while long‐term epidemiology research was limited. In this cross‐sectional study, the rate of cancer incidence increased with no significance while mortality rate declined by 0.42% annually from 1975 to 2020. Prostate and breast cancer were the most common malignancies in elderly males and females, respectively. And cancer of lung and bronchus showed the highest mortality rate. Overall incidence, mortality and survival improved, while incidence of intrahepatic cholangiocarcinoma and anus, anal canal, and anorectum cancer increased fast in the last period calculated by the Joinpoint method. It is time to optimize targeted cancer‐curing medical strategies towards site‐specific cancers and demographic‐specific populations in the elderly.

## AUTHOR CONTRIBUTIONS

**Jia Xu:** Data curation (lead); formal analysis (lead); methodology (lead); resources (equal); software (lead); validation (equal); visualization (lead); writing – original draft (lead). **Jingyuan Liao:** Project administration (equal); supervision (equal); validation (equal). **Qiong Yan:** Project administration (equal); supervision (equal). **Jiang Jiao:** Formal analysis (equal); methodology (equal); software (equal). **Nan Hu:** Methodology (equal); software (equal). **Wei Zhang:** Supervision (equal). **Lei Shi:** Supervision (equal). **Mingming Deng:** Supervision (equal). **Shu Huang:** Supervision (equal). **Xiaowei Tang:** Conceptualization (lead); funding acquisition (equal); resources (equal); supervision (equal); writing – original draft (equal); writing – review and editing (equal).

## FUNDING INFORMATION

None.

## CONFLICT OF INTEREST STATEMENT

Dr. Jia Xu, Dr. Jingyuan Liao, Dr. Qiong Yan, Dr. Jiao Jiang, Dr. Nan Hu, Dr. Wei Zhang, Dr. Lei Shi, Dr. Mingming Deng, Dr. Shu Huang, Dr. Xiaowei Tang all have no conflicts of interest or financial ties to disclose.

## ETHICS STATEMENT

SEER database is a publicly available database, therefore ethical approval and informed consent are not required. We signed a contract with SEER database and received permission to use the data for non‐commercial purpose.

## Supporting information


Appendix S1.

## Data Availability

The data used in this population‐based study are provided by the Surveillance, Epidemiology and End Results (SEER) database (https://seer.cancer.gov/) and National Center for Health Statistics (NCHS, www.cdc.gov/nchs), both of which are publicly available. As the deidentified data and public availability, this study was exempted from informed consent and institutional review board approval. The data that supports these findings are available in the public SEER database using SEER*stat software (version 8.4.2).
