# Supplementary material for: Trends analysis of cancer incidence, mortality, and survival for the elderly in the United States, 1975–2020
Source: Cancer Med. 2024 Jul 31;13(15):e70062. doi: 10.1002/cam4.70062 (PMC11289898; doi:10.1002/cam4.70062)
Supplement: Supplementary file 1 — Appendix S1. [file CAM4-13-e70062-s001.zip › Supplementary Figure 3 Trends in mortality rates p.docx]

**Supplementary Figure 3** Trends in mortality rates per 100,000 persons for the leading 12 cancers by sex, United States, 1975-2020. Rates are age adjusted to the 2000 US standard population. ^a^Liver excludes intrahepatic bile duct.

**
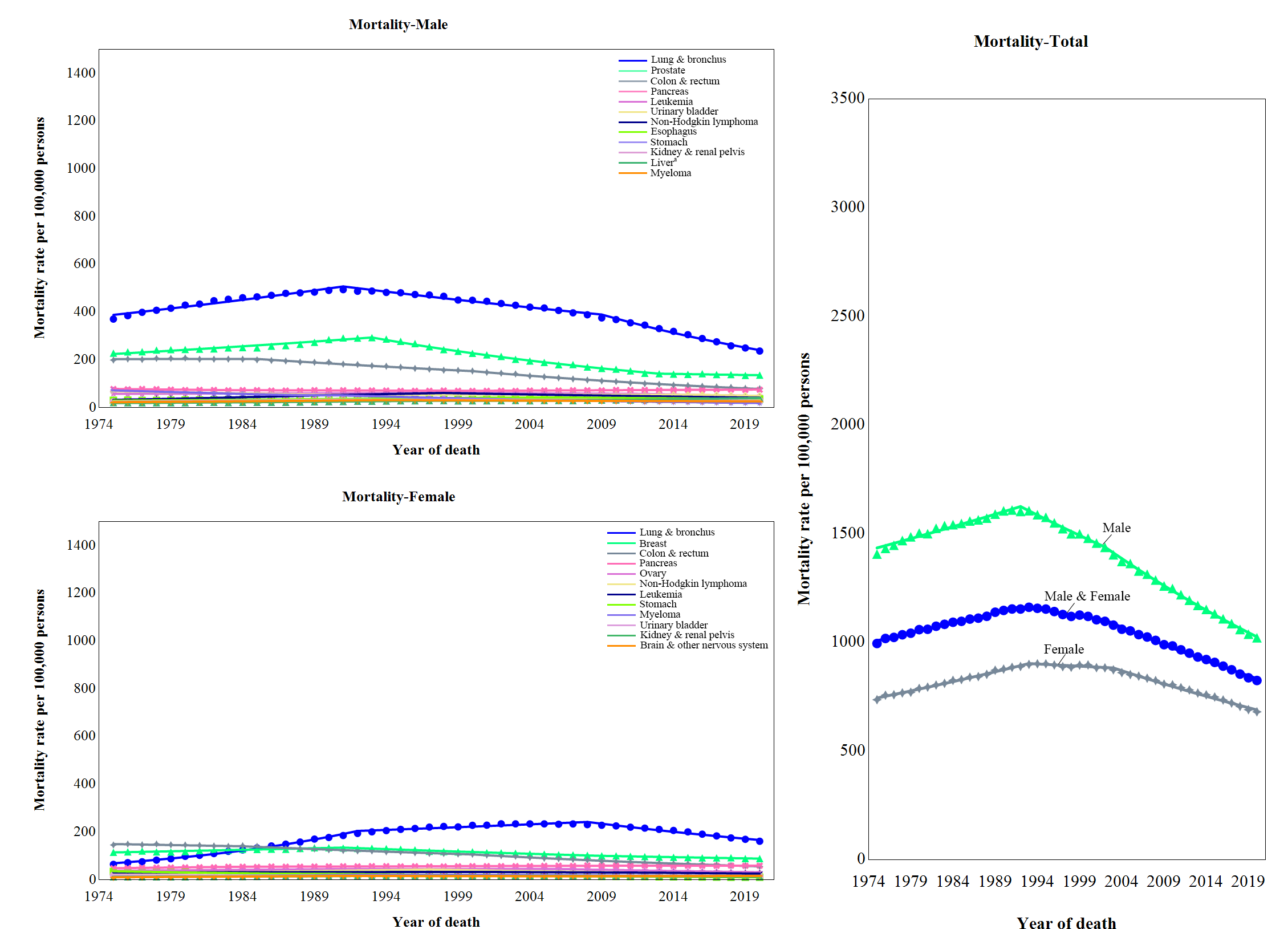
**
