# Supplementary material for: Trends analysis of cancer incidence, mortality, and survival for the elderly in the United States, 1975–2020
Source: Cancer Med. 2024 Jul 31;13(15):e70062. doi: 10.1002/cam4.70062 (PMC11289898; doi:10.1002/cam4.70062)

**Supplementary Figure 5** Trends in mortality rates per 100,000 persons for the rest cancers by sex, United States, 1975-2020. Rates are age adjusted to the 2000 US standard population.


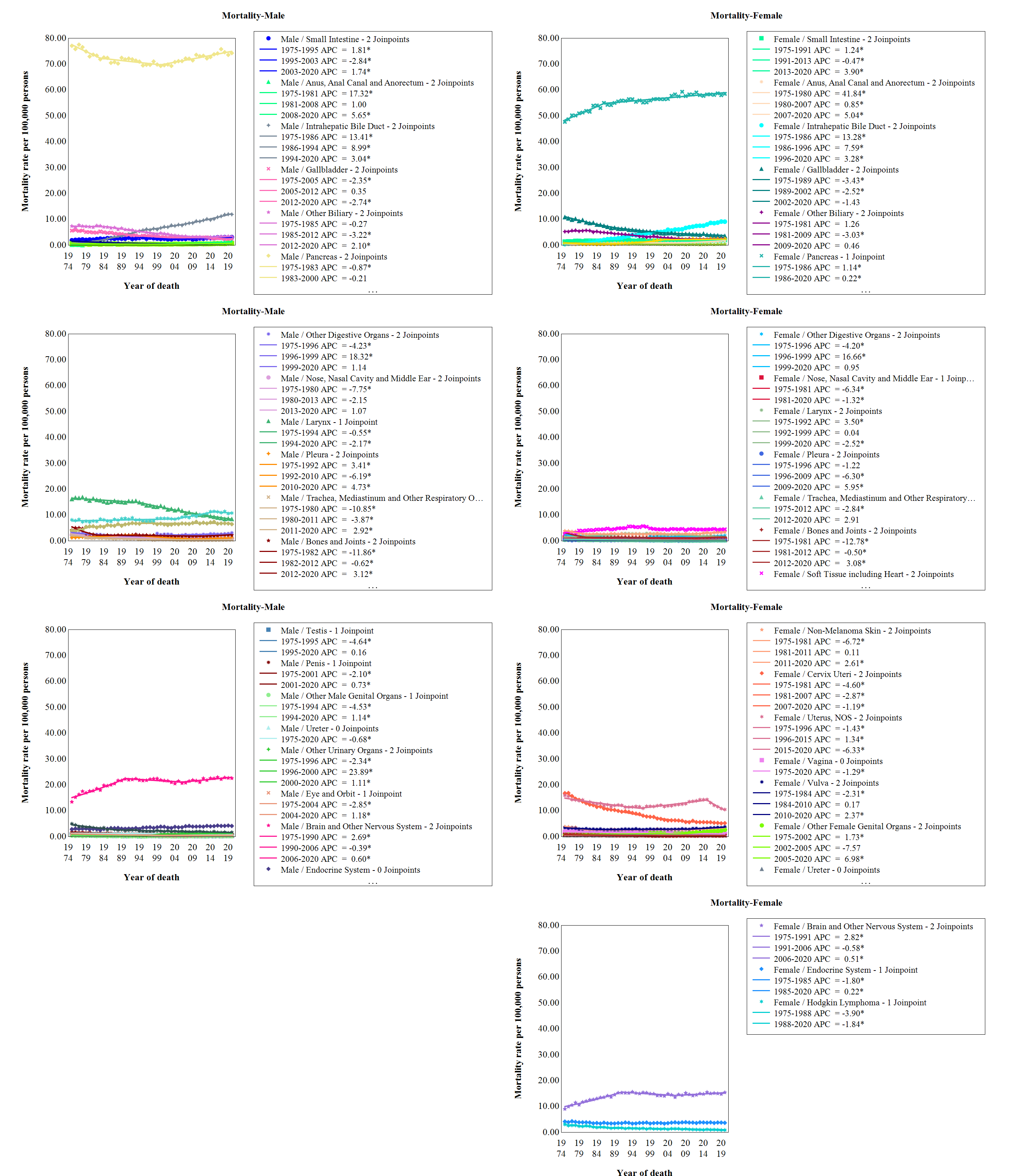

Supplement: Supplementary file 1 — Appendix S1. [file CAM4-13-e70062-s001.zip › Supplementary Figure 5 Trends in mortality rates p.docx]
