# Supplementary material for: Trends analysis of cancer incidence, mortality, and survival for the elderly in the United States, 1975–2020
Source: Cancer Med. 2024 Jul 31;13(15):e70062. doi: 10.1002/cam4.70062 (PMC11289898; doi:10.1002/cam4.70062)
Supplement: Supplementary file 1 — Appendix S1. [file CAM4-13-e70062-s001.zip › Supplementary Table 1 ICD.docx]

**Supplementary Table 1 ICD-O-3/WHO 2008 site code.**

|  | ICD-O-3 Site | ICD-O-3 Histology (Type) | Recode |
| --- | --- | --- | --- |
| **Oral Cavity and Pharynx** | | | |
| Lip | C000-C009 | excluding 9050-9055, 9140, 9590-9993 | 20010 |
| Tongue | C019-C029 |  | 20020 |
| Salivary Gland | C079-C089 |  | 20030 |
| Floor of Mouth | C040-C049 |  | 20040 |
| Gum and Other Mouth | C030-C039, C050-C059, C060-C069 |  | 20050 |
| Nasopharynx | C110-C119 |  | 20060 |
| Tonsil | C090-C099 |  | 20070 |
| Oropharynx | C100-C109 |  | 20080 |
| Hypopharynx | C129, C130-C139 |  | 20090 |
| Other Oral Cavity and Pharynx | C140, C142, C148 |  | 20100 |
| **Digestive System** | | | |
| Esophagus | C150-C159 | excluding 9050-9055, 9140, 9590-9993 | 21010 |
| Stomach | C160-C169 |  | 21020 |
| Small Intestine | C170-C179 |  | 21030 |
| Colon and Rectum | | | |
| Colon excluding Rectum | | | |
| Cecum | C180 | excluding 9050-9055, 9140, 9590-9993 | 21041 |
| Appendix | C181 |  | 21042 |
| Ascending Colon | C182 |  | 21043 |
| Hepatic Flexure | C183 |  | 21044 |
| Transverse Colon | C184 |  | 21045 |
| Splenic Flexure | C185 |  | 21046 |
| Descending Colon | C186 |  | 21047 |
| Sigmoid Colon | C187 |  | 21048 |
| Large Intestine, NOS | C188-C189, C260 |  | 21049 |
| Rectum and Rectosigmoid Junction | | | |
| Rectosigmoid Junction | C199 | excluding 9050-9055, 9140, 9590-9993 | 21051 |
| Rectum | C209 |  | 21052 |
| Anus, Anal Canal and Anorectum | C210-C212, C218 |  | 21060 |
| Liver and Intrahepatic Bile Duct | | | |
| Liver | C220 | excluding 9050-9055, 9140, 9590-9993 | 21071 |
| Intrahepatic Bile Duct | C221 |  | 21072 |
| Gallbladder | C239 |  | 21080 |
| Other Biliary | C240-C249 |  | 21090 |
| Pancreas | C250-C259 |  | 21100 |
| Retroperitoneum | C480 |  | 21110 |
| Peritoneum, Omentum and Mesentery | C481-C482 |  | 21120 |
| Other Digestive Organs | C268-C269, C488 |  | 21130 |
| **Respiratory System** | | | |
| Nose, Nasal Cavity and Middle Ear | C300-C301, C310-C319 | excluding 9050-9055, 9140, 9590-9993 | 22010 |
| Larynx | C320-C329 |  | 22020 |
| Lung and Bronchus | C340-C349 |  | 22030 |
| Pleura | C384 |  | 22050 |
| Trachea, Mediastinum and Other Respiratory Organs | C339, C381-C383, C388, C390, C398, C399 |  | 22060 |
| **Bones and Joints** | C400-C419 | excluding 9050-9055, 9140, 9590-9993 | 23000 |
| **Soft Tissue including Heart** | C380, C470-C479, C490-C499 | excluding 9050-9055, 9140, 9590-9993 | 24000 |
| **Skin excluding Basal and Squamous** | | | |
| Melanoma of the Skin | C440-C449 | 8720-8790 | 25010 |
| Other Non-Epithelial Skin | C440-C449 | excluding 8000-8005, 8010-8046, 8050-8084, 8090-8110, 8720-8790, 9050-9055, 9140, 9590-9993 | 25020 |
| **Breast** | C500-C509 | excluding 9050-9055, 9140, 9590-9993 | 26000 |
| **Female Genital System** | | | |
| Cervix Uteri | C530-C539 | excluding 9050-9055, 9140, 9590-9993 | 27010 |
| Corpus and Uterus, NOS | | | |
| Corpus Uteri | C540-C549 | excluding 9050-9055, 9140, 9590-9993 | 27020 |
| Uterus, NOS | C559 |  | 27030 |
| Ovary | C569 |  | 27040 |
| Vagina | C529 |  | 27050 |
| Vulva | C510-C519 |  | 27060 |
| Other Female Genital Organs | C570-C579, C589 |  | 27070 |
| **Male Genital System** | | | |
| Prostate | C619 | excluding 9050-9055, 9140, 9590-9993 | 28010 |
| Testis | C620-C629 |  | 28020 |
| Penis | C600-C609 |  | 28030 |
| Other Male Genital Organs | C630-C639 |  | 28040 |
| **Urinary System** | | | |
| Urinary Bladder | C670-C679 | excluding 9050-9055, 9140, 9590-9993 | 29010 |
| Kidney and Renal Pelvis | C649, C659 |  | 29020 |
| Ureter | C669 |  | 29030 |
| Other Urinary Organs | C680-C689 |  | 29040 |
| **Eye and Orbit** | C690-C699 | excluding 9050-9055, 9140, 9590-9993 | 30000 |
| **Brain and Other Nervous System** | | | |
| Brain | C710-C719 | excluding 9050-9055, 9140, 9530-9539, 9590-9993 | 31010 |
| Cranial Nerves Other Nervous System | C710-C719 | 9530-9539 | 31040 |
|  | C700-C709, C720-C729 | excluding 9050-9055, 9140, 9590-9993 |  |
| **Endocrine System** | | | |
| Thyroid | C739 | excluding 9050-9055, 9140, 9590-9993 | 32010 |
| Other Endocrine including Thymus | C379, C740-C749, C750-C759 |  | 32020 |
| **Lymphoma** | | | |
| Hodgkin Lymphoma | | | |
| Hodgkin - Nodal | C024, C098-C099, C111, C142, C379, C422, C770-C779 | 9650-9667 | 33011 |
| Hodgkin - Extranodal | All other sites |  | 33012 |
| Non-Hodgkin Lymphoma | | | |
| NHL - Nodal | C024, C098, C099, C111, C142, C379, C422, C770-C779 | 9590-9597, 9670-9671, 9673, 9675, 9678-9680, 9684, 9687-9691, 9695, 9698-9702, 9705, 9708-9709, 9712, 9714-9719, 9724-9729, 9735, 9737-9738, 9811-9818, 9823, 9827, 9837 | 33041 |
| NHL - Extranodal | All sites except C024, C098-C099, C111, C142, C379, C422, C770-C779 | 9590-9597, 9670-9671, 9673, 9675, 9678-9680, 9684, 9687, 9688, 9689-9691, 9695, 9698-9702, 9705, 9708-9709, 9712, 9714-9719, 9724-9729, 9735, 9737, 9738 | 33042 |
|  | All sites except C024, C098-C099, C111, C142, C379, C420-C422, C424, C770-C779 | 9811-9819, 9823, 9827, 9837 |  |
| **Myeloma** |  | 9731-9732, 9734 | 34000 |
| **Leukemia** | | | |
| Lymphocytic Leukemia | | | |
| Acute Lymphocytic Leukemia |  | 9826, 9835-9836 | 35011 |
|  | C420, C421, C424 | 9811-9819, 9837 |  |
| Chronic Lymphocytic Leukemia | C420, C421, C424 | 9823 | 35012 |
| Other Lymphocytic Leukemia |  | 9820, 9832-9834, 9940 | 35013 |
| Myeloid and Monocytic Leukemia | | | |
| Acute Myeloid Leukemia |  | 9840, 9861, 9865-9867, 9869, 9871-9874, 9877-9879, 9895-9897, 9898, 9910-9912, 9920 | 35021 |
| Acute Monocytic Leukemia |  | 9891 | 35031 |
| Chronic Myeloid Leukemia |  | 9863, 9875-9876, 9945-9946 | 35022 |
| Other Myeloid/Monocytic Leukemia |  | 9860, 9930 | 35023 |
| Other Leukemia | | | |
| Other Acute Leukemia |  | 9801, 9805-9809, 9931 | 35041 |
| Aleukemic, subleukemic and NOS |  | 9733, 9742, 9800, 9831, 9870, 9948, 9963-9964 | 35043 |
|  | C420, C421, C424 | 9827 |  |
| **Mesothelioma** |  | 9050-9055 | 36010 |
| **Kaposi Sarcoma** |  | 9140 | 36020 |
| **Miscellaneous** |  | 9740-9741, 9749-9769, 9950, 9960-9962, 9965-9968, 9970-9971, 9975, 9980, 9982-9987, 9989, 9991-9993 | 37000 |
|  | C760-C768, C809 | excluding 9050-9055, 9140, 9590-9993 |  |
|  | C420-C424 |  |  |
|  | C770-C779 |  |  |
| **Invalid** | Site or histology code not within valid range or site code not found in this table. | | 99999 |
