# Supplementary material for: Trends analysis of cancer incidence, mortality, and survival for the elderly in the United States, 1975–2020
Source: Cancer Med. 2024 Jul 31;13(15):e70062. doi: 10.1002/cam4.70062 (PMC11289898; doi:10.1002/cam4.70062)
Supplement: Supplementary file 1 — Appendix S1. [file CAM4-13-e70062-s001.zip › Supplementary Table 10 Average annual percentage c.docx]

**Supplementary Table 10** Average annual percentage change (AAPC) of all cancers’ incidence by sex, United States, 1975-2020.

| Cohort | Lower Endpoint | Upper Endpoint | AAPC | Lower CI | Upper CI |
| --- | --- | --- | --- | --- | --- |
| Male & Female - 2 Joinpoints | 1975 | 2020 | 0.1315 | -0.0614 | 0.3248 |
| Male - 2 Joinpoints | 1975 | 2020 | -0.1535 | -0.2761 | 0.0086 |
| Female - 2 Joinpoints | 1975 | 2020 | 0.1238 | -0.0331 | 0.2809 |
| Male / Prostate - 2 Joinpoints | 1975 | 2020 | 0.2239 | -0.5335 | 0.9871 |
| Male / Lung and Bronchus - 2 Joinpoints | 1975 | 2020 | -0.9029* | -1.1132 | -0.6922 |
| Male / Colon and Rectum - 2 Joinpoints | 1975 | 2020 | -1.9372* | -2.0369 | -1.7943 |
| Male / Urinary Bladder - 2 Joinpoints | 1975 | 2020 | -0.1374* | -0.2057 | -0.0449 |
| Male / Melanoma of the Skin - 2 Joinpoints | 1975 | 2020 | 3.8160* | 3.5467 | 4.0859 |
| Male / Non-Hodgkin Lymphoma - 2 Joinpoints | 1975 | 2020 | 1.4504* | 1.2058 | 1.6956 |
| Male / Kidney and Renal Pelvis - 1 Joinpoint | 1975 | 2020 | 1.7391* | 1.6248 | 1.9214 |
| Male / Leukemia - 2 Joinpoints | 1975 | 2020 | -0.2405* | -0.3356 | -0.1222 |
| Male / Pancreas - 2 Joinpoints | 1975 | 2020 | -0.0101 | -0.1144 | 0.1392 |
| Male / Oral Cavity and Pharynx - 2 Joinpoints | 1975 | 2020 | -0.7477* | -0.8567 | -0.6172 |
| Male / Stomach - 2 Joinpoints | 1975 | 2020 | -1.6226* | -1.7705 | -1.4144 |
| Male / Liver - 1 Joinpoint | 1975 | 2020 | 2.6366* | 2.5142 | 2.8905 |
| Male / Anus, Anal Canal and Anorectum - 0 Joinpoints | 1975 | 2020 | 1.4008* | 1.1858 | 1.8485 |
| Male / Bones and Joints - 0 Joinpoints | 1975 | 2020 | 0.2401 | -0.1018 | 0.8353 |
| Male / Brain and Other Nervous System - 1 Joinpoint | 1975 | 2020 | 0.4897* | 0.2917 | 0.7995 |
| Male / Endocrine System - 2 Joinpoints | 1975 | 2020 | 2.7230* | 2.4049 | 3.147 |
| Male / Eye and Orbit - 0 Joinpoints | 1975 | 2020 | 0.0066 | -0.2232 | 0.4204 |
| Male / Gallbladder - 1 Joinpoint | 1975 | 2020 | -1.1156* | -1.503 | -0.7016 |
| Male / Hodgkin Lymphoma - 2 Joinpoints | 1975 | 2020 | -1.0302* | -1.2721 | -0.7604 |
| Male / Intrahepatic Bile Duct - 2 Joinpoints | 1975 | 2020 | 5.1939* | 4.6223 | 6.3965 |
| Male / Kaposi Sarcoma - 0 Joinpoints | 1975 | 2020 | -2.0683* | -2.3948 | -1.558 |
| Male / Larynx - 2 Joinpoints | 1975 | 2020 | -1.3231* | -1.4847 | -1.0768 |
| Male / Mesothelioma - 2 Joinpoints | 1975 | 2020 | 0.4664* | 0.0794 | 1.0613 |
| Male / Nose, Nasal Cavity and Middle Ear - 0 Joinpoints | 1975 | 2020 | -0.3032 | -0.5721 | 0.1412 |
| Male / Other Biliary - 2 Joinpoints | 1975 | 2020 | 0.1838 | -0.1072 | 0.4682 |
| Male / Other Digestive Organs - 1 Joinpoint | 1975 | 2020 | 1.1835* | 0.658 | 1.8719 |
| Male / Other Male Genital Organs - 0 Joinpoints | 1975 | 2020 | -0.5498 | -0.9637 | 0.1025 |
| Male / Other Non-Epithelial Skin - 2 Joinpoints | 1975 | 2020 | 4.4498* | 4.0427 | 5.2294 |
| Male / Other Urinary Organs - 2 Joinpoints | 1975 | 2020 | -0.3412 | -0.8529 | 0.3697 |
| Male / Pancreas - 2 Joinpoints | 1975 | 2020 | -0.0064 | -0.1111 | 0.1444 |
| Male / Penis - 1 Joinpoint | 1975 | 2020 | -0.8331* | -1.5399 | -0.0043 |
| Male / Peritoneum, Omentum and Mesentery - 0 Joinpoints | 1975 | 2020 | 0.3098 | -0.5718 | 2.0279 |
| Male / Pleura - 0 Joinpoints | 1975 | 2020 | -1.2732 | -2.067 | 0.1258 |
| Male / Retroperitoneum - 0 Joinpoints | 1975 | 2020 | -0.4257 | -0.7899 | 0.2048 |
| Male / Small Intestine - 1 Joinpoint | 1975 | 2020 | 1.8866* | 1.7062 | 2.1974 |
| Male / Soft Tissue including Heart - 1 Joinpoint | 1975 | 2020 | 1.1833* | 1.009 | 1.4749 |
| Male / Testis - 0 Joinpoints | 1975 | 2020 | -0.5313 | -0.9029 | 0.1202 |
| Male / Trachea, Mediastinum and Other Respiratory Organs - 0 Joinpoints | 1975 | 2020 | -2.1916* | -2.794 | -1.2397 |
| Male / Ureter - 0 Joinpoints | 1975 | 2020 | -0.7014* | -0.9182 | -0.342 |
| Female / Breast - 2 Joinpoints | 1975 | 2020 | 0.7974* | 0.4297 | 1.1664 |
| Female / Lung and Bronchus - 2 Joinpoints | 1975 | 2020 | 2.2890* | 1.939 | 2.6403 |
| Female / Colon and Rectum - 2 Joinpoints | 1975 | 2020 | -1.8640* | -2.0801 | -1.6474 |
| Female / Corpus Uteri - 2 Joinpoints | 1975 | 2020 | -0.0937 | -0.4279 | 0.2416 |
| Female / Non-Hodgkin Lymphoma - 2 Joinpoints | 1975 | 2020 | 1.1314* | 1.0197 | 1.3042 |
| Female / Pancreas - 2 Joinpoints | 1975 | 2020 | 0.3771* | 0.1367 | 0.618 |
| Female / Urinary Bladder - 2 Joinpoints | 1975 | 2020 | -0.3502* | -0.4474 | -0.2198 |
| Female / Melanoma of the Skin - 2 Joinpoints | 1975 | 2020 | 2.7977* | 2.6682 | 3.0608 |
| Female / Leukemia - 2 Joinpoints | 1975 | 2020 | -0.1612* | -0.287 | -0.0246 |
| Female / Ovary - 2 Joinpoints | 1975 | 2020 | -0.8028* | -1.0957 | -0.5089 |
| Female / Kidney and Renal Pelvis - 2 Joinpoints | 1975 | 2020 | 1.6915* | 1.5291 | 2.0179 |
| Female / Stomach - 1 Joinpoint | 1975 | 2020 | -1.6172* | -1.8069 | -1.3159 |
| Female / Anus, Anal Canal and Anorectum - 0 Joinpoints | 1975 | 2020 | 1.8454* | 1.6468 | 2.1618 |
| Female / Bones and Joints - 0 Joinpoints | 1975 | 2020 | -0.3137 | -0.7211 | 0.342 |
| Female / Brain and Other Nervous System - 2 Joinpoints | 1975 | 2020 | 0.5173* | 0.2471 | 0.9593 |
| Female / Cervix Uteri - 1 Joinpoint | 1975 | 2020 | -2.4084* | -2.6402 | -2.1652 |
| Female / Endocrine System - 2 Joinpoints | 1975 | 2020 | 2.5682* | 2.2637 | 2.9775 |
| Female / Eye and Orbit - 0 Joinpoints | 1975 | 2020 | -0.4248 | -0.6981 | 0.0017 |
| Female / Gallbladder - 1 Joinpoint | 1975 | 2020 | -1.5030* | -1.7193 | -1.2499 |
| Female / Hodgkin Lymphoma - 2 Joinpoints | 1975 | 2020 | -0.8415* | -1.3911 | -0.2272 |
| Female / Intrahepatic Bile Duct - 2 Joinpoints | 1975 | 2020 | 5.7114* | 5.2499 | 6.4629 |
| Female / Kaposi Sarcoma - 1 Joinpoint | 1975 | 2020 | -3.9318* | -5.1379 | -2.7369 |
| Female / Larynx - 2 Joinpoints | 1975 | 2020 | -0.4059 | -0.8088 | 0.2301 |
| Female / Mesothelioma - 2 Joinpoints | 1975 | 2020 | 1.9124* | 1.2615 | 3.0669 |
| Female / Nose, Nasal Cavity and Middle Ear - 1 Joinpoint | 1975 | 2020 | -0.1891 | -0.6696 | 0.7079 |
| Female / Other Biliary - 2 Joinpoints | 1975 | 2020 | 0.1711 | -0.108 | 0.46 |
| Female / Other Digestive Organs - 1 Joinpoint | 1975 | 2020 | 1.7366* | 1.2803 | 2.3974 |
| Female / Other Female Genital Organs - 2 Joinpoints | 1975 | 2020 | 2.8401* | 2.471 | 3.5272 |
| Female / Other Non-Epithelial Skin - 2 Joinpoints | 1975 | 2020 | 3.1758* | 2.7181 | 4.1697 |
| Female / Other Urinary Organs - 1 Joinpoint | 1975 | 2020 | 0.2807 | -0.3126 | 0.9944 |
| Female / Peritoneum, Omentum and Mesentery - 2 Joinpoints | 1975 | 2020 | 8.2361* | 7.4547 | 10.2108 |
| Female / Pleura - 0 Joinpoints | 1975 | 2020 | -0.8239 | -1.8888 | 0.9691 |
| Female / Retroperitoneum - 0 Joinpoints | 1975 | 2020 | -0.4589 | -0.7916 | 0.1277 |
| Female / Small Intestine - 1 Joinpoint | 1975 | 2020 | 1.8015* | 1.5652 | 2.1604 |
| Female / Soft Tissue including Heart - 1 Joinpoint | 1975 | 2020 | 1.2607* | 1.007 | 1.7499 |
| Female / Trachea, Mediastinum and Other Respiratory Organs - 0 Joinpoints | 1975 | 2020 | -1.7079* | -2.3565 | -0.6411 |
| Female / Ureter - 0 Joinpoints | 1975 | 2020 | -0.411 | -0.6932 | 0.0648 |
| Female / Uterus, NOS - 1 Joinpoint | 1975 | 2020 | 0.8397* | 0.0961 | 1.7642 |
| Female / Vagina - 0 Joinpoints | 1975 | 2020 | -0.2317 | -0.4306 | 0.1022 |
| Female / Vulva - 2 Joinpoints | 1975 | 2020 | 0.0607 | -0.2201 | 0.3882 |

*Indicate that the Annual Percentage Change (APC) is significantly different from zero at the alpha=0.05 level (P value is not available for the Empirical Quantile method).
