# Supplementary material for: Trends analysis of cancer incidence, mortality, and survival for the elderly in the United States, 1975–2020
Source: Cancer Med. 2024 Jul 31;13(15):e70062. doi: 10.1002/cam4.70062 (PMC11289898; doi:10.1002/cam4.70062)
Supplement: Supplementary file 1 — Appendix S1. [file CAM4-13-e70062-s001.zip › Supplementary Table 11 Average annual percentage c.docx]

**Supplementary Table 11** Average annual percentage change (AAPC) of all cancers’ mortality by sex, United States, 1975-2020.

| Cohort | Lower Endpoint | Upper Endpoint | AAPC | Lower CI | Upper CI |
| --- | --- | --- | --- | --- | --- |
| Male & Female - 2 Joinpoints | 1975 | 2020 | -0.4210* | -0.4644 | -0.3776 |
| Male - 2 Joinpoints | 1975 | 2020 | -0.7432* | -0.7855 | -0.7008 |
| Female - 2 Joinpoints | 1975 | 2020 | -0.1691* | -0.2192 | -0.119 |
| Male / Lung and Bronchus - 2 Joinpoints | 1975 | 2020 | -1.0595* | -1.1648 | -0.9542 |
| Male / Prostate - 2 Joinpoints | 1975 | 2020 | -1.1166* | -1.2368 | -0.9963 |
| Male / Colon and Rectum - 2 Joinpoints | 1975 | 2020 | -2.1195* | -2.2052 | -2.0337 |
| Male / Pancreas - 2 Joinpoints | 1975 | 2020 | -0.0745* | -0.1229 | -0.0195 |
| Male / Leukemia - 2 Joinpoints | 1975 | 2020 | -0.3920* | -0.4409 | -0.3419 |
| Male / Urinary Bladder - 2 Joinpoints | 1975 | 2020 | -0.7611* | -0.8832 | -0.6388 |
| Male / Non-Hodgkin Lymphoma - 2 Joinpoints | 1975 | 2020 | 0.5586* | 0.5101 | 0.6197 |
| Male / Esophagus - 2 Joinpoints | 1975 | 2020 | 0.5206* | 0.3483 | 0.6932 |
| Male / Stomach - 2 Joinpoints | 1975 | 2020 | -2.9457* | -3.013 | -2.8879 |
| Male / Kidney and Renal Pelvis - 2 Joinpoints | 1975 | 2020 | 0.3575* | 0.2958 | 0.4241 |
| Male / Liver - 2 Joinpoints | 1975 | 2020 | 1.7339* | 1.5383 | 1.9299 |
| Male / Myeloma - 1 Joinpoint | 1975 | 2020 | 0.3449* | 0.2836 | 0.42 |
| Male / Small Intestine - 2 Joinpoints | 1975 | 2020 | 0.9396* | 0.7894 | 1.1406 |
| Male / Anus, Anal Canal and Anorectum - 2 Joinpoints | 1975 | 2020 | 4.2804* | 3.4523 | 5.6768 |
| Male / Intrahepatic Bile Duct - 2 Joinpoints | 1975 | 2020 | 6.5444* | 6.2608 | 7.0859 |
| Male / Gallbladder - 2 Joinpoints | 1975 | 2020 | -2.0013* | -2.2168 | -1.8069 |
| Male / Other Biliary - 2 Joinpoints | 1975 | 2020 | -1.6435* | -1.8181 | -1.4389 |
| Male / Pancreas - 2 Joinpoints | 1975 | 2020 | -0.0745* | -0.1229 | -0.0195 |
| Male / Retroperitoneum - 1 Joinpoint | 1975 | 2020 | -2.0127* | -2.3417 | -1.6983 |
| Male / Peritoneum, Omentum and Mesentery - 1 Joinpoint | 1975 | 2020 | 0.4776* | 0.0827 | 0.9289 |
| Male / Other Digestive Organs - 2 Joinpoints | 1975 | 2020 | -0.3617 | -0.7437 | 0.0038 |
| Male / Nose, Nasal Cavity and Middle Ear - 2 Joinpoints | 1975 | 2020 | -2.2971* | -2.7359 | -1.8323 |
| Male / Larynx - 1 Joinpoint | 1975 | 2020 | -1.4902* | -1.5657 | -1.4051 |
| Male / Pleura - 2 Joinpoints | 1975 | 2020 | -0.2614 | -0.5102 | 0.0187 |
| Male / Trachea, Mediastinum and Other Respiratory Organs - 2 Joinpoints | 1975 | 2020 | -3.3624* | -3.7594 | -2.9701 |
| Male / Bones and Joints - 2 Joinpoints | 1975 | 2020 | -1.8170* | -2.1993 | -1.4081 |
| Male / Soft Tissue including Heart - 2 Joinpoints | 1975 | 2020 | 1.3391* | 0.9324 | 1.8125 |
| Male / Non-Melanoma Skin - 2 Joinpoints | 1975 | 2020 | 0.7277* | 0.5557 | 0.8957 |
| Male / Testis - 1 Joinpoint | 1975 | 2020 | -2.0050* | -2.514 | -1.4686 |
| Male / Penis - 1 Joinpoint | 1975 | 2020 | -0.9128* | -1.1468 | -0.6653 |
| Male / Other Male Genital Organs - 1 Joinpoint | 1975 | 2020 | -1.2979* | -1.9018 | -0.5993 |
| Male / Ureter - 0 Joinpoints | 1975 | 2020 | -0.6787* | -0.8929 | -0.4456 |
| Male / Other Urinary Organs - 2 Joinpoints | 1975 | 2020 | 1.2997* | 0.8318 | 1.7999 |
| Male / Eye and Orbit - 1 Joinpoint | 1975 | 2020 | -1.4348* | -1.723 | -1.1352 |
| Male / Brain and Other Nervous System - 2 Joinpoints | 1975 | 2020 | 0.9354* | 0.8556 | 1.0404 |
| Male / Endocrine System - 0 Joinpoints | 1975 | 2020 | 0.9352* | 0.8148 | 1.0904 |
| Male / Hodgkin Lymphoma - 1 Joinpoint | 1975 | 2020 | -2.2864* | -2.5244 | -2.0436 |
| Female / Lung and Bronchus - 2 Joinpoints | 1975 | 2020 | ^1^1.9863* | 1.8207 | 2.1522 |
| Female / Breast - 2 Joinpoints | 1975 | 2020 | -0.5719* | -0.6129 | -0.526 |
| Female / Colon and Rectum - 2 Joinpoints | 1975 | 2020 | -2.1704* | -2.2592 | -2.0816 |
| Female / Pancreas - 2 Joinpoints | 1975 | 2020 | 0.4251* | 0.3322 | 0.518 |
| Female / Ovary - 2 Joinpoints | 1975 | 2020 | -0.4087* | -0.5366 | -0.2807 |
| Female / Non-Hodgkin Lymphoma - 2 Joinpoints | 1975 | 2020 | 0.0910* | 0.0435 | 0.1543 |
| Female / Leukemia - 2 Joinpoints | 1975 | 2020 | -0.3880* | -0.449 | -0.3301 |
| Female / Stomach - 2 Joinpoints | 1975 | 2020 | -2.7876* | -2.8449 | -2.7395 |
| Female / Myeloma - 2 Joinpoints | 1975 | 2020 | 0.2429* | 0.0109 | 0.4754 |
| Female / Urinary Bladder - 2 Joinpoints | 1975 | 2020 | -0.8606* | -0.9879 | -0.7388 |
| Female / Kidney and Renal Pelvis - 2 Joinpoints | 1975 | 2020 | 0.1936* | 0.1251 | 0.2699 |
| Female / Brain and Other Nervous System - 2 Joinpoints | 1975 | 2020 | 0.9562* | 0.8702 | 1.0657 |
| Female / Small Intestine - 2 Joinpoints | 1975 | 2020 | 0.8069* | 0.5843 | 1.1173 |
| Female / Anus, Anal Canal and Anorectum - 2 Joinpoints | 1975 | 2020 | 5.9851* | 5.3077 | 7.0698 |
| Female / Intrahepatic Bile Duct - 2 Joinpoints | 1975 | 2020 | 6.6050* | 6.3579 | 7.0923 |
| Female / Gallbladder - 2 Joinpoints | 1975 | 2020 | -2.3728* | -2.4936 | -2.2464 |
| Female / Other Biliary - 2 Joinpoints | 1975 | 2020 | -1.6229* | -1.784 | -1.4339 |
| Female / Pancreas - 1 Joinpoint | 1975 | 2020 | 0.4400* | 0.398 | 0.4964 |
| Female / Retroperitoneum - 1 Joinpoint | 1975 | 2020 | -1.9309* | -2.3899 | -1.5368 |
| Female / Peritoneum, Omentum and Mesentery - 2 Joinpoints | 1975 | 2020 | 2.5208* | 2.1739 | 2.9607 |
| Female / Other Digestive Organs - 2 Joinpoints | 1975 | 2020 | -0.5362* | -0.8687 | -0.2378 |
| Female / Nose, Nasal Cavity and Middle Ear - 1 Joinpoint | 1975 | 2020 | -2.0055* | -2.3529 | -1.5291 |
| Female / Larynx - 2 Joinpoints | 1975 | 2020 | 0.1157 | -0.1101 | 0.4283 |
| Female / Pleura - 2 Joinpoints | 1975 | 2020 | -1.0327* | -1.4644 | -0.5203 |
| Female / Trachea, Mediastinum and Other Respiratory Organs - 1 Joinpoint | 1975 | 2020 | -1.8438* | -2.4449 | -1.3316 |
| Female / Bones and Joints - 2 Joinpoints | 1975 | 2020 | -1.6176* | -1.9152 | -1.2941 |
| Female / Soft Tissue including Heart - 2 Joinpoints | 1975 | 2020 | 0.6951* | 0.5739 | 0.8662 |
| Female / Non-Melanoma Skin - 2 Joinpoints | 1975 | 2020 | -0.3377* | -0.5548 | -0.0822 |
| Female / Non-Melanoma Skin - 2 Joinpoints | 1975 | 2020 | -0.3377* | -0.5548 | -0.0822 |
| Female / Cervix Uteri - 2 Joinpoints | 1975 | 2020 | -2.6180* | -2.7257 | -2.5092 |
| Female / Uterus, NOS - 2 Joinpoints | 1975 | 2020 | -0.8328* | -0.9833 | -0.7039 |
| Female / Vagina - 0 Joinpoints | 1975 | 2020 | -1.2873* | -1.4243 | -1.1439 |
| Female / Vulva - 2 Joinpoints | 1975 | 2020 | 0.1549* | 0.0049 | 0.3129 |
| Female / Other Female Genital Organs - 2 Joinpoints | 1975 | 2020 | 2.7939* | 2.483 | 3.4368 |
| Female / Ureter - 0 Joinpoints | 1975 | 2020 | -0.2909* | -0.4834 | -0.0706 |
| Female / Other Urinary Organs - 2 Joinpoints | 1975 | 2020 | -0.1214 | -0.4541 | 0.1799 |
| Female / Eye and Orbit - 1 Joinpoint | 1975 | 2020 | -1.5053* | -1.8188 | -1.2033 |
| Female / Brain and Other Nervous System - 2 Joinpoints | 1975 | 2020 | 0.9562* | 0.8702 | 1.0657 |
| Female / Endocrine System - 1 Joinpoint | 1975 | 2020 | -0.2283* | -0.3597 | -0.0941 |
| Female / Hodgkin Lymphoma - 1 Joinpoint | 1975 | 2020 | -2.4391* | -2.668 | -2.2177 |

*Indicate that the Annual Percentage Change (APC) is significantly different from zero at the alpha=0.05 level (P value is not available for the Empirical Quantile method).
