# Supplementary material for: Trends analysis of cancer incidence, mortality, and survival for the elderly in the United States, 1975–2020
Source: Cancer Med. 2024 Jul 31;13(15):e70062. doi: 10.1002/cam4.70062 (PMC11289898; doi:10.1002/cam4.70062)
Supplement: Supplementary file 1 — Appendix S1. [file CAM4-13-e70062-s001.zip › Supplementary Table 12 Annual percentage change.docx]

**Supplementary Table 12** Annual percentage change (APC) of all cancers’ incidence by sex, United States, 1975-2020.

| Cohort | Segment | Lower Endpoint | Upper Endpoint | APC | Lower CI | Upper CI |
| --- | --- | --- | --- | --- | --- | --- |
| Male & Female - 2 Joinpoints | 1 | 1975 | 1991 | 1.6174* | 1.1747 | 2.0619 |
| Male & Female - 2 Joinpoints | 2 | 1991 | 2008 | -0.2091 | -0.4768 | 0.0594 |
| Male & Female - 2 Joinpoints | 3 | 2008 | 2020 | -1.3408* | -1.6091 | -1.0719 |
| Male - 2 Joinpoints | 1 | 1975 | 1991 | 1.6675* | 1.1403 | 2.6838 |
| Male - 2 Joinpoints | 2 | 1991 | 2007 | -0.6313* | -1.0594 | -0.0319 |
| Male - 2 Joinpoints | 3 | 2007 | 2020 | -1.7719* | -2.3873 | -1.482 |
| Female - 2 Joinpoints | 1 | 1975 | 1996 | 1.1193* | 0.933 | 1.306 |
| Female - 2 Joinpoints | 2 | 1996 | 2018 | -0.2870* | -0.3792 | -0.1947 |
| Female - 2 Joinpoints | 3 | 2018 | 2020 | -5.5803* | -8.2934 | -2.787 |
| Male / Prostate - 2 Joinpoints | 1 | 1975 | 1992 | 5.1326* | 3.5581 | 6.7309 |
| Male / Prostate - 2 Joinpoints | 2 | 1992 | 2015 | -3.2434* | -3.8002 | -2.6834 |
| Male / Prostate - 2 Joinpoints | 3 | 2015 | 2020 | 0.1627 | -3.8076 | 4.297 |
| Male / Lung and Bronchus - 2 Joinpoints | 1 | 1975 | 2000 | 0.3024* | 0.0446 | 0.5608 |
| Male / Lung and Bronchus - 2 Joinpoints | 2 | 2000 | 2009 | -1.1785* | -1.8316 | -0.5211 |
| Male / Lung and Bronchus - 2 Joinpoints | 3 | 2009 | 2020 | -3.3687* | -3.7657 | -2.9702 |
| Male / Colon and Rectum - 2 Joinpoints | 1 | 1975 | 1985 | 1.2620* | 0.4773 | 2.5662 |
| Male / Colon and Rectum - 2 Joinpoints | 2 | 1985 | 2002 | -1.5601* | -1.8695 | -1.2148 |
| Male / Colon and Rectum - 2 Joinpoints | 3 | 2002 | 2020 | -4.0193* | -4.2114 | -3.8483 |
| Male / Urinary Bladder - 2 Joinpoints | 1 | 1975 | 2007 | 0.5857* | 0.4997 | 0.7239 |
| Male / Urinary Bladder - 2 Joinpoints | 2 | 2007 | 2017 | -1.2957* | -1.6097 | -0.7784 |
| Male / Urinary Bladder - 2 Joinpoints | 3 | 2017 | 2020 | -3.8683* | -5.9222 | -2.4287 |
| Male / Melanoma of the Skin - 2 Joinpoints | 1 | 1975 | 2007 | 5.1623* | 4.9353 | 5.3897 |
| Male / Melanoma of the Skin - 2 Joinpoints | 2 | 2007 | 2018 | 2.1937* | 1.7956 | 2.5934 |
| Male / Melanoma of the Skin - 2 Joinpoints | 3 | 2018 | 2020 | -7.8809* | -11.896 | -3.6828 |
| Male / Non-Hodgkin Lymphoma - 2 Joinpoints | 1 | 1975 | 1994 | 3.2592* | 2.7614 | 3.7593 |
| Male / Non-Hodgkin Lymphoma - 2 Joinpoints | 2 | 1994 | 2012 | 0.8998* | 0.6326 | 1.1676 |
| Male / Non-Hodgkin Lymphoma - 2 Joinpoints | 3 | 2012 | 2020 | -1.5208* | -2.0539 | -0.9848 |
| Male / Kidney and Renal Pelvis - 1 Joinpoint | 1 | 1975 | 2009 | 2.3599* | 2.1913 | 2.6489 |
| Male / Kidney and Renal Pelvis - 1 Joinpoint | 2 | 2009 | 2020 | -0.156 | -0.7181 | 0.3147 |
| Male / Leukemia - 2 Joinpoints | 1 | 1975 | 2007 | -0.1434 | -0.344 | 0.0423 |
| Male / Leukemia - 2 Joinpoints | 2 | 2007 | 2012 | 2.4849* | 1.0864 | 4.7435 |
| Male / Leukemia - 2 Joinpoints | 3 | 2012 | 2020 | -2.2881* | -3.0166 | -1.7264 |
| Male / Pancreas - 2 Joinpoints | 1 | 1975 | 1996 | -0.6336* | -1.1397 | -0.2416 |
| Male / Pancreas - 2 Joinpoints | 2 | 1996 | 2018 | 0.8590* | 0.7179 | 1.2145 |
| Male / Pancreas - 2 Joinpoints | 3 | 2018 | 2020 | -2.9182 | -4.3201 | 0.4125 |
| Male / Oral Cavity and Pharynx - 2 Joinpoints | 1 | 1975 | 2005 | -1.4963* | -1.671 | -1.306 |
| Male / Oral Cavity and Pharynx - 2 Joinpoints | 2 | 2005 | 2018 | 1.4652* | 1.1482 | 2.0671 |
| Male / Oral Cavity and Pharynx - 2 Joinpoints | 3 | 2018 | 2020 | -3.6600* | -6.1462 | -0.0692 |
| Male / Stomach - 2 Joinpoints | 1 | 1975 | 1980 | 0.3785 | -1.4868 | 5.8767 |
| Male / Stomach - 2 Joinpoints | 2 | 1980 | 2014 | -1.6655* | -1.8575 | -1.5541 |
| Male / Stomach - 2 Joinpoints | 3 | 2014 | 2020 | -3.0203* | -4.5686 | -2.2663 |
| Male / Liver - 1 Joinpoint | 1 | 1975 | 2018 | 3.0195* | 2.9021 | 3.274 |
| Male / Liver - 1 Joinpoint | 2 | 2018 | 2020 | -5.2599* | -8.0052 | -0.4025 |
| Male / Anus, Anal Canal and Anorectum - 0 Joinpoints | 1 | 1975 | 2020 | 1.4008* | 1.1858 | 1.8485 |
| Male / Bones and Joints - 0 Joinpoints | 1 | 1975 | 2020 | 0.2401 | -0.1018 | 0.8353 |
| Male / Brain and Other Nervous System - 1 Joinpoint | 1 | 1975 | 1992 | 1.9181* | 0.9966 | 3.5173 |
| Male / Brain and Other Nervous System - 1 Joinpoint | 2 | 1992 | 2020 | -0.3677* | -0.6228 | -0.1213 |
| Male / Endocrine System - 2 Joinpoints | 1 | 1975 | 1998 | 2.0790* | 0.6047 | 3.2316 |
| Male / Endocrine System - 2 Joinpoints | 2 | 1998 | 2009 | 6.9486* | 5.705 | 10.0988 |
| Male / Endocrine System - 2 Joinpoints | 3 | 2009 | 2020 | -0.0297 | -0.8481 | 0.6952 |
| Male / Eye and Orbit - 0 Joinpoints | 1 | 1975 | 2020 | 0.0066 | -0.2232 | 0.4204 |
| Male / Gallbladder - 1 Joinpoint | 1 | 1975 | 1996 | -2.0827* | -7.0248 | -1.0594 |
| Male / Gallbladder - 1 Joinpoint | 2 | 1996 | 2020 | -0.2615 | -0.7354 | 1.4373 |
| Male / Hodgkin Lymphoma - 2 Joinpoints | 1 | 1975 | 2001 | -1.4382* | -2.3194 | -0.8738 |
| Male / Hodgkin Lymphoma - 2 Joinpoints | 2 | 2001 | 2005 | 6.9597* | 2.3985 | 12.0418 |
| Male / Hodgkin Lymphoma - 2 Joinpoints | 3 | 2005 | 2020 | -2.3613* | -3.0927 | -1.794 |
| Male / Intrahepatic Bile Duct - 2 Joinpoints | 1 | 1975 | 1997 | 9.6402* | 7.7264 | 12.9479 |
| Male / Intrahepatic Bile Duct - 2 Joinpoints | 2 | 1997 | 2004 | -11.9114* | -21.7827 | -6.7659 |
| Male / Intrahepatic Bile Duct - 2 Joinpoints | 3 | 2004 | 2020 | 7.3959* | 6.4466 | 8.6778 |
| Male / Kaposi Sarcoma - 0 Joinpoints | 1 | 1975 | 2020 | -2.0683* | -2.3948 | -1.558 |
| Male / Larynx - 2 Joinpoints | 1 | 1975 | 1986 | 0.703 | -0.3235 | 3.337 |
| Male / Larynx - 2 Joinpoints | 2 | 1986 | 2011 | -1.3962* | -1.6735 | -1.1172 |
| Male / Larynx - 2 Joinpoints | 3 | 2011 | 2020 | -3.5459* | -4.543 | -2.9041 |
| Male / Mesothelioma - 2 Joinpoints | 1 | 1975 | 1992 | 5.3539* | 3.797 | 7.8796 |
| Male / Mesothelioma - 2 Joinpoints | 2 | 1992 | 2013 | -1.0281* | -1.6131 | -0.2361 |
| Male / Mesothelioma - 2 Joinpoints | 3 | 2013 | 2020 | -6.3630* | -9.5584 | -4.4365 |
| Male / Nose, Nasal Cavity and Middle Ear - 0 Joinpoints | 1 | 1975 | 2020 | -0.3032 | -0.5721 | 0.1412 |
| Male / Other Biliary - 2 Joinpoints | 1 | 1975 | 1994 | -0.3221 | -2.4674 | 0.6485 |
| Male / Other Biliary - 2 Joinpoints | 2 | 1994 | 2011 | 1.9689* | 1.4057 | 4.0494 |
| Male / Other Biliary - 2 Joinpoints | 3 | 2011 | 2020 | -2.0621* | -3.2865 | -1.1476 |
| Male / Other Digestive Organs - 1 Joinpoint | 1 | 1975 | 1996 | -1.2298 | -5.599 | 0.6561 |
| Male / Other Digestive Organs - 1 Joinpoint | 2 | 1996 | 2020 | 3.3436* | 2.7723 | 4.3896 |
| Male / Other Male Genital Organs - 0 Joinpoints | 1 | 1975 | 2020 | -0.5498 | -0.9637 | 0.1025 |
| Male / Other Non-Epithelial Skin - 2 Joinpoints | 1 | 1975 | 1996 | 8.1360* | 6.7164 | 10.7346 |
| Male / Other Non-Epithelial Skin - 2 Joinpoints | 2 | 1996 | 2014 | 2.5222* | 1.933 | 3.3512 |
| Male / Other Non-Epithelial Skin - 2 Joinpoints | 3 | 2014 | 2020 | -2.1735* | -5.0451 | -0.4718 |
| Male / Other Urinary Organs - 2 Joinpoints | 1 | 1975 | 2004 | -1.5458* | -3.251 | -0.2544 |
| Male / Other Urinary Organs - 2 Joinpoints | 2 | 2004 | 2016 | 4.6055* | 2.5238 | 15.7385 |
| Male / Other Urinary Organs - 2 Joinpoints | 3 | 2016 | 2020 | -5.8782 | -16.5903 | 1.0559 |
| Male / Pancreas - 2 Joinpoints | 1 | 1975 | 1996 | -0.6184* | -1.1261 | -0.2224 |
| Male / Pancreas - 2 Joinpoints | 2 | 1996 | 2018 | 0.8512* | 0.7087 | 1.2256 |
| Male / Pancreas - 2 Joinpoints | 3 | 2018 | 2020 | -2.9115 | -4.3273 | 0.4092 |
| Male / Penis - 1 Joinpoint | 1 | 1975 | 1984 | -4.6331* | -24.68 | -0.1498 |
| Male / Penis - 1 Joinpoint | 2 | 1984 | 2020 | 0.1403 | -0.3634 | 1.8858 |
| Male / Peritoneum, Omentum and Mesentery - 0 Joinpoints | 1 | 1975 | 2020 | 0.3098 | -0.5718 | 2.0279 |
| Male / Pleura - 0 Joinpoints | 1 | 1975 | 2020 | -1.2732 | -2.067 | 0.1258 |
| Male / Retroperitoneum - 0 Joinpoints | 1 | 1975 | 2020 | -0.4257 | -0.7899 | 0.2048 |
| Male / Small Intestine - 1 Joinpoint | 1 | 1975 | 2012 | 2.3613* | 2.1516 | 2.8166 |
| Male / Small Intestine - 1 Joinpoint | 2 | 2012 | 2020 | -0.2802 | -2.1538 | 0.7976 |
| Male / Soft Tissue including Heart - 1 Joinpoint | 1 | 1975 | 2016 | 1.6033* | 1.4431 | 1.9278 |
| Male / Soft Tissue including Heart - 1 Joinpoint | 2 | 2016 | 2020 | -3.0228* | -8.0998 | -0.256 |
| Male / Testis - 0 Joinpoints | 1 | 1975 | 2020 | -0.5313 | -0.9029 | 0.1202 |
| Male / Trachea, Mediastinum and Other Respiratory Organs - 0 Joinpoints | 1 | 1975 | 2020 | -2.1916* | -2.794 | -1.2397 |
| Male / Ureter - 0 Joinpoints | 1 | 1975 | 2020 | -0.7014* | -0.9182 | -0.342 |
| Female / Breast - 2 Joinpoints | 1 | 1975 | 1990 | 3.0583* | 2.1553 | 3.9693 |
| Female / Breast - 2 Joinpoints | 2 | 1990 | 2005 | -0.7518* | -1.3613 | -0.1385 |
| Female / Breast - 2 Joinpoints | 3 | 2005 | 2020 | 0.1249 | -0.2329 | 0.484 |
| Female / Lung and Bronchus - 2 Joinpoints | 1 | 1975 | 1990 | 6.6032* | 5.6367 | 7.5786 |
| Female / Lung and Bronchus - 2 Joinpoints | 2 | 1990 | 2007 | 1.8905* | 1.479 | 2.3036 |
| Female / Lung and Bronchus - 2 Joinpoints | 3 | 2007 | 2020 | -1.9731* | -2.3086 | -1.6366 |
| Female / Colon and Rectum - 2 Joinpoints | 1 | 1975 | 1983 | 0.8023 | -0.2877 | 1.9042 |
| Female / Colon and Rectum - 2 Joinpoints | 2 | 1983 | 2002 | -1.2224* | -1.4528 | -0.9915 |
| Female / Colon and Rectum - 2 Joinpoints | 3 | 2002 | 2020 | -3.6909* | -3.8568 | -3.5247 |
| Female / Corpus Uteri - 2 Joinpoints | 1 | 1975 | 1997 | 0.0613 | -0.2679 | 0.3916 |
| Female / Corpus Uteri - 2 Joinpoints | 2 | 1997 | 2003 | -3.8532* | -5.9455 | -1.7145 |
| Female / Corpus Uteri - 2 Joinpoints | 3 | 2003 | 2020 | 1.0650* | 0.8293 | 1.3013 |
| Female / Non-Hodgkin Lymphoma - 2 Joinpoints | 1 | 1975 | 1991 | 3.1217* | 2.6013 | 3.9528 |
| Female / Non-Hodgkin Lymphoma - 2 Joinpoints | 2 | 1991 | 2008 | 0.9018* | 0.5714 | 1.2681 |
| Female / Non-Hodgkin Lymphoma - 2 Joinpoints | 3 | 2008 | 2020 | -1.1451* | -1.5126 | -0.8564 |
| Female / Pancreas - 2 Joinpoints | 1 | 1975 | 2001 | 0.2750* | 0.0277 | 0.5229 |
| Female / Pancreas - 2 Joinpoints | 2 | 2001 | 2008 | 1.3512* | 0.1949 | 2.5208 |
| Female / Pancreas - 2 Joinpoints | 3 | 2008 | 2020 | 0.0334 | -0.3238 | 0.392 |
| Female / Urinary Bladder - 2 Joinpoints | 1 | 1975 | 2004 | 0.4546* | 0.3211 | 0.6599 |
| Female / Urinary Bladder - 2 Joinpoints | 2 | 2004 | 2018 | -1.1307* | -1.3932 | -0.7838 |
| Female / Urinary Bladder - 2 Joinpoints | 3 | 2018 | 2020 | -6.3027* | -8.401 | -2.9958 |
| Female / Melanoma of the Skin - 2 Joinpoints | 1 | 1975 | 2007 | 3.4500* | 3.2545 | 4.5054 |
| Female / Melanoma of the Skin - 2 Joinpoints | 2 | 2007 | 2018 | 2.5513* | 1.614 | 3.0662 |
| Female / Melanoma of the Skin - 2 Joinpoints | 3 | 2018 | 2020 | -5.8627* | -8.1219 | -2.1014 |
| Female / Leukemia - 2 Joinpoints | 1 | 1975 | 1999 | -0.2889 | -0.7983 | 0.0467 |
| Female / Leukemia - 2 Joinpoints | 2 | 1999 | 2013 | 1.0499* | 0.7066 | 2.1723 |
| Female / Leukemia - 2 Joinpoints | 3 | 2013 | 2020 | -2.1110* | -3.1374 | -1.324 |
| Female / Ovary - 2 Joinpoints | 1 | 1975 | 1990 | 1.4116* | 0.706 | 2.1222 |
| Female / Ovary - 2 Joinpoints | 2 | 1990 | 2009 | -0.9924* | -1.3362 | -0.6474 |
| Female / Ovary - 2 Joinpoints | 3 | 2009 | 2020 | -3.4260* | -3.9485 | -2.9007 |
| Female / Kidney and Renal Pelvis - 2 Joinpoints | 1 | 1975 | 2007 | 2.8085* | 2.5625 | 3.3368 |
| Female / Kidney and Renal Pelvis - 2 Joinpoints | 2 | 2007 | 2018 | 0.1258 | -0.4582 | 2.6853 |
| Female / Kidney and Renal Pelvis - 2 Joinpoints | 3 | 2018 | 2020 | -7.0103* | -10.121 | -0.8559 |
| Female / Stomach - 1 Joinpoint | 1 | 1975 | 2016 | -1.3981* | -1.4949 | -0.4509 |
| Female / Stomach - 1 Joinpoint | 2 | 2016 | 2020 | -3.8355* | -8.3812 | -1.5799 |
| Female / Anus, Anal Canal and Anorectum - 0 Joinpoints | 1 | 1975 | 2020 | 1.8454* | 1.6468 | 2.1618 |
| Female / Bones and Joints - 0 Joinpoints | 1 | 1975 | 2020 | -0.3137 | -0.7211 | 0.342 |
| Female / Brain and Other Nervous System - 2 Joinpoints | 1 | 1975 | 1986 | 3.3742* | 1.3715 | 10.5919 |
| Female / Brain and Other Nervous System - 2 Joinpoints | 2 | 1986 | 2009 | -0.0046 | -0.3999 | 2.5077 |
| Female / Brain and Other Nervous System - 2 Joinpoints | 3 | 2009 | 2020 | -1.1909* | -4.1797 | -0.4929 |
| Female / Cervix Uteri - 1 Joinpoint | 1 | 1975 | 1982 | -4.8790* | -11.4223 | -2.8407 |
| Female / Cervix Uteri - 1 Joinpoint | 2 | 1982 | 2020 | -1.9463* | -2.0778 | -1.6901 |
| Female / Endocrine System - 2 Joinpoints | 1 | 1975 | 1996 | 1.442 | -0.0729 | 2.635 |
| Female / Endocrine System - 2 Joinpoints | 2 | 1996 | 2010 | 7.5624* | 6.6097 | 9.0073 |
| Female / Endocrine System - 2 Joinpoints | 3 | 2010 | 2020 | -1.7858* | -2.6612 | -0.9469 |
| Female / Eye and Orbit - 0 Joinpoints | 1 | 1975 | 2020 | -0.4248 | -0.6981 | 0.0017 |
| Female / Gallbladder - 1 Joinpoint | 1 | 1975 | 1997 | -2.6462* | -3.8355 | -1.9717 |
| Female / Gallbladder - 1 Joinpoint | 2 | 1997 | 2020 | -0.3969 | -0.7726 | 0.1497 |
| Female / Hodgkin Lymphoma - 2 Joinpoints | 1 | 1975 | 2001 | -1.0128 | -6.166 | 0.2825 |
| Female / Hodgkin Lymphoma - 2 Joinpoints | 2 | 2001 | 2004 | 9.04 | -7.3966 | 14.9137 |
| Female / Hodgkin Lymphoma - 2 Joinpoints | 3 | 2004 | 2020 | -2.3182 | -4.1316 | 2.2743 |
| Female / Intrahepatic Bile Duct - 2 Joinpoints | 1 | 1975 | 1997 | 9.8936* | 8.4775 | 12.0493 |
| Female / Intrahepatic Bile Duct - 2 Joinpoints | 2 | 1997 | 2003 | -10.7651* | -19.0222 | -5.996 |
| Female / Intrahepatic Bile Duct - 2 Joinpoints | 3 | 2003 | 2020 | 6.7302* | 6.0958 | 7.5315 |
| Female / Kaposi Sarcoma - 1 Joinpoint | 1 | 1975 | 2015 | -1.8377* | -2.3602 | -0.8072 |
| Female / Kaposi Sarcoma - 1 Joinpoint | 2 | 2015 | 2020 | -19.1534* | -40.2386 | -8.2255 |
| Female / Larynx - 2 Joinpoints | 1 | 1975 | 1991 | 3.7915* | 1.6786 | 7.026 |
| Female / Larynx - 2 Joinpoints | 2 | 1991 | 2015 | -1.9321 | -2.3516 | 4.5813 |
| Female / Larynx - 2 Joinpoints | 3 | 2015 | 2020 | -6.0157* | -13.3488 | -2.415 |
| Female / Mesothelioma - 2 Joinpoints | 1 | 1975 | 1983 | 12.5973* | 6.0652 | 31.3605 |
| Female / Mesothelioma - 2 Joinpoints | 2 | 1983 | 2018 | 0.4430* | 0.1247 | 1.1671 |
| Female / Mesothelioma - 2 Joinpoints | 3 | 2018 | 2020 | -11.8138* | -18.257 | -0.4465 |
| Female / Nose, Nasal Cavity and Middle Ear - 1 Joinpoint | 1 | 1975 | 2008 | 0.3629 | -0.1263 | 6.8872 |
| Female / Nose, Nasal Cavity and Middle Ear - 1 Joinpoint | 2 | 2008 | 2020 | -1.6912* | -8.4536 | -0.2729 |
| Female / Other Biliary - 2 Joinpoints | 1 | 1975 | 1996 | -0.6447 | -2.371 | 0.2238 |
| Female / Other Biliary - 2 Joinpoints | 2 | 1996 | 2005 | 3.8016* | 2.0021 | 10.5133 |
| Female / Other Biliary - 2 Joinpoints | 3 | 2005 | 2020 | -0.8169* | -1.4938 | -0.2827 |
| Female / Other Digestive Organs - 1 Joinpoint | 1 | 1975 | 2004 | 0.4719 | -2.2226 | 1.5587 |
| Female / Other Digestive Organs - 1 Joinpoint | 2 | 2004 | 2020 | 4.0696* | 3.1202 | 6.7855 |
| Female / Other Female Genital Organs - 2 Joinpoints | 1 | 1975 | 2005 | -0.3005 | -1.2433 | 0.9134 |
| Female / Other Female Genital Organs - 2 Joinpoints | 2 | 2005 | 2018 | 11.0768* | 0.1208 | 16.1421 |
| Female / Other Female Genital Organs - 2 Joinpoints | 3 | 2018 | 2020 | -0.7544 | -7.3109 | 9.7015 |
| Female / Other Non-Epithelial Skin - 2 Joinpoints | 1 | 1975 | 1996 | 5.8612* | 4.452 | 12.6313 |
| Female / Other Non-Epithelial Skin - 2 Joinpoints | 2 | 1996 | 2013 | 2.0880* | 1.0404 | 3.7254 |
| Female / Other Non-Epithelial Skin - 2 Joinpoints | 3 | 2013 | 2020 | -1.9876* | -6.8472 | -0.0017 |
| Female / Other Urinary Organs - 1 Joinpoint | 1 | 1975 | 2001 | -2.1698* | -4.8643 | -0.7321 |
| Female / Other Urinary Organs - 1 Joinpoint | 2 | 2001 | 2020 | 3.7338* | 2.7384 | 5.5208 |
| Female / Peritoneum, Omentum and Mesentery - 2 Joinpoints | 1 | 1975 | 1997 | 17.9833* | 15.5625 | 24.0029 |
| Female / Peritoneum, Omentum and Mesentery - 2 Joinpoints | 2 | 1997 | 2007 | 4.3902* | 2.0941 | 7.6388 |
| Female / Peritoneum, Omentum and Mesentery - 2 Joinpoints | 3 | 2007 | 2020 | -3.8199* | -5.0187 | -2.8928 |
| Female / Pancreas - 0 Joinpoints | 1 | 1975 | 2020 | 0.4609* | 0.3838 | 0.5764 |
| Female / Pleura - 0 Joinpoints | 1 | 1975 | 2020 | -0.8239 | -1.8888 | 0.9691 |
| Female / Retroperitoneum - 0 Joinpoints | 1 | 1975 | 2020 | -0.4589 | -0.7916 | 0.1277 |
| Female / Small Intestine - 1 Joinpoint | 1 | 1975 | 2016 | 2.3143* | 2.1117 | 2.7298 |
| Female / Small Intestine - 1 Joinpoint | 2 | 2016 | 2020 | -3.3079 | -9.2368 | 0.0613 |
| Female / Soft Tissue including Heart - 1 Joinpoint | 1 | 1975 | 2008 | 1.6580* | 1.3564 | 3.4099 |
| Female / Soft Tissue including Heart - 1 Joinpoint | 2 | 2008 | 2020 | 0.1762 | -2.472 | 0.9826 |
| Female / Trachea, Mediastinum and Other Respiratory Organs - 0 Joinpoints | 1 | 1975 | 2020 | -1.7079* | -2.3565 | -0.6411 |
| Female / Ureter - 0 Joinpoints | 1 | 1975 | 2020 | -0.411 | -0.6932 | 0.0648 |
| Female / Uterus, NOS - 1 Joinpoint | 1 | 1975 | 1986 | -4.5554 | -21.936 | 0.4003 |
| Female / Uterus, NOS - 1 Joinpoint | 2 | 1986 | 2020 | 2.6497* | 2.2193 | 3.5529 |
| Female / Vagina - 0 Joinpoints | 1 | 1975 | 2020 | -0.2317 | -0.4306 | 0.1022 |
| Female / Vulva - 2 Joinpoints | 1 | 1975 | 2004 | -0.1523 | -1.1288 | 0.4119 |
| Female / Vulva - 2 Joinpoints | 2 | 2004 | 2017 | 1.5127 | -0.1451 | 5.7728 |
| Female / Vulva - 2 Joinpoints | 3 | 2017 | 2020 | -4.0386 | -9.2488 | 0.7118 |

*Indicate that the Annual Percentage Change (APC) is significantly different from zero at the alpha=0.05 level (P value is not available for the Empirical Quantile method).
