# Supplementary material for: Trends analysis of cancer incidence, mortality, and survival for the elderly in the United States, 1975–2020
Source: Cancer Med. 2024 Jul 31;13(15):e70062. doi: 10.1002/cam4.70062 (PMC11289898; doi:10.1002/cam4.70062)
Supplement: Supplementary file 1 — Appendix S1. [file CAM4-13-e70062-s001.zip › Supplementary Table 13 Annual percentage change.docx]

**Supplementary Table 13** Annual percentage change (APC) of all cancers’ mortality by sex, United States, 1975-2020.

| Cohort | Segment | Lower Endpoint | Upper Endpoint | APC | Lower CI | Upper CI |
| --- | --- | --- | --- | --- | --- | --- |
| Male & Female - 2 Joinpoints | 1 | 1975 | 1992 | 0.8554* | 0.7891 | 0.9218 |
| Male & Female - 2 Joinpoints | 2 | 1992 | 2002 | -0.5376* | -0.6807 | -0.3943 |
| Male & Female - 2 Joinpoints | 3 | 2002 | 2020 | -1.5477* | -1.596 | -1.4993 |
| Male - 2 Joinpoints | 1 | 1975 | 1992 | 0.7355* | 0.6709 | 0.8001 |
| Male - 2 Joinpoints | 2 | 1992 | 2002 | -1.2109* | -1.3509 | -1.0706 |
| Male - 2 Joinpoints | 3 | 2002 | 2020 | -1.8626* | -1.9093 | -1.8159 |
| Female - 2 Joinpoints | 1 | 1975 | 1993 | 1.0796* | 1.009 | 1.1503 |
| Female - 2 Joinpoints | 2 | 1993 | 2003 | -0.2268* | -0.3919 | -0.0616 |
| Female - 2 Joinpoints | 3 | 2003 | 2020 | -1.4410* | -1.5022 | -1.3797 |
| Male / Lung and Bronchus - 2 Joinpoints | 1 | 1975 | 1991 | 1.6886* | 1.5013 | 1.8762 |
| Male / Lung and Bronchus - 2 Joinpoints | 2 | 1991 | 2009 | -1.4565* | -1.5949 | -1.3179 |
| Male / Lung and Bronchus - 2 Joinpoints | 3 | 2009 | 2020 | -4.2972* | -4.5632 | -4.0305 |
| Male / Prostate - 2 Joinpoints | 1 | 1975 | 1993 | 1.5081* | 1.3468 | 1.6697 |
| Male / Prostate - 2 Joinpoints | 2 | 1993 | 2013 | -3.5756* | -3.7003 | -3.4506 |
| Male / Prostate - 2 Joinpoints | 3 | 2013 | 2020 | -0.6626* | -1.2465 | -0.0752 |
| Male / Colon and Rectum - 2 Joinpoints | 1 | 1975 | 1985 | 0.029 | -0.2494 | 0.3082 |
| Male / Colon and Rectum - 2 Joinpoints | 2 | 1985 | 2000 | -1.9107* | -2.0623 | -1.7588 |
| Male / Colon and Rectum - 2 Joinpoints | 3 | 2000 | 2020 | -3.3310* | -3.4205 | -3.2414 |
| Male / Pancreas - 2 Joinpoints | 1 | 1975 | 1983 | -0.8661* | -2.2273 | -0.412 |
| Male / Pancreas - 2 Joinpoints | 2 | 1983 | 2000 | -0.2127 | -0.3628 | 0.2607 |
| Male / Pancreas - 2 Joinpoints | 3 | 2000 | 2020 | 0.3618* | 0.2768 | 0.5026 |
| Male / Leukemia - 2 Joinpoints | 1 | 1975 | 2000 | 0.2184* | 0.1339 | 0.3403 |
| Male / Leukemia - 2 Joinpoints | 2 | 2000 | 2012 | -0.5290* | -0.8347 | -0.2619 |
| Male / Leukemia - 2 Joinpoints | 3 | 2012 | 2020 | -2.0737* | -2.5708 | -1.737 |
| Male / Urinary Bladder - 2 Joinpoints | 1 | 1975 | 1989 | -1.6515* | -1.877 | -1.4255 |
| Male / Urinary Bladder - 2 Joinpoints | 2 | 1989 | 2016 | -0.0815* | -0.1563 | -0.0066 |
| Male / Urinary Bladder - 2 Joinpoints | 3 | 2016 | 2020 | -2.1918* | -3.2333 | -1.1391 |
| Male / Non-Hodgkin Lymphoma - 2 Joinpoints | 1 | 1975 | 1990 | 3.3364* | 3.1206 | 3.6349 |
| Male / Non-Hodgkin Lymphoma - 2 Joinpoints | 2 | 1990 | 1998 | 1.9371* | 1.1633 | 2.3878 |
| Male / Non-Hodgkin Lymphoma - 2 Joinpoints | 3 | 1998 | 2020 | -1.7799* | -1.8673 | -1.6962 |
| Male / Esophagus - 2 Joinpoints | 1 | 1975 | 2001 | 1.4378* | 1.3303 | 1.5454 |
| Male / Esophagus - 2 Joinpoints | 2 | 2001 | 2006 | 0.221 | -1.1706 | 1.6322 |
| Male / Esophagus - 2 Joinpoints | 3 | 2006 | 2020 | -1.0555* | -1.2436 | -0.8669 |
| Male / Stomach - 2 Joinpoints | 1 | 1975 | 1988 | -2.5194* | -3.5409 | -2.2305 |
| Male / Stomach - 2 Joinpoints | 2 | 1988 | 1991 | -0.5701 | -3.5299 | 0.1474 |
| Male / Stomach - 2 Joinpoints | 3 | 1991 | 2020 | -3.3782* | -3.5136 | -3.2458 |
| Male / Kidney and Renal Pelvis - 2 Joinpoints | 1 | 1975 | 1991 | 1.7624* | 1.5374 | 2.0394 |
| Male / Kidney and Renal Pelvis - 2 Joinpoints | 2 | 1991 | 2011 | 0.0046 | -0.1334 | 0.1591 |
| Male / Kidney and Renal Pelvis - 2 Joinpoints | 3 | 2011 | 2020 | -1.3229* | -1.7799 | -0.9912 |
| Male / Liver - 2 Joinpoints | 1 | 1975 | 1997 | 1.9094* | 1.6902 | 2.1291 |
| Male / Liver - 2 Joinpoints | 2 | 1997 | 2006 | 0.3424 | -0.4028 | 1.093 |
| Male / Liver - 2 Joinpoints | 3 | 2006 | 2020 | 2.3609* | 2.1021 | 2.6205 |
| Male / Myeloma - 1 Joinpoint | 1 | 1975 | 1994 | 1.7957* | 1.5703 | 2.0563 |
| Male / Myeloma - 1 Joinpoint | 2 | 1994 | 2020 | -0.7023* | -0.8066 | -0.599 |
| Male / Small Intestine - 2 Joinpoints | 1 | 1975 | 1995 | 1.8072* | 1.3105 | 2.4764 |
| Male / Small Intestine - 2 Joinpoints | 2 | 1995 | 2003 | -2.8419* | -8.114 | -1.3017 |
| Male / Small Intestine - 2 Joinpoints | 3 | 2003 | 2020 | 1.7402* | 1.2753 | 2.3618 |
| Male / Anus, Anal Canal and Anorectum - 2 Joinpoints | 1 | 1975 | 1981 | 17.3210* | 6.6451 | 60.363 |
| Male / Anus, Anal Canal and Anorectum - 2 Joinpoints | 2 | 1981 | 2008 | 1 | -0.3291 | 1.7077 |
| Male / Anus, Anal Canal and Anorectum - 2 Joinpoints | 3 | 2008 | 2020 | 5.6452* | 4.3431 | 8.02 |
| Male / Intrahepatic Bile Duct - 2 Joinpoints | 1 | 1975 | 1986 | 13.4144* | 11.4247 | 21.7359 |
| Male / Intrahepatic Bile Duct - 2 Joinpoints | 2 | 1986 | 1994 | 8.9913* | 3.1647 | 10.8227 |
| Male / Intrahepatic Bile Duct - 2 Joinpoints | 3 | 1994 | 2020 | 3.0422* | 2.8481 | 3.2312 |
| Male / Gallbladder - 2 Joinpoints | 1 | 1975 | 2005 | -2.3454* | -2.6706 | -1.8925 |
| Male / Gallbladder - 2 Joinpoints | 2 | 2005 | 2012 | 0.3455 | -3.5911 | 4.5137 |
| Male / Gallbladder - 2 Joinpoints | 3 | 2012 | 2020 | -2.7353* | -6.0479 | -1.2319 |
| Male / Other Biliary - 2 Joinpoints | 1 | 1975 | 1985 | -0.2712 | -1.1781 | 1.1501 |
| Male / Other Biliary - 2 Joinpoints | 2 | 1985 | 2012 | -3.2234* | -3.5518 | -2.9894 |
| Male / Other Biliary - 2 Joinpoints | 3 | 2012 | 2020 | 2.0975* | 0.7593 | 4.4109 |
| Male / Pancreas - 2 Joinpoints | 1 | 1975 | 1983 | -0.8661* | -2.2273 | -0.412 |
| Male / Pancreas - 2 Joinpoints | 2 | 1983 | 2000 | -0.2127 | -0.3628 | 0.2607 |
| Male / Pancreas - 2 Joinpoints | 3 | 2000 | 2020 | 0.3618* | 0.2768 | 0.5026 |
| Male / Retroperitoneum - 1 Joinpoint | 1 | 1975 | 2010 | -4.0674* | -4.5447 | -3.6728 |
| Male / Retroperitoneum - 1 Joinpoint | 2 | 2010 | 2020 | 5.5314* | 3.1177 | 9.6212 |
| Male / Peritoneum, Omentum and Mesentery - 1 Joinpoint | 1 | 1975 | 2000 | -1.5200* | -3.0851 | -0.5503 |
| Male / Peritoneum, Omentum and Mesentery - 1 Joinpoint | 2 | 2000 | 2020 | 3.0316* | 2.0612 | 4.7824 |
| Male / Other Digestive Organs - 2 Joinpoints | 1 | 1975 | 1996 | -4.2253* | -5.9438 | -3.1214 |
| Male / Other Digestive Organs - 2 Joinpoints | 2 | 1996 | 1999 | 18.3160* | 2.5581 | 25.4609 |
| Male / Other Digestive Organs - 2 Joinpoints | 3 | 1999 | 2020 | 1.1445 | -0.0976 | 1.9166 |
| Male / Nose, Nasal Cavity and Middle Ear - 2 Joinpoints | 1 | 1975 | 1980 | -7.7513* | -17.7436 | -2.6388 |
| Male / Nose, Nasal Cavity and Middle Ear - 2 Joinpoints | 2 | 1980 | 2013 | -2.1484 | -3.4579 | 2.6191 |
| Male / Nose, Nasal Cavity and Middle Ear - 2 Joinpoints | 3 | 2013 | 2020 | 1.0678 | -1.9141 | 10.7527 |
| Male / Larynx - 1 Joinpoint | 1 | 1975 | 1994 | -0.5514* | -0.7919 | -0.2504 |
| Male / Larynx - 1 Joinpoint | 2 | 1994 | 2020 | -2.1706* | -2.3297 | -2.0343 |
| Male / Pleura - 2 Joinpoints | 1 | 1975 | 1992 | 3.4070* | 2.6029 | 4.365 |
| Male / Pleura - 2 Joinpoints | 2 | 1992 | 2010 | -6.1857* | -7.0859 | -5.4783 |
| Male / Pleura - 2 Joinpoints | 3 | 2010 | 2020 | 4.7289* | 3.0834 | 6.8221 |
| Male / Trachea, Mediastinum and Other Respiratory Organs - 2 Joinpoints | 1 | 1975 | 1980 | -10.8492* | -19.6244 | -6.7305 |
| Male / Trachea, Mediastinum and Other Respiratory Organs - 2 Joinpoints | 2 | 1980 | 2011 | -3.8712* | -4.3584 | -3.3075 |
| Male / Trachea, Mediastinum and Other Respiratory Organs - 2 Joinpoints | 3 | 2011 | 2020 | 2.9199* | 0.3247 | 9.0092 |
| Male / Bones and Joints - 2 Joinpoints | 1 | 1975 | 1982 | -11.8627* | -17.3233 | -8.4732 |
| Male / Bones and Joints - 2 Joinpoints | 2 | 1982 | 2012 | -0.6222* | -1.653 | -0.1035 |
| Male / Bones and Joints - 2 Joinpoints | 3 | 2012 | 2020 | 3.1227* | 0.7 | 11.4752 |
| Male / Soft Tissue including Heart - 2 Joinpoints | 1 | 1975 | 1980 | 7.4388* | 2.4423 | 20.8825 |
| Male / Soft Tissue including Heart - 2 Joinpoints | 2 | 1980 | 1993 | 1.8663 | -1.0361 | 3.4428 |
| Male / Soft Tissue including Heart - 2 Joinpoints | 3 | 1993 | 2020 | -0.002 | -2.1749 | 0.9843 |
| Male / Non-Melanoma Skin - 2 Joinpoints | 1 | 1975 | 2004 | 0.3091 | -0.0433 | 0.6111 |
| Male / Non-Melanoma Skin - 2 Joinpoints | 2 | 2004 | 2015 | 2.6268* | 1.8259 | 6.6124 |
| Male / Non-Melanoma Skin - 2 Joinpoints | 3 | 2015 | 2020 | -0.9644 | -4.3035 | 0.834 |
| Male / Testis - 1 Joinpoint | 1 | 1975 | 1995 | -4.6430* | -7.7663 | -3.2244 |
| Male / Testis - 1 Joinpoint | 2 | 1995 | 2020 | 0.1578 | -0.8564 | 2.1595 |
| Male / Penis - 1 Joinpoint | 1 | 1975 | 2001 | -2.0991* | -2.8389 | -1.611 |
| Male / Penis - 1 Joinpoint | 2 | 2001 | 2020 | 0.7337* | 0.0813 | 1.8119 |
| Male / Other Male Genital Organs - 1 Joinpoint | 1 | 1975 | 1994 | -4.5342* | -8.7389 | -2.4866 |
| Male / Other Male Genital Organs - 1 Joinpoint | 2 | 1994 | 2020 | 1.1362* | 0.0795 | 3.4032 |
| Male / Ureter - 0 Joinpoints | 1 | 1975 | 2020 | -0.6787* | -0.8929 | -0.4456 |
| Male / Other Urinary Organs - 2 Joinpoints | 1 | 1975 | 1996 | -2.3392* | -4.4419 | -0.8812 |
| Male / Other Urinary Organs - 2 Joinpoints | 2 | 1996 | 2000 | 23.8915* | 9.7065 | 40.3454 |
| Male / Other Urinary Organs - 2 Joinpoints | 3 | 2000 | 2020 | 1.1121* | 0.0554 | 1.9496 |
| Male / Eye and Orbit - 1 Joinpoint | 1 | 1975 | 2004 | -2.8477* | -3.5069 | -2.3571 |
| Male / Eye and Orbit - 1 Joinpoint | 2 | 2004 | 2020 | 1.1789* | 0.1044 | 3.0122 |
| Male / Brain and Other Nervous System - 2 Joinpoints | 1 | 1975 | 1990 | 2.6877* | 2.316 | 3.13 |
| Male / Brain and Other Nervous System - 2 Joinpoints | 2 | 1990 | 2006 | -0.3930* | -1.2614 | -0.1005 |
| Male / Brain and Other Nervous System - 2 Joinpoints | 3 | 2006 | 2020 | 0.6027* | 0.3164 | 1.2532 |
| Male / Endocrine System - 0 Joinpoints | 1 | 1975 | 2020 | 0.9352* | 0.8148 | 1.0904 |
| Male / Hodgkin Lymphoma - 1 Joinpoint | 1 | 1975 | 1988 | -4.3110* | -7.0676 | -3.2118 |
| Male / Hodgkin Lymphoma - 1 Joinpoint | 2 | 1988 | 2020 | -1.4518* | -1.7566 | -0.9936 |
| Female / Lung and Bronchus - 2 Joinpoints | 1 | 1975 | 1992 | 6.6271* | 6.2933 | 6.9619 |
| Female / Lung and Bronchus - 2 Joinpoints | 2 | 1992 | 2008 | 1.0674* | 0.8293 | 1.3062 |
| Female / Lung and Bronchus - 2 Joinpoints | 3 | 2008 | 2020 | -3.0819* | -3.3879 | -2.775 |
| Female / Breast - 2 Joinpoints | 1 | 1975 | 1991 | 1.0258* | 0.8708 | 1.1865 |
| Female / Breast - 2 Joinpoints | 2 | 1991 | 2009 | -1.6419* | -1.8885 | -1.5298 |
| Female / Breast - 2 Joinpoints | 3 | 2009 | 2020 | -1.1153* | -1.3391 | -0.5637 |
| Female / Colon and Rectum - 2 Joinpoints | 1 | 1975 | 1984 | -0.7145* | -1.0387 | -0.3893 |
| Female / Colon and Rectum - 2 Joinpoints | 2 | 1984 | 2000 | -1.7412* | -1.8798 | -1.6024 |
| Female / Colon and Rectum - 2 Joinpoints | 3 | 2000 | 2020 | -3.1583* | -3.2555 | -3.061 |
| Female / Pancreas - 2 Joinpoints | 1 | 1975 | 1984 | 1.2764* | 0.9024 | 1.6518 |
| Female / Pancreas - 2 Joinpoints | 2 | 1984 | 2010 | 0.2906* | 0.2266 | 0.3547 |
| Female / Pancreas - 2 Joinpoints | 3 | 2010 | 2020 | 0.0126 | -0.2026 | 0.2283 |
| Female / Ovary - 2 Joinpoints | 1 | 1975 | 1992 | 1.0952* | 0.8884 | 1.3024 |
| Female / Ovary - 2 Joinpoints | 2 | 1992 | 2006 | -0.0105 | -0.2683 | 0.2479 |
| Female / Ovary - 2 Joinpoints | 3 | 2006 | 2020 | -2.5943* | -2.8196 | -2.3686 |
| Female / Non-Hodgkin Lymphoma - 2 Joinpoints | 1 | 1975 | 1989 | 3.2905* | 3.0593 | 3.6224 |
| Female / Non-Hodgkin Lymphoma - 2 Joinpoints | 2 | 1989 | 1997 | 1.9785* | 1.1754 | 2.2965 |
| Female / Non-Hodgkin Lymphoma - 2 Joinpoints | 3 | 1997 | 2020 | -2.4438* | -2.5292 | -2.3681 |
| Female / Leukemia - 2 Joinpoints | 1 | 1975 | 2000 | 0.2965* | 0.2027 | 0.4211 |
| Female / Leukemia - 2 Joinpoints | 2 | 2000 | 2013 | -0.8118* | -1.065 | -0.4475 |
| Female / Leukemia - 2 Joinpoints | 3 | 2013 | 2020 | -2.0216* | -3.0872 | -1.533 |
| Female / Stomach - 2 Joinpoints | 1 | 1975 | 1986 | -2.9815* | -3.5791 | -2.7005 |
| Female / Stomach - 2 Joinpoints | 2 | 1986 | 1991 | -0.9936 | -1.9399 | 0.5128 |
| Female / Stomach - 2 Joinpoints | 3 | 1991 | 2020 | -3.0201* | -3.1099 | -2.9477 |
| Female / Myeloma - 2 Joinpoints | 1 | 1975 | 1992 | 2.0149* | 1.6714 | 2.3595 |
| Female / Myeloma - 2 Joinpoints | 2 | 1992 | 2000 | -0.0409 | -1.0558 | 0.9845 |
| Female / Myeloma - 2 Joinpoints | 3 | 2000 | 2020 | -1.1271* | -1.3273 | -0.9265 |
| Female / Urinary Bladder - 2 Joinpoints | 1 | 1975 | 1986 | -1.5182* | -2.7259 | -0.9642 |
| Female / Urinary Bladder - 2 Joinpoints | 2 | 1986 | 2016 | -0.4122 | -0.5101 | 0.1105 |
| Female / Urinary Bladder - 2 Joinpoints | 3 | 2016 | 2020 | -2.3897* | -5.28 | -0.7075 |
| Female / Kidney and Renal Pelvis - 2 Joinpoints | 1 | 1975 | 1992 | 1.6746* | 1.4462 | 1.9864 |
| Female / Kidney and Renal Pelvis - 2 Joinpoints | 2 | 1992 | 2006 | 0.1705 | -0.1403 | 0.4822 |
| Female / Kidney and Renal Pelvis - 2 Joinpoints | 3 | 2006 | 2020 | -1.5531* | -1.832 | -1.3223 |
| Female / Brain and Other Nervous System - 2 Joinpoints | 1 | 1975 | 1991 | 2.8225* | 2.4686 | 3.2458 |
| Female / Brain and Other Nervous System - 2 Joinpoints | 2 | 1991 | 2006 | -0.5845* | -1.4547 | -0.2601 |
| Female / Brain and Other Nervous System - 2 Joinpoints | 3 | 2006 | 2020 | 0.5071* | 0.1906 | 1.1525 |
| Female / Small Intestine - 2 Joinpoints | 1 | 1975 | 1991 | 1.2378* | 0.4474 | 3.6722 |
| Female / Small Intestine - 2 Joinpoints | 2 | 1991 | 2013 | -0.4662* | -3.9085 | -0.065 |
| Female / Small Intestine - 2 Joinpoints | 3 | 2013 | 2020 | 3.8974* | 1.9515 | 7.6168 |
| Female / Anus, Anal Canal and Anorectum - 2 Joinpoints | 1 | 1975 | 1980 | 41.8427* | 28.7298 | 64.3805 |
| Female / Anus, Anal Canal and Anorectum - 2 Joinpoints | 2 | 1980 | 2007 | 0.8527* | 0.2634 | 1.3522 |
| Female / Anus, Anal Canal and Anorectum - 2 Joinpoints | 3 | 2007 | 2020 | 5.0365* | 4.1231 | 6.4932 |
| Female / Intrahepatic Bile Duct - 2 Joinpoints | 1 | 1975 | 1986 | 13.2799* | 11.5818 | 17.3642 |
| Female / Intrahepatic Bile Duct - 2 Joinpoints | 2 | 1986 | 1996 | 7.5866* | 5.9256 | 9.0595 |
| Female / Intrahepatic Bile Duct - 2 Joinpoints | 3 | 1996 | 2020 | 3.2833* | 3.0934 | 3.4742 |
| Female / Gallbladder - 2 Joinpoints | 1 | 1975 | 1989 | -3.4300* | -5.3076 | -2.9821 |
| Female / Gallbladder - 2 Joinpoints | 2 | 1989 | 2002 | -2.5214* | -3.1744 | -1.1288 |
| Female / Gallbladder - 2 Joinpoints | 3 | 2002 | 2020 | -1.4342 | -2.0262 | 0.3085 |
| Female / Other Biliary - 2 Joinpoints | 1 | 1975 | 1981 | 1.2625 | -0.4039 | 4.0208 |
| Female / Other Biliary - 2 Joinpoints | 2 | 1981 | 2009 | -3.0309* | -3.2748 | -2.8485 |
| Female / Other Biliary - 2 Joinpoints | 3 | 2009 | 2020 | 0.4575 | -0.2954 | 1.5537 |
| Female / Pancreas - 1 Joinpoint | 1 | 1975 | 1986 | 1.1379* | 0.8566 | 1.5672 |
| Female / Pancreas - 1 Joinpoint | 2 | 1986 | 2020 | 0.2153* | 0.1686 | 0.2589 |
| Female / Retroperitoneum - 1 Joinpoint | 1 | 1975 | 2013 | -3.6841* | -4.0886 | -3.3353 |
| Female / Retroperitoneum - 1 Joinpoint | 2 | 2013 | 2020 | 8.1590* | 3.2267 | 19.7566 |
| Female / Peritoneum, Omentum and Mesentery - 2 Joinpoints | 1 | 1975 | 1985 | -6.6609* | -10.5478 | -3.754 |
| Female / Peritoneum, Omentum and Mesentery - 2 Joinpoints | 2 | 1985 | 2003 | 9.9381* | 8.9928 | 11.4464 |
| Female / Peritoneum, Omentum and Mesentery - 2 Joinpoints | 3 | 2003 | 2020 | 0.6146* | 0.0469 | 1.1879 |
| Female / Other Digestive Organs - 2 Joinpoints | 1 | 1975 | 1996 | -4.2031* | -5.443 | -3.3452 |
| Female / Other Digestive Organs - 2 Joinpoints | 2 | 1996 | 1999 | 16.6581* | 3.3463 | 22.2618 |
| Female / Other Digestive Organs - 2 Joinpoints | 3 | 1999 | 2020 | 0.9451 | -0.1536 | 1.6321 |
| Female / Nose, Nasal Cavity and Middle Ear - 1 Joinpoint | 1 | 1975 | 1981 | -6.3367* | -16.1395 | -2.0539 |
| Female / Nose, Nasal Cavity and Middle Ear - 1 Joinpoint | 2 | 1981 | 2020 | -1.3216* | -1.5992 | -0.6395 |
| Female / Larynx - 2 Joinpoints | 1 | 1975 | 1992 | 3.5046* | 2.6229 | 8.3735 |
| Female / Larynx - 2 Joinpoints | 2 | 1992 | 1999 | 0.0409 | -2.4512 | 3.6458 |
| Female / Larynx - 2 Joinpoints | 3 | 1999 | 2020 | -2.5220* | -4.9844 | -2.0597 |
| Female / Pleura - 2 Joinpoints | 1 | 1975 | 1996 | -1.2179 | -2.1434 | 0.4288 |
| Female / Pleura - 2 Joinpoints | 2 | 1996 | 2009 | -6.2966* | -19.5171 | -4.2114 |
| Female / Pleura - 2 Joinpoints | 3 | 2009 | 2020 | 5.9488* | 2.7995 | 11.2852 |
| Female / Trachea, Mediastinum and Other Respiratory Organs - 1 Joinpoint | 1 | 1975 | 2012 | -2.8427* | -3.8043 | -2.3927 |
| Female / Trachea, Mediastinum and Other Respiratory Organs - 1 Joinpoint | 2 | 2012 | 2020 | 2.911 | -1.4505 | 18.0404 |
| Female / Bones and Joints - 2 Joinpoints | 1 | 1975 | 1981 | -12.7795* | -16.9326 | -9.6582 |
| Female / Bones and Joints - 2 Joinpoints | 2 | 1981 | 2012 | -0.5028* | -1.0059 | -0.139 |
| Female / Bones and Joints - 2 Joinpoints | 3 | 2012 | 2020 | 3.0814* | 1.1723 | 8.8743 |
| Female / Soft Tissue including Heart - 2 Joinpoints | 1 | 1975 | 1997 | 2.7013* | 2.3765 | 3.1569 |
| Female / Soft Tissue including Heart - 2 Joinpoints | 2 | 1997 | 2000 | -8.2685* | -9.8706 | -3.3291 |
| Female / Soft Tissue including Heart - 2 Joinpoints | 3 | 2000 | 2020 | -0.0789 | -0.3959 | 0.3777 |
| Female / Non-Melanoma Skin - 2 Joinpoints | 1 | 1975 | 1981 | -6.7183* | -11.8096 | -4.1052 |
| Female / Non-Melanoma Skin - 2 Joinpoints | 2 | 1981 | 2011 | 0.1107 | -0.2209 | 0.3915 |
| Female / Non-Melanoma Skin - 2 Joinpoints | 3 | 2011 | 2020 | 2.6097* | 1.5595 | 5.6272 |
| Female / Cervix Uteri - 2 Joinpoints | 1 | 1975 | 1981 | -4.6007* | -7.0631 | -3.5651 |
| Female / Cervix Uteri - 2 Joinpoints | 2 | 1981 | 2007 | -2.8662* | -3.0326 | -2.6403 |
| Female / Cervix Uteri - 2 Joinpoints | 3 | 2007 | 2020 | -1.1860* | -1.6104 | -0.5109 |
| Female / Uterus, NOS - 2 Joinpoints | 1 | 1975 | 1996 | -1.4343* | -1.7949 | -1.1473 |
| Female / Uterus, NOS - 2 Joinpoints | 2 | 1996 | 2015 | 1.3448* | 1.0559 | 1.7488 |
| Female / Uterus, NOS - 2 Joinpoints | 3 | 2015 | 2020 | -6.3259* | -8.3902 | -4.6677 |
| Female / Vagina - 0 Joinpoints | 1 | 1975 | 2020 | -1.2873* | -1.4243 | -1.1439 |
| Female / Vulva - 2 Joinpoints | 1 | 1975 | 1984 | -2.3105* | -4.8748 | -1.2743 |
| Female / Vulva - 2 Joinpoints | 2 | 1984 | 2010 | 0.1749 | -0.0749 | 0.4593 |
| Female / Vulva - 2 Joinpoints | 3 | 2010 | 2020 | 2.3737* | 1.74 | 3.7213 |
| Female / Other Female Genital Organs - 2 Joinpoints | 1 | 1975 | 2002 | 1.7349* | 1.0141 | 10.6583 |
| Female / Other Female Genital Organs - 2 Joinpoints | 2 | 2002 | 2005 | -7.5697 | -11.6725 | 10.0882 |
| Female / Other Female Genital Organs - 2 Joinpoints | 3 | 2005 | 2020 | 6.9778* | 4.4485 | 9.0894 |
| Female / Ureter - 0 Joinpoints | 1 | 1975 | 2020 | -0.2909* | -0.4834 | -0.0706 |
| Female / Other Urinary Organs - 2 Joinpoints | 1 | 1975 | 1997 | -2.1506* | -3.6122 | -1.3226 |
| Female / Other Urinary Organs - 2 Joinpoints | 2 | 1997 | 2000 | 15.8679* | 3.3663 | 21.2046 |
| Female / Other Urinary Organs - 2 Joinpoints | 3 | 2000 | 2020 | -0.0911 | -1.0523 | 0.5349 |
| Female / Eye and Orbit - 1 Joinpoint | 1 | 1975 | 2004 | -3.0080* | -3.6054 | -2.5376 |
| Female / Eye and Orbit - 1 Joinpoint | 2 | 2004 | 2020 | 1.2781* | 0.0592 | 3.1246 |
| Female / Brain and Other Nervous System - 2 Joinpoints | 1 | 1975 | 1991 | 2.8225* | 2.4686 | 3.2458 |
| Female / Brain and Other Nervous System - 2 Joinpoints | 2 | 1991 | 2006 | -0.5845* | -1.4547 | -0.2601 |
| Female / Brain and Other Nervous System - 2 Joinpoints | 3 | 2006 | 2020 | 0.5071* | 0.1906 | 1.1525 |
| Female / Endocrine System - 1 Joinpoint | 1 | 1975 | 1985 | -1.7966* | -3.5183 | -0.9395 |
| Female / Endocrine System - 1 Joinpoint | 2 | 1985 | 2020 | 0.2244* | 0.1115 | 0.3737 |
| Female / Hodgkin Lymphoma - 1 Joinpoint | 1 | 1975 | 1988 | -3.9042* | -6.6802 | -2.9579 |
| Female / Hodgkin Lymphoma - 1 Joinpoint | 2 | 1988 | 2020 | -1.8375* | -2.1251 | -1.3554 |

*Indicate that the Annual Percentage Change (APC) is significantly different from zero at the alpha=0.05 level (P value is not available for the Empirical Quantile method).
