# Supplementary material for: Trends analysis of cancer incidence, mortality, and survival for the elderly in the United States, 1975–2020
Source: Cancer Med. 2024 Jul 31;13(15):e70062. doi: 10.1002/cam4.70062 (PMC11289898; doi:10.1002/cam4.70062)
Supplement: Supplementary file 1 — Appendix S1. [file CAM4-13-e70062-s001.zip › Supplementary Table 14 Trends in 1.docx]

**Supplementary Table 14** Trends in 1-, 3- and 5-year survival rate, United States, 1975-2019.

| Interval | Start Year | End Year | Estimate Average Absolute Change %* | Standard Error % | Lower Limit 95% C.I. | Upper Limit 95% C.I. | Significance |
| --- | --- | --- | --- | --- | --- | --- | --- |
| Total |  |  |  |  |  |  |  |
| 1 | 1975 | 1983 | 0.25 | 0.08 | 0.1 | 0.4 | Increasing |
| 1 | 1983 | 1991 | 0.91 | 0.05 | 0.81 | 1 | Increasing |
| 1 | 1991 | 2019 | 0.19 | 0.01 | 0.17 | 0.21 | Increasing |
| 3 | 1975 | 1983 | 0.31 | 0.1 | 0.13 | 0.5 | Increasing |
| 3 | 1983 | 1991 | 1.17 | 0.06 | 1.06 | 1.29 | Increasing |
| 3 | 1991 | 2019 | 0.26 | 0.01 | 0.24 | 0.29 | Increasing |
| 5 | 1975 | 1983 | 0.33 | 0.1 | 0.13 | 0.53 | Increasing |
| 5 | 1983 | 1991 | 1.25 | 0.06 | 1.13 | 1.38 | Increasing |
| 5 | 1991 | 2019 | 0.29 | 0.01 | 0.26 | 0.31 | Increasing |
| Oral cavity & pharynx |  |  |  |  |  |  |  |
| 1 | 1975 | 2005 | 0.09 | 0.02 | 0.04 | 0.14 | Increasing |
| 1 | 2005 | 2018 | 0.6 | 0.06 | 0.48 | 0.72 | Increasing |
| 3 | 1975 | 2005 | 0.13 | 0.04 | 0.06 | 0.21 | Increasing |
| 3 | 2005 | 2018 | 1 | 0.1 | 0.8 | 1.19 | Increasing |
| 5 | 1975 | 2005 | 0.15 | 0.04 | 0.06 | 0.23 | Increasing |
| 5 | 2005 | 2018 | 1.12 | 0.11 | 0.9 | 1.35 | Increasing |
| Esophagus |  |  |  |  |  |  |  |
| 1 | 1975 | 2019 | 0.64 | 0.03 | 0.59 | 0.69 | Increasing |
| 3 | 1975 | 2019 | 0.53 | 0.02 | 0.49 | 0.57 | Increasing |
| 5 | 1975 | 2019 | 0.46 | 0.02 | 0.43 | 0.5 | Increasing |
| Stomach |  |  |  |  |  |  |  |
| 1 | 1975 | 2005 | 0.4 | 0.03 | 0.34 | 0.46 | Increasing |
| 1 | 2005 | 2019 | 0.83 | 0.07 | 0.69 | 0.97 | Increasing |
| 3 | 1975 | 2005 | 0.38 | 0.03 | 0.32 | 0.43 | Increasing |
| 3 | 2005 | 2019 | 0.92 | 0.08 | 0.77 | 1.08 | Increasing |
| 5 | 1975 | 2005 | 0.35 | 0.03 | 0.3 | 0.4 | Increasing |
| 5 | 2005 | 2019 | 0.91 | 0.08 | 0.76 | 1.07 | Increasing |
| Liver |  |  |  |  |  |  |  |
| 1 | 1975 | 2019 | 1.08 | 0.03 | 1.03 | 1.13 | Increasing |
| 3 | 1975 | 2019 | 0.8 | 0.02 | 0.76 | 0.84 | Increasing |
| 5 | 1975 | 2019 | 0.66 | 0.02 | 0.62 | 0.69 | Increasing |
| Pancreas |  |  |  |  |  |  |  |
| 1 | 1975 | 2018 | 0.48 | 0.02 | 0.43 | 0.52 | Increasing |
| 3 | 1975 | 2018 | 0.24 | 0.01 | 0.21 | 0.26 | Increasing |
| 5 | 1975 | 2018 | 0.18 | 0.01 | 0.17 | 0.2 | Increasing |
| Colon & rectum |  |  |  |  |  |  |  |
| 1 | 1975 | 1988 | 0.62 | 0.04 | 0.55 | 0.7 | Increasing |
| 1 | 1988 | 2008 | 0.11 | 0.02 | 0.08 | 0.14 | Increasing |
| 1 | 2008 | 2019 | -0.15 | 0.04 | -0.24 | -0.07 | Decreasing |
| 3 | 1975 | 1988 | 0.9 | 0.05 | 0.8 | 1 | Increasing |
| 3 | 1988 | 2008 | 0.17 | 0.03 | 0.12 | 0.22 | Increasing |
| 3 | 2008 | 2019 | -0.23 | 0.07 | -0.37 | -0.1 | Decreasing |
| 5 | 1975 | 1988 | 0.99 | 0.06 | 0.88 | 1.1 | Increasing |
| 5 | 1988 | 2008 | 0.19 | 0.03 | 0.13 | 0.25 | Increasing |
| 5 | 2008 | 2019 | -0.27 | 0.08 | -0.42 | -0.11 | Decreasing |
| Lung & bronchus |  |  |  |  |  |  |  |
| 1 | 1975 | 2002 | 0.28 | 0.02 | 0.25 | 0.32 | Increasing |
| 1 | 2002 | 2013 | 0.73 | 0.04 | 0.65 | 0.81 | Increasing |
| 1 | 2013 | 2019 | 1.77 | 0.11 | 1.55 | 1.99 | Increasing |
| 3 | 1975 | 2002 | 0.23 | 0.01 | 0.2 | 0.26 | Increasing |
| 3 | 2002 | 2013 | 0.69 | 0.04 | 0.61 | 0.77 | Increasing |
| 3 | 2013 | 2019 | 1.94 | 0.13 | 1.69 | 2.19 | Increasing |
| 5 | 1975 | 2002 | 0.2 | 0.01 | 0.17 | 0.22 | Increasing |
| 5 | 2002 | 2013 | 0.63 | 0.04 | 0.56 | 0.71 | Increasing |
| 5 | 2013 | 2019 | 1.88 | 0.12 | 1.64 | 2.12 | Increasing |
| Melanoma |  |  |  |  |  |  |  |
| 1 | 1975 | 2004 | 0.19 | 0.02 | 0.15 | 0.24 | Increasing |
| 3 | 1975 | 2004 | 0.45 | 0.05 | 0.35 | 0.56 | Increasing |
| 5 | 1975 | 2004 | 0.56 | 0.07 | 0.43 | 0.69 | Increasing |
| Corpus uteri |  |  |  |  |  |  |  |
| 1 | 1975 | 2013 | 0.05 | 0.02 | 0.01 | 0.08 | Increasing |
| 3 | 1975 | 2013 | 0.09 | 0.03 | 0.02 | 0.15 | Increasing |
| 5 | 1975 | 2013 | 0.1 | 0.04 | 0.02 | 0.17 | Increasing |
| Ovary |  |  |  |  |  |  |  |
| 1 | 1975 | 2019 | 0.43 | 0.03 | 0.37 | 0.48 | Increasing |
| 3 | 1975 | 2019 | 0.53 | 0.03 | 0.47 | 0.6 | Increasing |
| 5 | 1975 | 2019 | 0.52 | 0.03 | 0.46 | 0.59 | Increasing |
| Breast |  |  |  |  |  |  |  |
| 1 | 1975 | 1985 | 0.1 | 0.03 | 0.04 | 0.16 | Increasing |
| 1 | 1985 | 1988 | 0.71 | 0.06 | 0.59 | 0.83 | Increasing |
| 1 | 1988 | 2019 | 0.05 | 0 | 0.04 | 0.05 | Increasing |
| 3 | 1975 | 1985 | 0.25 | 0.07 | 0.1 | 0.4 | Increasing |
| 3 | 1985 | 1988 | 1.8 | 0.15 | 1.51 | 2.1 | Increasing |
| 3 | 1988 | 2019 | 0.12 | 0.01 | 0.1 | 0.14 | Increasing |
| 5 | 1975 | 1985 | 0.36 | 0.11 | 0.15 | 0.56 | Increasing |
| 5 | 1985 | 1988 | 2.63 | 0.21 | 2.21 | 3.05 | Increasing |
| 5 | 1988 | 2019 | 0.18 | 0.01 | 0.15 | 0.21 | Increasing |
| Kidney & renal pelvis |  |  |  |  |  |  |  |
| 1 | 1975 | 1981 | -0.41 | 0.31 | -1.02 | 0.2 | Not significant |
| 1 | 1981 | 2016 | 0.54 | 0.03 | 0.48 | 0.6 | Increasing |
| 3 | 1975 | 1981 | -0.53 | 0.39 | -1.3 | 0.25 | Not significant |
| 3 | 1981 | 2016 | 0.73 | 0.04 | 0.66 | 0.81 | Increasing |
| 5 | 1975 | 1981 | -0.56 | 0.42 | -1.39 | 0.26 | Not significant |
| 5 | 1981 | 2016 | 0.81 | 0.04 | 0.73 | 0.9 | Increasing |
| Urinary bladder |  |  |  |  |  |  |  |
| 1 | 1975 | 1989 | 0.41 | 0.04 | 0.34 | 0.49 | Increasing |
| 1 | 1989 | 2019 | 0.04 | 0.01 | 0.02 | 0.06 | Increasing |
| 3 | 1975 | 1989 | 0.66 | 0.06 | 0.54 | 0.78 | Increasing |
| 3 | 1989 | 2019 | 0.06 | 0.02 | 0.03 | 0.1 | Increasing |
| 5 | 1975 | 1989 | 0.77 | 0.07 | 0.63 | 0.91 | Increasing |
| 5 | 1989 | 2019 | 0.07 | 0.02 | 0.03 | 0.12 | Increasing |
| Prostate |  |  |  |  |  |  |  |
| 1 | 1975 | 1985 | 0.17 | 0.04 | 0.09 | 0.25 | Increasing |
| 1 | 1985 | 1989 | 0.61 | 0.07 | 0.46 | 0.75 | Increasing |
| 1 | 1989 | 2019 | 0.08 | 0.01 | 0.07 | 0.1 | Increasing |
| 3 | 1975 | 1985 | 0.49 | 0.12 | 0.26 | 0.72 | Increasing |
| 3 | 1985 | 1989 | 1.83 | 0.21 | 1.41 | 2.24 | Increasing |
| 3 | 1989 | 2019 | 0.27 | 0.02 | 0.23 | 0.3 | Increasing |
| 5 | 1975 | 1985 | 0.7 | 0.17 | 0.38 | 1.03 | Increasing |
| 5 | 1985 | 1989 | 2.74 | 0.31 | 2.13 | 3.35 | Increasing |
| 5 | 1989 | 2019 | 0.42 | 0.03 | 0.37 | 0.48 | Increasing |
| Brain & other nervous system |  |  |  |  |  |  |  |
| 1 | 1975 | 1999 | 0.2 | 0.08 | 0.05 | 0.35 | Increasing |
| 1 | 1999 | 2019 | 0.77 | 0.09 | 0.6 | 0.94 | Increasing |
| 3 | 1975 | 1999 | 0.09 | 0.03 | 0.03 | 0.16 | Increasing |
| 3 | 1999 | 2019 | 0.51 | 0.06 | 0.39 | 0.63 | Increasing |
| 5 | 1975 | 1999 | 0.07 | 0.03 | 0.02 | 0.13 | Increasing |
| 5 | 1999 | 2019 | 0.44 | 0.05 | 0.33 | 0.54 | Increasing |
| Non-Hodgkin lymphoma |  |  |  |  |  |  |  |
| 1 | 1975 | 1996 | 0.57 | 0.06 | 0.44 | 0.69 | Increasing |
| 1 | 1996 | 2005 | 1.86 | 0.19 | 1.48 | 2.24 | Increasing |
| 3 | 1975 | 1996 | 0.73 | 0.09 | 0.56 | 0.89 | Increasing |
| 3 | 1996 | 2005 | 2.71 | 0.28 | 2.16 | 3.25 | Increasing |
| 5 | 1975 | 1996 | 0.77 | 0.09 | 0.59 | 0.95 | Increasing |
| 5 | 1996 | 2005 | 3.14 | 0.33 | 2.49 | 3.79 | Increasing |
| Myeloma |  |  |  |  |  |  |  |
| 1 | 1975 | 2000 | 0.19 | 0.05 | 0.1 | 0.28 | Increasing |
| 1 | 2000 | 2018 | 0.94 | 0.04 | 0.86 | 1.03 | Increasing |
| 3 | 1975 | 2000 | 0.25 | 0.06 | 0.13 | 0.37 | Increasing |
| 3 | 2000 | 2018 | 1.53 | 0.07 | 1.4 | 1.67 | Increasing |
| 5 | 1975 | 2000 | 0.24 | 0.06 | 0.13 | 0.35 | Increasing |
| 5 | 2000 | 2018 | 1.73 | 0.08 | 1.58 | 1.89 | Increasing |
| Leukemia |  |  |  |  |  |  |  |
| 1 | 1975 | 1997 | 0.51 | 0.06 | 0.39 | 0.64 | Increasing |
| 1 | 1997 | 2018 | 0.81 | 0.06 | 0.68 | 0.93 | Increasing |
| 3 | 1975 | 1997 | 0.57 | 0.07 | 0.43 | 0.72 | Increasing |
| 3 | 1997 | 2018 | 1.01 | 0.08 | 0.85 | 1.16 | Increasing |
| 5 | 1975 | 1997 | 0.58 | 0.07 | 0.43 | 0.72 | Increasing |
| 5 | 1997 | 2018 | 1.09 | 0.09 | 0.92 | 1.26 | Increasing |
| Small intestine |  |  |  |  |  |  |  |
| 1 | 1975 | 2019 | 0.53 | 0.05 | 0.43 | 0.63 | Increasing |
| 3 | 1975 | 2019 | 0.68 | 0.06 | 0.56 | 0.8 | Increasing |
| 5 | 1975 | 2019 | 0.73 | 0.06 | 0.61 | 0.85 | Increasing |
| Anus,anal canal and anorectum |  |  |  |  |  |  |  |
| 1 | 1975 | 2019 | 0.17 | 0.04 | 0.09 | 0.25 | Increasing |
| 3 | 1975 | 2019 | 0.3 | 0.07 | 0.16 | 0.43 | Increasing |
| 5 | 1975 | 2019 | 0.34 | 0.08 | 0.19 | 0.49 | Increasing |
| Intrahepatic bile duct |  |  |  |  |  |  |  |
| 1 | 1975 | 2019 | 0.6 | 0.06 | 0.48 | 0.72 | Increasing |
| 3 | 1975 | 2019 | 0.33 | 0.03 | 0.27 | 0.39 | Increasing |
| 5 | 1975 | 2019 | 0.22 | 0.03 | 0.18 | 0.27 | Increasing |
| Gallbladder |  |  |  |  |  |  |  |
| 1 | 1975 | 2019 | 0.51 | 0.04 | 0.42 | 0.6 | Increasing |
| 3 | 1975 | 2019 | 0.42 | 0.04 | 0.35 | 0.5 | Increasing |
| 5 | 1975 | 2019 | 0.38 | 0.03 | 0.31 | 0.45 | Increasing |
| Other biliary |  |  |  |  |  |  |  |
| 1 | 1975 | 1991 | 1.08 | 0.15 | 0.78 | 1.38 | Increasing |
| 1 | 1991 | 2010 | -0.14 | 0.1 | -0.33 | 0.06 | Not significant |
| 1 | 2010 | 2019 | 0.91 | 0.24 | 0.44 | 1.37 | Increasing |
| 3 | 1975 | 1991 | 0.84 | 0.11 | 0.62 | 1.06 | Increasing |
| 3 | 1991 | 2010 | -0.13 | 0.09 | -0.31 | 0.05 | Not significant |
| 3 | 2010 | 2019 | 0.88 | 0.23 | 0.42 | 1.33 | Increasing |
| 5 | 1975 | 1991 | 0.72 | 0.1 | 0.53 | 0.91 | Increasing |
| 5 | 1991 | 2010 | -0.11 | 0.08 | -0.28 | 0.05 | Not significant |
| 5 | 2010 | 2019 | 0.81 | 0.22 | 0.38 | 1.23 | Increasing |
| Retroperitoneum |  |  |  |  |  |  |  |
| 1 | 1975 | 2019 | 0.6 | 0.09 | 0.42 | 0.78 | Increasing |
| 3 | 1975 | 2019 | 0.74 | 0.11 | 0.53 | 0.95 | Increasing |
| 5 | 1975 | 2019 | 0.75 | 0.11 | 0.54 | 0.96 | Increasing |
| Peritoneum, omentum and mesentery |  |  |  |  |  |  |  |
| 1 | 1975 | 1985 | -2.86 | 0.6 | -4.03 | -1.69 | Decreasing |
| 1 | 1985 | 2019 | 0.33 | 0.1 | 0.13 | 0.52 | Increasing |
| 3 | 1975 | 1985 | -5.17 | 2.08 | -9.25 | -1.09 | Decreasing |
| 3 | 1985 | 2019 | 0.49 | 0.41 | -0.31 | 1.28 | Not significant |
| 5 | 1975 | 1985 | -5.9 | 3.22 | -12.2 | 0.41 | Not significant |
| 5 | 1985 | 2019 | 0.46 | 0.37 | -0.27 | 1.19 | Not significant |
| Other digestive organs |  |  |  |  |  |  |  |
| 1 | 1975 | 2019 | 0.37 | 0.05 | 0.27 | 0.47 | Increasing |
| 3 | 1975 | 2019 | 0.22 | 0.03 | 0.16 | 0.28 | Increasing |
| 5 | 1975 | 2019 | 0.18 | 0.03 | 0.13 | 0.23 | Increasing |
| Nose, nasal cavity and middle ear |  |  |  |  |  |  |  |
| 1 | 1975 | 2019 | 0.22 | 0.06 | 0.1 | 0.35 | Increasing |
| 3 | 1975 | 2019 | 0.33 | 0.09 | 0.15 | 0.51 | Increasing |
| 5 | 1975 | 2019 | 0.36 | 0.1 | 0.17 | 0.56 | Increasing |
| Larynx |  |  |  |  |  |  |  |
| 1 | 1975 | 2019 | -0.01 | 0.02 | -0.04 | 0.03 | Not significant |
| 3 | 1975 | 2019 | -0.01 | 0.04 | -0.08 | 0.06 | Not significant |
| 5 | 1975 | 2019 | -0.01 | 0.04 | -0.09 | 0.07 | Not significant |
| Pleura |  |  |  |  |  |  |  |
| 1 | 1975 | 2019 | 0.7 | 0.27 | 0.18 | 1.22 | Increasing |
| 3 | 1975 | 2019 | 0.58 | 0.22 | 0.14 | 1.02 | Increasing |
| 5 | 1975 | 2019 | 0.58 | 0.22 | 0.14 | 1.02 | Increasing |
| Trachea mediastinum and other respiratory organs |  |  |  |  |  |  |  |
| 1 | 1975 | 2006 | -0.31 | 0.27 | -0.83 | 0.22 | Not significant |
| 1 | 2006 | 2019 | 3.96 | 0.61 | 2.76 | 5.16 | Increasing |
| 3 | 1975 | 2006 | -0.27 | 0.24 | -0.74 | 0.19 | Not significant |
| 3 | 2006 | 2019 | 4.57 | 0.7 | 3.2 | 5.94 | Increasing |
| 5 | 1975 | 2006 | -0.24 | 0.21 | -0.66 | 0.17 | Not significant |
| 5 | 2006 | 2019 | 4.65 | 0.73 | 3.21 | 6.09 | Increasing |
| Bones and joints |  |  |  |  |  |  |  |
| 1 | 1975 | 2019 | 0.42 | 0.09 | 0.25 | 0.59 | Increasing |
| 3 | 1975 | 2019 | 0.53 | 0.11 | 0.32 | 0.74 | Increasing |
| 5 | 1975 | 2019 | 0.57 | 0.11 | 0.35 | 0.79 | Increasing |
| Soft tissue including heart |  |  |  |  |  |  |  |
| 1 | 1975 | 2019 | 0.11 | 0.03 | 0.04 | 0.18 | Increasing |
| 3 | 1975 | 2019 | 0.16 | 0.05 | 0.06 | 0.25 | Increasing |
| 5 | 1975 | 2019 | 0.17 | 0.05 | 0.07 | 0.27 | Increasing |
| Other epithelial skin |  |  |  |  |  |  |  |
| 1 | 1975 | 2019 | 0.03 | 0.03 | -0.03 | 0.08 | Not significant |
| 3 | 1975 | 2019 | 0.06 | 0.06 | -0.06 | 0.17 | Not significant |
| 5 | 1975 | 2019 | 0.07 | 0.07 | -0.07 | 0.22 | Not significant |
| Cervix uteri |  |  |  |  |  |  |  |
| 1 | 1975 | 2019 | -0.02 | 0.03 | -0.08 | 0.04 | Not significant |
| 3 | 1975 | 2019 | -0.03 | 0.04 | -0.11 | 0.06 | Not significant |
| 5 | 1975 | 2019 | -0.03 | 0.05 | -0.12 | 0.06 | Not significant |
| Uterus,NOS |  |  |  |  |  |  |  |
| 1 | 1975 | 2019 | 0.07 | 0.12 | -0.17 | 0.32 | Not significant |
| 3 | 1975 | 2019 | 0.07 | 0.12 | -0.17 | 0.32 | Not significant |
| 5 | 1975 | 2019 | 0.07 | 0.12 | -0.16 | 0.3 | Not significant |
| Vagina |  |  |  |  |  |  |  |
| 1 | 1975 | 2019 | -0.07 | 0.08 | -0.23 | 0.09 | Not significant |
| 3 | 1975 | 2019 | -0.1 | 0.11 | -0.31 | 0.12 | Not significant |
| 5 | 1975 | 2019 | -0.1 | 0.11 | -0.33 | 0.12 | Not significant |
| Vulva |  |  |  |  |  |  |  |
| 1 | 1975 | 2019 | -0.04 | 0.03 | -0.11 | 0.02 | Not significant |
| 3 | 1975 | 2019 | -0.07 | 0.05 | -0.18 | 0.04 | Not significant |
| 5 | 1975 | 2019 | -0.08 | 0.06 | -0.2 | 0.04 | Not significant |
| Other female genital organs |  |  |  |  |  |  |  |
| 1 | 1975 | 1988 | 0.26 | 0.53 | -0.78 | 1.3 | Not significant |
| 1 | 1988 | 1991 | 5.67 | 1.4 | 2.93 | 8.41 | Increasing |
| 1 | 1991 | 2019 | -0.31 | 0.09 | -0.49 | -0.13 | Decreasing |
| 3 | 1975 | 1988 | 0.33 | 0.68 | -1.01 | 1.67 | Not significant |
| 3 | 1988 | 1991 | 8.63 | 1.75 | 5.21 | 12.05 | Increasing |
| 3 | 1991 | 2019 | -0.51 | 0.15 | -0.81 | -0.21 | Decreasing |
| 5 | 1975 | 1988 | 0.3 | 0.63 | -0.93 | 1.53 | Not significant |
| 5 | 1988 | 1991 | 9.27 | 1.73 | 5.87 | 12.66 | Increasing |
| 5 | 1991 | 2019 | -0.58 | 0.18 | -0.92 | -0.23 | Decreasing |
| Testis |  |  |  |  |  |  |  |
| 1 | 1975 | 2019 | 0.33 | 0.14 | 0.06 | 0.6 | Increasing |
| 3 | 1975 | 2019 | 0.39 | 0.16 | 0.07 | 0.7 | Increasing |
| 5 | 1975 | 2019 | 0.43 | 0.18 | 0.07 | 0.78 | Increasing |
| Penis |  |  |  |  |  |  |  |
| 1 | 1975 | 2019 | -0.07 | 0.06 | -0.18 | 0.04 | Not significant |
| 3 | 1975 | 2019 | -0.13 | 0.1 | -0.33 | 0.07 | Not significant |
| 5 | 1975 | 2019 | -0.16 | 0.13 | -0.41 | 0.09 | Not significant |
| Other male genital organs |  |  |  |  |  |  |  |
| 1 | 1975 | 2019 | 0.26 | 0.1 | 0.06 | 0.46 | Increasing |
| 3 | 1975 | 2019 | 0.56 | 0.2 | 0.16 | 0.95 | Increasing |
| 5 | 1975 | 2019 | 0.69 | 0.24 | 0.22 | 1.16 | Increasing |
| Ureter |  |  |  |  |  |  |  |
| 1 | 1975 | 2019 | -0.27 | 0.05 | -0.36 | -0.17 | Decreasing |
| 3 | 1975 | 2019 | -0.42 | 0.08 | -0.58 | -0.27 | Decreasing |
| 5 | 1975 | 2019 | -0.46 | 0.09 | -0.63 | -0.3 | Decreasing |
| Other urinary organs |  |  |  |  |  |  |  |
| 1 | 1975 | 2014 | -0.08 | 0.1 | -0.27 | 0.12 | Not significant |
| 1 | 2014 | 2019 | -3.96 | 1.16 | -6.23 | -1.68 | Decreasing |
| 3 | 1975 | 2014 | -0.11 | 0.14 | -0.39 | 0.17 | Not significant |
| 3 | 2014 | 2019 | -4.95 | 1.37 | -7.63 | -2.27 | Decreasing |
| 5 | 1975 | 2014 | -0.12 | 0.15 | -0.42 | 0.18 | Not significant |
| 5 | 2014 | 2019 | -5.01 | 1.35 | -7.66 | -2.37 | Decreasing |
| Eye and orbit |  |  |  |  |  |  |  |
| 1 | 1975 | 2019 | -0.01 | 0.02 | -0.04 | 0.03 | Not significant |
| 3 | 1975 | 2019 | -0.02 | 0.09 | -0.19 | 0.15 | Not significant |
| 5 | 1975 | 2019 | -0.03 | 0.12 | -0.27 | 0.21 | Not significant |
| Endocrine system |  |  |  |  |  |  |  |
| 1 | 1975 | 2019 | 0.62 | 0.04 | 0.55 | 0.7 | Increasing |
| 3 | 1975 | 2019 | 0.75 | 0.04 | 0.66 | 0.83 | Increasing |
| 5 | 1975 | 2019 | 0.8 | 0.05 | 0.71 | 0.89 | Increasing |
| Hodgkin lymphoma |  |  |  |  |  |  |  |
| 1 | 1975 | 2019 | 0.66 | 0.06 | 0.54 | 0.77 | Increasing |
| 3 | 1975 | 2019 | 0.8 | 0.07 | 0.66 | 0.93 | Increasing |
| 5 | 1975 | 2019 | 0.84 | 0.07 | 0.7 | 0.98 | Increasing |
| Mesothelioma |  |  |  |  |  |  |  |
| 1 | 1975 | 2019 | 0.35 | 0.05 | 0.25 | 0.45 | Increasing |
| 3 | 1975 | 2019 | 0.2 | 0.03 | 0.15 | 0.26 | Increasing |
| 5 | 1975 | 2019 | 0.14 | 0.02 | 0.1 | 0.18 | Increasing |
| Kaposi sarcoma |  |  |  |  |  |  |  |
| 1 | 1975 | 2019 | 0.12 | 0.08 | -0.04 | 0.27 | Not significant |
| 3 | 1975 | 2019 | 0.2 | 0.13 | -0.06 | 0.46 | Not significant |
| 5 | 1975 | 2019 | 0.23 | 0.15 | -0.07 | 0.53 | Not significant |

*Numbers represent the difference in cumulative survival rate (as the percent surviving) from one year at diagnosis to the previous.
