# Supplementary material for: Trends analysis of cancer incidence, mortality, and survival for the elderly in the United States, 1975–2020
Source: Cancer Med. 2024 Jul 31;13(15):e70062. doi: 10.1002/cam4.70062 (PMC11289898; doi:10.1002/cam4.70062)
Supplement: Supplementary file 1 — Appendix S1. [file CAM4-13-e70062-s001.zip › Supplementary Table 2 Cancer types by sex.docx]

**Supplementary Table 2** Cancer types by sex.

| **Male** | **Female** |
| --- | --- |
| Prostate | Breast |
| Lung & Bronchus | Lung & Bronchus |
| Colon & Rectum | Colon & Rectum |
| Urinary Bladder | Corpus Uteri |
| Melanoma of the skin | Non-Hodgkin Lymphoma |
| Non-Hodgkin Lymphoma | Pancreas |
| Kidney & Renal Pelvis | Urinary Bladder |
| Leukemia | Melanoma of the skin |
| Pancreas | Leukemia |
| Oral cavity & Pharynx | Ovary |
| Stomach | Kidney & Renal Pelvis |
| Liver | Stomach |
| Anus, Anal Canal and Anorectum | Anus, Anal Canal and Anorectum |
| Bones and Joints | Bones and Joints |
| Brain and Other Nervous System | Brain and Other Nervous System |
| Endocrine System | Cervix Uteri |
| Esophagus | Esophagus |
| Eye and Orbit | Endocrine System |
| Gallbladder | Eye and Orbit |
| Hodgkin Lymphoma | Gallbladder |
| Intrahepatic Bile Duct | Hodgkin Lymphoma |
| Larynx | Larynx |
| Mesothelioma | Intrahepatic Bile Duct |
| Nose, Nasal Cavity and Middle Ear | Mesothelioma |
| Other Biliary | Nose, Nasal Cavity and Middle Ear |
| Other Digestive Organs | Other Biliary |
| Other Male Genital Organs | Other Digestive Organs |
| Other Non-Epithelial Skin | Other Female Genital Organs |
| Other Urinary Organs | Other Non-Epithelial Skin |
| Penis | Pleura |
| Peritoneum, Omentum and Mesentery | Retroperitoneum |
| Pleura | Small Intestine |
| Retroperitoneum | Soft Tissue including Heart |
| Small Intestine | Trachea, Mediastinum and Other Respiratory Organs |
| Soft Tissue including Heart | Ureter |
| Testis | Ureter, NOS |
| Trachea, Mediastinum and Other Respiratory Organs | Vagina |
| Ureter | Vulva |
