# Supplementary material for: Trends analysis of cancer incidence, mortality, and survival for the elderly in the United States, 1975–2020
Source: Cancer Med. 2024 Jul 31;13(15):e70062. doi: 10.1002/cam4.70062 (PMC11289898; doi:10.1002/cam4.70062)
Supplement: Supplementary file 1 — Appendix S1. [file CAM4-13-e70062-s001.zip › Supplementary Table 3 Cancer demographics of incid.docx]

**Supplementary Table 3** Cancer demographics of incidence, United States, 1975-2020^a^

| Characteristic | Age groups, N. (%) of patients | | | | | |
| --- | --- | --- | --- | --- | --- | --- |
|  | 65-69 years | 70-74 years | 75-79 years | 80-84 years | 85+ years | All |
| All | 1,412,876(100.0) | 1,340,730(100.0) | 1,158,322(100.0) | 856,360(100.0) | 731,054(100.0) | 5,499,342(100.0) |
| Sex |  |  |  |  |  |  |
| Male | 808,905(57.3) | 761,126(56.8) | 633,845(54.7) | 438,850(51.2) | 328,698(45.0) | 2,971,424(54.0) |
| Female | 603,971(42.7) | 579,604(43.2) | 524,477(45.3) | 417,510(48.8) | 402,356(55.0) | 2,527,918(46.0) |
| Race |  |  |  |  |  |  |
| White | 1,170,747(82.9) | 1,135,164(84.7) | 997,594(86.1) | 747,746(87.3) | 643,700(88.1) | 4,694,951(85.4) |
| Black | 135,200(9.6) | 107,734(8.0) | 79,987(6.9) | 51,009(6.0) | 39,450(5.4) | 413,380(7.5) |
| AIAN | 7,454(5.3) | 6,574(0.5) | 4,829(0.4) | 3,219(0.4) | 2,296(0.3) | 24,372(0.4) |
| AAPI | 86,141(6.1) | 80,381(6.0) | 68,422(5.9) | 50,029(5.8) | 42,611(5.8) | 327,584(6.0) |
| Unknown | 13,334(0.9) | 10,877(0.8) | 7,490(0.6) | 4,357(0.5) | 2,997(0.4) | 39,055(0.7) |

Abbreviation: AIAN, American Indian/Alaska Native. AAPI, Asian or Pacific Islander.

^a^ Incidence data for 1975-1999 are from the Surveillance, Epidemiology and End Results (SEER) program: Incidence - SEER Research Data, 8 Registries, Nov 2022 Sub (1975-2020) - Linked To County Attributes. Incidence data for 2000-2020 are from the SEER program: Incidence - SEER Research Data, 17 Registries, Nov 2022 Sub (2000-2020) - Linked To County Attributes.
