# Supplementary material for: Trends analysis of cancer incidence, mortality, and survival for the elderly in the United States, 1975–2020
Source: Cancer Med. 2024 Jul 31;13(15):e70062. doi: 10.1002/cam4.70062 (PMC11289898; doi:10.1002/cam4.70062)
Supplement: Supplementary file 1 — Appendix S1. [file CAM4-13-e70062-s001.zip › Supplementary Table 4 Cancer demographics of incid.docx]

**Supplementary Table 4** Cancer demographics of incidence, United States, 2000-2020^a^

| Characteristic | Age groups, N. (%) of patients | | | | | |
| --- | --- | --- | --- | --- | --- | --- |
|  | 65-69 years | 70-74 years | 75-79 years | 80-84 years | 85+ years | All |
| All | 1,140,292(100.0) | 1,060,838(100.0) | 91,9164(100.0) | 692165(100.0) | 603,966(100.0) | 4,416,425(100.0) |
| Sex |  |  |  |  |  |  |
| Male | 653,854(57.3) | 600,191(56.6) | 501,618(54.6) | 355074(51.3) | 273,562(45.3) | 2,384,299(54.0) |
| Female | 486,438(42.7) | 460,647(43.4) | 417,546(45.4) | 337091(48.7) | 330,404(54.7) | 2,032,126(46.0) |
| Race |  |  |  |  |  |  |
| White | 929,962(81.6) | 885,226(83.4) | 781,896(85.1) | 597901(86.4) | 527,089(87.3) | 3,722,074(84.3) |
| Black | 119,506(10.5) | 93,998(8.9) | 69,781(7.6) | 45071(6.5) | 35,345(5.9) | 363,701(8.2) |
| AIAN | 6,447(0.6) | 5,676(0.5) | 4,105(0.4) | 2750(0.4) | 1,909(0.3) | 20,887(0.5) |
| AAPI | 71,400(6.3) | 65,380(6.2) | 56,131(6.1) | 42269(6.1) | 36,742(6.1) | 271,922(6.2) |
| Unknown | 12,977(1.1) | 10,558(1.0) | 7,251(0.7) | 4174(0.6) | 2,881(0.5) | 37,841(0.9) |

Abbreviation: AIAN, American Indian/Alaska Native. AAPI, Asian or Pacific Islander.

^a^ Incidence data for 2000-2020 are from the SEER program: Incidence - SEER Research Data, 17 Registries, Nov 2022 Sub (2000-2020) - Linked To County Attributes.
