# Supplementary material for: Trends analysis of cancer incidence, mortality, and survival for the elderly in the United States, 1975–2020
Source: Cancer Med. 2024 Jul 31;13(15):e70062. doi: 10.1002/cam4.70062 (PMC11289898; doi:10.1002/cam4.70062)
Supplement: Supplementary file 1 — Appendix S1. [file CAM4-13-e70062-s001.zip › Supplementary Table 5 Cancer demographics of morta.docx]

**Supplementary Table 5** Cancer demographics of mortality, United States, 1975-2020^a^

| Characteristic | Age groups, N. (%) of patients | | | | | |
| --- | --- | --- | --- | --- | --- | --- |
|  | 65-69 years | 70-74 years | 75-79 years | 80-84 years | 85+ years | All |
| All | 3,219,272(100.0) | 3,549,476(100.0) | 3,467,416(100.0) | 2,942,280(100.0) | 3,157,389(100.0) | 16,335,833(100.0) |
| Gender |  |  |  |  |  |  |
| Male | 1,814,294(56.4) | 1,979,018(55.8) | 1,878,131(54.2) | 1,515,650(51.5) | 1,427,714(45.2) | 8,614,807(52.7) |
| Female | 1,404,978(43.6) | 1,570,458(44.2) | 1,589,285(45.8) | 1,426,630(48.5) | 1,729,675(54.8) | 7,721,026(47.3) |
| Race |  |  |  |  |  |  |
| White | 2,773,161(86.1) | 3,110,524(87.6) | 3,080,095(88.8) | 2,642,624(89.8) | 2,854,715(90.4) | 14,461,119(88.5) |
| Black | 381,924(11.9) | 371,738(10.5) | 322,846(9.3) | 245,415(8.3) | 244,028(7.7) | 1,565,951(9.6) |
| Other^b^ | 64,137(2.0) | 67,164(1.9) | 64,443(1.9) | 54,206(1.8) | 58,617(1.9) | 308,567(1.9) |

^a^ Mortality data are from the Surveillance, Epidemiology, and End Results (SEER) database: Mortality - All COD, Aggregated Total U.S. (1969-2020) <Katrina/Rita Population Adjustment>, National Cancer Institute, DCCPS, Surveillance Research Program, released June 2022.

^b^ Other: American Indian/Alaska Native, Asian or Pacific Islander.
