# Supplementary material for: Trends analysis of cancer incidence, mortality, and survival for the elderly in the United States, 1975–2020
Source: Cancer Med. 2024 Jul 31;13(15):e70062. doi: 10.1002/cam4.70062 (PMC11289898; doi:10.1002/cam4.70062)
Supplement: Supplementary file 1 — Appendix S1. [file CAM4-13-e70062-s001.zip › Supplementary Table 6 Cancer demographics of morta.docx]

**Supplementary Table 6** Cancer demographics of mortality, United States, 2000-2020^a^

| Characteristic | Age groups, N. (%) of patients | | | | | |
| --- | --- | --- | --- | --- | --- | --- |
|  | 65-69 years | 70-74 years | 75-79 years | 80-84 years | 85+ years | All |
| All | 1,507,580(100.0) | 1,682,465(100.0) | 1,749,404(100.0) | 1,615,029(100.0) | 1,938,437(100.0) | 8,492,915(100.0) |
| Gender |  |  |  |  |  |  |
| Male | 836,887(55.5) | 923,175(54.9) | 939,532(53.7) | 836,578(51.8) | 893,960(46.1) | 4,430,132(52.2) |
| Female | 670,693(44.5) | 759,290(45.1) | 809,872(46.3) | 778,451(48.2) | 1,044,477(53.9) | 4,062,783(47.8) |
| Race |  |  |  |  |  |  |
| White | 1,271,993(84.4) | 1,451,909(86.3) | 1,535,648(87.8) | 1,437,192(89.0) | 1,743,570(90.0) | 7,440,312(87.6) |
| Black | 191,016(12.7) | 183,667(10.9) | 167,249(9.6) | 136,696(8.5) | 147,809(7.6) | 826,437(9.7) |
| Other^b^ | 44,571(3.0) | 46,889(2.8) | 46,507(2.7) | 41,141(2.5) | 47,058(2.4) | 226,166(2.7) |

^a^ Mortality data are from the Surveillance, Epidemiology, and End Results (SEER) database: Mortality - All COD, Aggregated Total U.S. (1969-2020) <Katrina/Rita Population Adjustment>, National Cancer Institute, DCCPS, Surveillance Research Program, released June 2022.

^b^ Other: American Indian/Alaska Native, Asian or Pacific Islander.
