# Supplementary material for: Trends analysis of cancer incidence, mortality, and survival for the elderly in the United States, 1975–2020
Source: Cancer Med. 2024 Jul 31;13(15):e70062. doi: 10.1002/cam4.70062 (PMC11289898; doi:10.1002/cam4.70062)
Supplement: Supplementary file 1 — Appendix S1. [file CAM4-13-e70062-s001.zip › Supplementary Table 7 Cancer mortality distributio.docx]

**Supplementary Table 7** Cancer mortality distribution by sex and age, United States, 1975-2020^a^

| **Cancer subtype^b^** | **Age group, No. (%)** | | | | | |
| --- | --- | --- | --- | --- | --- | --- |
|  | **65-69 years** | **70-74 years** | **75-79 years** | **80-84 years** | **85+ years** | **All** |
| **Male** |  |  |  |  |  |  |
| **All Sites** | 1,814,294(21.1) | 1,979,018(23.0) | 1,878,131(21.8) | 1,515,650(17.6) | 1,427,714(16.6) | 8,614,807(100.0) |
| Lung and Bronchus | 659,432(25.3) | 687,890(26.4) | 588,596(22.6) | 400,461(15.4) | 269,768(10.4) | 2,606,147(100.0) |
| Prostate | 135,148(11.3) | 200,997(16.9) | 251,700(21.1) | 263,092(22.1) | 341,106(28.6) | 1,192,043(100.0) |
| Colon and Rectum | 174,849(19.9) | 193,872(22.1) | 189,218(21.6) | 159,356(18.2) | 159,875(18.2) | 877,170(100.0) |
| Pancreas | 107,228(23.0) | 112,446(24.1) | 102,687(22) | 78,711(16.9) | 65,308(14.0) | 466,380(100.0) |
| Leukemia | 59,586(16.9) | 74,196(21.1) | 79,205(22.5) | 70,083(19.9) | 69,104(19.6) | 352,174(100.0) |
| Urinary Bladder | 44,737(13.7) | 58,867(18.0) | 67,995(20.8) | 68,570(21.0) | 86,179(26.4) | 326,348(100.0) |
| Non-Hodgkin Lymphoma | 55,979(18.4) | 65,834(21.7) | 68,049(22.4) | 59,034(19.4) | 54,760(18.0) | 303,656(100.0) |
| Esophagus | 66,929(27.2) | 63,180(25.7) | 51,595(21.0) | 36,221(14.7) | 27,704(11.3) | 245,629(100.0) |
| Stomach | 48,557(20.7) | 53,563(22.9) | 51,767(22.1) | 41,563(17.8) | 38,662(16.5) | 234,112(100.0) |
| Kidney and Renal Pelvis | 46,422(23.5) | 47,023(23.8) | 42,297(21.4) | 32,551(16.4) | 29,610(15.0) | 197,903(100.0) |
| Liver | 52,860(27.6) | 47,283(24.7) | 40,127(21.0) | 29,222(15.3) | 21,852(11.4) | 191,344(100.0) |
| Myeloma | 33,138(19.3) | 39,041(22.7) | 39,155(22.8) | 32,705(19.0) | 27,974(16.3) | 172,013(100.0) |
| **Female** |  |  |  |  |  |  |
| **All Sites** | 1,404,978(18.2) | 1,570,458(20.3) | 1,589,285(20.6) | 1,426,630(18.5) | 1,729,675(22.4) | 7,721,026(100.0) |
| Lung and Bronchus | 381,366(22.0) | 419,032(24.1) | 387,623(22.3) | 294,363(16.9) | 255,017(14.7) | 1,737,401(100.0) |
| Breast | 219,351(20.8) | 216,996(20.6) | 203,862(19.3) | 178,218(16.9) | 236,849(22.4) | 1,055,276(100.0) |
| Colon and Rectum | 133,532(14.0) | 162,822(17.0) | 185,372(19.4) | 190,664(19.9) | 284,128(29.7) | 956,518(100.0) |
| Pancreas | 86,314(16.3) | 1,045,97(19.7) | 112,098(21.1) | 104,034(19.6) | 123,287(23.2) | 530,330(100.0) |
| Ovary | 82,231(21.5) | 85,296(22.4) | 81,180(21.3) | 67,633(17.7) | 65,270(17.1) | 381,610(100.0) |
| Non-Hodgkin Lymphoma | 42,922(14.0) | 55,782(18.1) | 65,331(21.3) | 65,465(21.3) | 77,855(25.3) | 307,355(100.0) |
| Leukemia | 37,145(13.0) | 48,788(17.0) | 57,535(20.1) | 59,354(20.7) | 83,656(29.2) | 286,478(100.0) |
| Stomach | 24,913(14.0) | 31,071(17.5) | 35,818(20.1) | 36,341(20.4) | 49,794(28.0) | 177,937(100.0) |
| Myeloma | 26,390(15.9) | 33,143(20.0) | 36,518(22.0) | 34,099(20.6) | 35,643(21.5) | 165,793(100.0) |
| Urinary Bladder | 15,125(10.1) | 21,609(14.4) | 27,440(18.3) | 31,852(21.3) | 53,641(35.8) | 149,667(100.0) |
| Kidney and Renal Pelvis | 23,426(17.3) | 26,955(20.0) | 27.678(20.5) | 25,666(19.0) | 31,329(23.2) | 135,054(100.0) |
| Brain and Other Nervous System | 33,346(24.8) | 33,202(24.7) | 28.828(21.4) | 21,252(15.8) | 17,905(13.3) | 134,533(100.0) |

^a^ Mortality data are from the Surveillance, Epidemiology, and End Results (SEER) database: Mortality - All COD, Aggregated Total U.S. (1969-2020) <Katrina/Rita Population Adjustment>, National Cancer Institute, DCCPS, Surveillance Research Program, released June 2022.

^b^ Ranked by decreasing incidence for males and females. The 12 sites listed respectively for male and female are those with the highest incidence (1975-2020).
