# Supplementary material for: Trends analysis of cancer incidence, mortality, and survival for the elderly in the United States, 1975–2020
Source: Cancer Med. 2024 Jul 31;13(15):e70062. doi: 10.1002/cam4.70062 (PMC11289898; doi:10.1002/cam4.70062)
Supplement: Supplementary file 1 — Appendix S1. [file CAM4-13-e70062-s001.zip › Supplementary Table 8 All cancer incidence distrib.docx]

**Supplementary Table 8** All cancer incidence distribution by sex and age, United States, 1975-2020^a^

|  | 65-69 years | 70-74 years | 75-79 years | 80-84 years | 85+ years | Total |
| --- | --- | --- | --- | --- | --- | --- |
| Male and female |  |  |  |  |  |  |
| All Sites | 1,412,876 | 1,340,730 | 1,158,322 | 856,360 | 731,054 | 5,499,342 |
| Oral Cavity and Pharynx | 34,811 | 28,901 | 22,245 | 15,946 | 14,221 | 116,124 |
| Lip | 2,726 | 2,732 | 2,525 | 2,080 | 2,100 | 12,163 |
| Tongue | 10,144 | 8,209 | 5,876 | 3,789 | 3,065 | 31,083 |
| Salivary Gland | 3,067 | 3,000 | 2,802 | 2,458 | 2,711 | 14,038 |
| Floor of Mouth | 2,270 | 1,771 | 1,287 | 841 | 576 | 6,745 |
| Gum and Other Mouth | 4,633 | 4,580 | 4,070 | 3,461 | 3,624 | 20,368 |
| Nasopharynx | 1,512 | 1,106 | 738 | 480 | 313 | 4,149 |
| Tonsil | 5,426 | 3,375 | 2,032 | 1,080 | 615 | 12,528 |
| Oropharynx | 1,520 | 1,167 | 745 | 472 | 336 | 4,240 |
| Hypopharynx | 2,649 | 2,245 | 1,666 | 944 | 595 | 8,099 |
| Other Oral Cavity and Pharynx | 864 | 716 | 504 | 341 | 286 | 2,711 |
| Digestive System | 252,258 | 254,091 | 241,216 | 200,806 | 192,738 | 1,141,109 |
| Esophagus | 15,268 | 14,110 | 11,909 | 8,735 | 7,286 | 57,308 |
| Stomach | 22,016 | 23,474 | 22,776 | 19,232 | 18,745 | 106,243 |
| Small Intestine | 6,185 | 5,864 | 5,054 | 3,776 | 3,059 | 23,938 |
| Colon and Rectum | 129,694 | 133,845 | 130,612 | 112,071 | 110,679 | 616,901 |
| Colon excluding Rectum | 90,769 | 98,240 | 99,273 | 87,815 | 89,022 | 465,119 |
| Cecum | 18,960 | 22,125 | 24,310 | 22,704 | 23,874 | 111,973 |
| Appendix | 1,987 | 1,624 | 1,264 | 790 | 607 | 6,272 |
| Ascending Colon | 15,468 | 18,359 | 19,198 | 17,914 | 18,582 | 89,521 |
| Hepatic Flexure | 4,233 | 5,088 | 5,416 | 4,955 | 5,149 | 24,841 |
| Transverse Colon | 8,337 | 9,660 | 9,929 | 9,251 | 9,505 | 46,682 |
| Splenic Flexure | 3,199 | 3,279 | 3,291 | 2,891 | 2,657 | 15,317 |
| Descending Colon | 5,955 | 6,171 | 5,718 | 4,576 | 4,253 | 26,673 |
| Sigmoid Colon | 28,699 | 27,645 | 25,453 | 19,656 | 16,875 | 118,328 |
| Large Intestine, NOS | 3,931 | 4,289 | 4,694 | 5,078 | 7,520 | 25,512 |
| Rectum and Rectosigmoid Junction | 38,925 | 35,605 | 31,339 | 24,256 | 21,657 | 151,782 |
| Rectosigmoid Junction | 10,905 | 10,330 | 9,295 | 7,007 | 5,912 | 43,449 |
| Rectum | 28,020 | 25,275 | 22,044 | 17,249 | 15,745 | 108,333 |
| Anus, Anal Canal and Anorectum | 4,604 | 3,816 | 3,018 | 2,386 | 2,272 | 16,096 |
| Liver and Intrahepatic Bile Duct | 24,440 | 20,120 | 16,699 | 11,819 | 9,139 | 82,217 |
| Liver | 21,402 | 17,199 | 14,035 | 9,736 | 7,185 | 69,557 |
| Intrahepatic Bile Duct | 3,038 | 2,921 | 2,664 | 2,083 | 1,954 | 12,660 |
| Gallbladder | 3,489 | 3,905 | 4,033 | 3,458 | 3,516 | 18,401 |
| Other Biliary | 5,032 | 5,521 | 5,601 | 5,074 | 5,120 | 26,348 |
| Pancreas | 37,171 | 38,995 | 37,395 | 31,137 | 30,064 | 174,762 |
| Retroperitoneum | 1,016 | 965 | 810 | 559 | 394 | 3,744 |
| Peritoneum, Omentum and Mesentery | 1,797 | 1,762 | 1,516 | 980 | 656 | 6,711 |
| Other Digestive Organs | 1,546 | 1,714 | 1,793 | 1,579 | 1,808 | 8,440 |
| Respiratory System | 220,117 | 228,503 | 201,756 | 139,780 | 96,189 | 886,345 |
| Nose, Nasal Cavity and Middle Ear | 1,851 | 1,722 | 1,560 | 1,246 | 1,157 | 7,536 |
| Larynx | 12,859 | 10,853 | 7,724 | 4,847 | 3,230 | 39,513 |
| Lung and Bronchus | 205,010 | 215,544 | 192,121 | 133,420 | 91,570 | 837,665 |
| Pleura | 77 | 78 | 91 | 86 | 81 | 413 |
| Trachea, Mediastinum and Other Respiratory Organs | 320 | 306 | 260 | 181 | 151 | 1,218 |
| Bones and Joints | 1,174 | 1,091 | 829 | 719 | 622 | 4,435 |
| Soft Tissue including Heart | 6,407 | 6,206 | 5,743 | 4,835 | 4,791 | 27,982 |
| Skin excluding Basal and Squamous | 54,994 | 52,278 | 46,337 | 37,666 | 36,949 | 228,224 |
| Melanoma of the Skin | 51,174 | 47,842 | 41,393 | 32,768 | 30,901 | 204,078 |
| Other Non-Epithelial Skin | 3,820 | 4,436 | 4,944 | 4,898 | 6,048 | 24,146 |
| Breast | 186,628 | 162,183 | 131,171 | 93,075 | 79,748 | 652,805 |
| Female Genital System | 75,850 | 62,582 | 48,714 | 35,142 | 30,729 | 253,017 |
| Cervix Uteri | 6,152 | 4,660 | 3,525 | 2,564 | 2,353 | 19,254 |
| Corpus and Uterus, NOS | 46,348 | 35,936 | 25,563 | 16,639 | 12,589 | 137,075 |
| Corpus Uteri | 45,417 | 35,092 | 24,860 | 16,014 | 11,711 | 133,094 |
| Uterus, NOS | 931 | 844 | 703 | 625 | 878 | 3,981 |
| Ovary | 17,311 | 15,962 | 13,857 | 10,881 | 9,822 | 67,833 |
| Vagina | 1,054 | 995 | 1,020 | 872 | 1,017 | 4,958 |
| Vulva | 2,993 | 3,160 | 3,337 | 3,161 | 4,041 | 16,692 |
| Other Female Genital Organs | 1,992 | 1,869 | 1,412 | 1,025 | 907 | 7,205 |
| Male Genital System | 286,983 | 245,936 | 175,817 | 97,262 | 61,290 | 867,288 |
| Prostate | 284,947 | 244,128 | 174,236 | 96,034 | 60,095 | 859,440 |
| Testis | 584 | 336 | 218 | 142 | 97 | 1,377 |
| Penis | 1,116 | 1,105 | 1,041 | 821 | 868 | 4,951 |
| Other Male Genital Organs | 336 | 367 | 322 | 265 | 230 | 1,520 |
| Urinary System | 109,061 | 112,230 | 105,501 | 83,315 | 73,495 | 483,602 |
| Urinary Bladder | 61,358 | 68,984 | 69,591 | 58,465 | 54,670 | 313,068 |
| Kidney and Renal Pelvis | 45,018 | 40,050 | 32,513 | 21,837 | 16,140 | 155,558 |
| Ureter | 1,746 | 2,096 | 2,275 | 1,964 | 1,576 | 9,657 |
| Other Urinary Organs | 939 | 1,100 | 1,122 | 1,049 | 1,109 | 5,319 |
| Eye and Orbit | 2,280 | 1,905 | 1,628 | 1,254 | 1,004 | 8,071 |
| Brain and Other Nervous System | 13,753 | 12,702 | 10,475 | 7,257 | 5,231 | 49,418 |
| Brain | 13,202 | 12,200 | 10,078 | 6,984 | 5,003 | 47,467 |
| Cranial Nerves Other Nervous System | 551 | 502 | 397 | 273 | 228 | 1,951 |
| Endocrine System | 19,495 | 14,097 | 9,456 | 5,436 | 3,440 | 51,924 |
| Thyroid | 17,949 | 12,873 | 8,559 | 4,824 | 3,067 | 47,272 |
| Other Endocrine including Thymus | 1,546 | 1,224 | 897 | 612 | 373 | 4,652 |
| Lymphoma | 53,732 | 54,205 | 51,901 | 41,193 | 35,732 | 236,763 |
| Hodgkin Lymphoma | 2,817 | 2,568 | 2,197 | 1,541 | 1,096 | 10,219 |
| Hodgkin - Nodal | 2,731 | 2,505 | 2,110 | 1,475 | 1,035 | 9,856 |
| Hodgkin - Extranodal | 86 | 63 | 87 | 66 | 61 | 363 |
| Non-Hodgkin Lymphoma | 50,915 | 51,637 | 49,704 | 39,652 | 34,636 | 226,544 |
| NHL - Nodal | 34,576 | 34,999 | 33,632 | 26,573 | 22,824 | 152,604 |
| NHL - Extranodal | 16,339 | 16,638 | 16,072 | 13,079 | 11,812 | 73,940 |
| Myeloma | 20,198 | 20,593 | 19,072 | 14,878 | 11,398 | 86,139 |
| Leukemia | 32,976 | 34,728 | 34,040 | 28,509 | 27,814 | 158,067 |
| Lymphocytic Leukemia | 17,326 | 17,540 | 16,564 | 13,262 | 13,095 | 77,787 |
| Acute Lymphocytic Leukemia | 1,122 | 980 | 830 | 622 | 552 | 4,106 |
| Chronic Lymphocytic Leukemia | 15,172 | 15,611 | 14,853 | 11,912 | 11,805 | 69,353 |
| Other Lymphocytic Leukemia | 1,032 | 949 | 881 | 728 | 738 | 4,328 |
| Myeloid and Monocytic Leukemia | 14,100 | 15,473 | 15,520 | 13,302 | 12,158 | 70,553 |
| Acute Myeloid Leukemia | 9,197 | 10,389 | 10,256 | 8,697 | 7,745 | 46,284 |
| Acute Monocytic Leukemia | 500 | 513 | 567 | 477 | 444 | 2,501 |
| Chronic Myeloid Leukemia | 4,013 | 4,080 | 4,223 | 3,650 | 3,461 | 19,427 |
| Other Myeloid/Monocytic Leukemia | 390 | 491 | 474 | 478 | 508 | 2,341 |
| Other Leukemia | 1,550 | 1,715 | 1,956 | 1,945 | 2,561 | 9,727 |
| Other Acute Leukemia | 575 | 726 | 859 | 868 | 1,152 | 4,180 |
| Aleukemic, Subleukemic and NOS | 975 | 989 | 1,097 | 1,077 | 1,409 | 5,547 |
| Mesothelioma | 2,726 | 3,353 | 3,642 | 2,971 | 2,376 | 15,068 |
| Kaposi Sarcoma | 458 | 483 | 540 | 542 | 664 | 2,687 |
| Miscellaneous | 38,975 | 44,663 | 48,239 | 45,774 | 52,623 | 230,274 |
| Male |  |  |  |  |  |  |
| All Sites | 808,905 | 761,126 | 633,845 | 438,850 | 328,698 | 2,971,424 |
| Oral Cavity and Pharynx | 25,092 | 19,889 | 14,214 | 9,547 | 7,228 | 75,970 |
| Lip | 2,208 | 2,187 | 1,917 | 1,500 | 1,330 | 9,142 |
| Tongue | 7,480 | 5,661 | 3,713 | 2,181 | 1,377 | 20,412 |
| Salivary Gland | 1,886 | 1,896 | 1,783 | 1,642 | 1,682 | 8,889 |
| Floor of Mouth | 1,551 | 1,139 | 743 | 441 | 261 | 4,135 |
| Gum and Other Mouth | 2,747 | 2,510 | 1,949 | 1,512 | 1,275 | 9,993 |
| Nasopharynx | 1,063 | 768 | 487 | 281 | 169 | 2,768 |
| Tonsil | 4,270 | 2,557 | 1,455 | 743 | 368 | 9,393 |
| Oropharynx | 1,182 | 890 | 540 | 316 | 180 | 3,108 |
| Hypopharynx | 2,082 | 1,786 | 1,279 | 706 | 425 | 6,278 |
| Other Oral Cavity and Pharynx | 623 | 495 | 348 | 225 | 161 | 1,852 |
| Digestive System | 147,762 | 142,106 | 125,215 | 95,018 | 75,611 | 585,712 |
| Esophagus | 12,143 | 10,847 | 8,763 | 6,042 | 4,379 | 42,174 |
| Stomach | 14,533 | 15,072 | 13,855 | 10,855 | 9,013 | 63,328 |
| Small Intestine | 3,363 | 3,196 | 2,669 | 1,848 | 1,321 | 12,397 |
| Colon and Rectum | 72,770 | 72,138 | 64,873 | 50,262 | 40,942 | 300,985 |
| Colon excluding Rectum | 49,074 | 50,927 | 47,506 | 37,907 | 31,811 | 217,225 |
| Cecum | 9,166 | 10,232 | 10,294 | 8,839 | 7,819 | 46,350 |
| Appendix | 918 | 784 | 613 | 365 | 264 | 2,944 |
| Ascending Colon | 7,713 | 8,848 | 8,575 | 7,188 | 6,357 | 38,681 |
| Hepatic Flexure | 2,257 | 2,650 | 2,671 | 2,225 | 1,949 | 11,752 |
| Transverse Colon | 4,396 | 4,959 | 4,592 | 3,877 | 3,208 | 21,032 |
| Splenic Flexure | 1,910 | 1,859 | 1,778 | 1,417 | 1,088 | 8,052 |
| Descending Colon | 3,464 | 3,503 | 3,053 | 2,230 | 1,767 | 14,017 |
| Sigmoid Colon | 17,021 | 15,818 | 13,669 | 9,618 | 6,927 | 63,053 |
| Large Intestine, NOS | 2,229 | 2,274 | 2,261 | 2,148 | 2,432 | 11,344 |
| Rectum and Rectosigmoid Junction | 23,696 | 21,211 | 17,367 | 12,355 | 9,131 | 83,760 |
| Rectosigmoid Junction | 6,545 | 6,030 | 5,047 | 3,408 | 2,358 | 23,388 |
| Rectum | 17,151 | 15,181 | 12,320 | 8,947 | 6,773 | 60,372 |
| Anus, Anal Canal and Anorectum | 1,589 | 1,339 | 1,041 | 802 | 647 | 5,418 |
| Liver and Intrahepatic Bile Duct | 17,672 | 13,461 | 10,384 | 6,859 | 4,572 | 52,948 |
| Liver | 16,034 | 11,929 | 9,106 | 5,870 | 3,771 | 46,710 |
| Intrahepatic Bile Duct | 1,638 | 1,532 | 1,278 | 989 | 801 | 6,238 |
| Gallbladder | 1,146 | 1,278 | 1,312 | 1,005 | 872 | 5,613 |
| Other Biliary | 2,833 | 3,055 | 2,995 | 2,567 | 2,144 | 13,594 |
| Pancreas | 20,222 | 20,244 | 17,891 | 13,724 | 10,760 | 82,841 |
| Retroperitoneum | 543 | 469 | 416 | 276 | 184 | 1,888 |
| Peritoneum, Omentum and Mesentery | 120 | 99 | 90 | 69 | 56 | 434 |
| Other Digestive Organs | 828 | 908 | 926 | 709 | 721 | 4,092 |
| Respiratory System | 128,055 | 129,891 | 112,235 | 75,614 | 48,156 | 493,951 |
| Nose, Nasal Cavity and Middle Ear | 1,172 | 1,056 | 871 | 693 | 500 | 4,292 |
| Larynx | 10,463 | 8,863 | 6,360 | 4,001 | 2,605 | 32,292 |
| Lung and Bronchus | 116,166 | 119,728 | 104,798 | 70,770 | 44,915 | 456,377 |
| Pleura | 45 | 53 | 62 | 57 | 53 | 270 |
| Trachea, Mediastinum and Other Respiratory Organs | 209 | 191 | 144 | 93 | 83 | 720 |
| Bones and Joints | 667 | 621 | 464 | 361 | 252 | 2,365 |
| Soft Tissue including Heart | 3,627 | 3,573 | 3,274 | 2,700 | 2,491 | 15,665 |
| Skin excluding Basal and Squamous | 35,642 | 34,868 | 31,087 | 24,740 | 22,274 | 148,611 |
| Melanoma of the Skin | 33,177 | 32,001 | 27,879 | 21,582 | 18,749 | 133,388 |
| Other Non-Epithelial Skin | 2,465 | 2,867 | 3,208 | 3,158 | 3,525 | 15,223 |
| Breast | 1,621 | 1,577 | 1,319 | 1,073 | 889 | 6,479 |
| Female Genital System | 0 | 0 | 0 | 0 | 0 | 0 |
| Cervix Uteri | 0 | 0 | 0 | 0 | 0 | 0 |
| Corpus and Uterus, NOS | 0 | 0 | 0 | 0 | 0 | 0 |
| Corpus Uteri | 0 | 0 | 0 | 0 | 0 | 0 |
| Uterus, NOS | 0 | 0 | 0 | 0 | 0 | 0 |
| Ovary | 0 | 0 | 0 | 0 | 0 | 0 |
| Vagina | 0 | 0 | 0 | 0 | 0 | 0 |
| Vulva | 0 | 0 | 0 | 0 | 0 | 0 |
| Other Female Genital Organs | 0 | 0 | 0 | 0 | 0 | 0 |
| Male Genital System | 286,983 | 245,936 | 175,817 | 97,262 | 61,290 | 867,288 |
| Prostate | 284,947 | 244,128 | 174,236 | 96,034 | 60,095 | 859,440 |
| Testis | 584 | 336 | 218 | 142 | 97 | 1,377 |
| Penis | 1,116 | 1,105 | 1,041 | 821 | 868 | 4,951 |
| Other Male Genital Organs | 336 | 367 | 322 | 265 | 230 | 1,520 |
| Urinary System | 78,679 | 81,043 | 75,254 | 57,918 | 47,627 | 340,521 |
| Urinary Bladder | 47,688 | 53,531 | 53,298 | 43,478 | 37,884 | 235,879 |
| Kidney and Renal Pelvis | 29,202 | 25,411 | 19,706 | 12,570 | 8,226 | 95,115 |
| Ureter | 1,149 | 1,342 | 1,430 | 1,136 | 788 | 5,845 |
| Other Urinary Organs | 640 | 759 | 820 | 734 | 729 | 3,682 |
| Eye and Orbit | 1,260 | 1,057 | 929 | 669 | 466 | 4,381 |
| Brain and Other Nervous System | 7,793 | 6,972 | 5,530 | 3,682 | 2,323 | 26,300 |
| Brain | 7,532 | 6,719 | 5,357 | 3,549 | 2,237 | 25,394 |
| Cranial Nerves Other Nervous System | 261 | 253 | 173 | 133 | 86 | 906 |
| Endocrine System | 6,657 | 4,963 | 3,367 | 1,962 | 1,071 | 18,020 |
| Thyroid | 5,869 | 4,358 | 2,913 | 1,656 | 900 | 15,696 |
| Other Endocrine including Thymus | 788 | 605 | 454 | 306 | 171 | 2,324 |
| Lymphoma | 29,486 | 28,991 | 26,852 | 20,276 | 15,897 | 121,502 |
| Hodgkin Lymphoma | 1,645 | 1,437 | 1,138 | 773 | 477 | 5,470 |
| Hodgkin - Nodal | 1,601 | 1,414 | 1,086 | 734 | 442 | 5,277 |
| Hodgkin - Extranodal | 44 | 23 | 52 | 39 | 35 | 193 |
| Non-Hodgkin Lymphoma | 27,841 | 27,554 | 25,714 | 19,503 | 15,420 | 116,032 |
| NHL - Nodal | 19,000 | 18,748 | 17,283 | 13,028 | 10,081 | 78,140 |
| NHL - Extranodal | 8,841 | 8,806 | 8,431 | 6,475 | 5,339 | 37,892 |
| Myeloma | 11,501 | 11,611 | 10,273 | 7,718 | 5,509 | 46,612 |
| Leukemia | 19,987 | 20,927 | 19,795 | 15,640 | 13,227 | 89,576 |
| Lymphocytic Leukemia | 10,848 | 10,787 | 9,690 | 7,294 | 6,321 | 44,940 |
| Acute Lymphocytic Leukemia | 563 | 544 | 410 | 301 | 231 | 2,049 |
| Chronic Lymphocytic Leukemia | 9,490 | 9,561 | 8,686 | 6,539 | 5,709 | 39,985 |
| Other Lymphocytic Leukemia | 795 | 682 | 594 | 454 | 381 | 2,906 |
| Myeloid and Monocytic Leukemia | 8,281 | 9,168 | 9,001 | 7,325 | 5,814 | 39,589 |
| Acute Myeloid Leukemia | 5,321 | 6,068 | 5,907 | 4,761 | 3,655 | 25,712 |
| Acute Monocytic Leukemia | 275 | 297 | 336 | 286 | 243 | 1,437 |
| Chronic Myeloid Leukemia | 2,469 | 2,500 | 2,485 | 2,010 | 1,666 | 11,130 |
| Other Myeloid/Monocytic Leukemia | 216 | 303 | 273 | 268 | 250 | 1,310 |
| Other Leukemia | 858 | 972 | 1,104 | 1,021 | 1,092 | 5,047 |
| Other Acute Leukemia | 329 | 431 | 481 | 464 | 450 | 2,155 |
| Aleukemic, Subleukemic and NOS | 529 | 541 | 623 | 557 | 642 | 2,892 |
| Mesothelioma | 2,157 | 2,685 | 2,871 | 2,349 | 1,830 | 11,892 |
| Kaposi Sarcoma | 400 | 381 | 401 | 343 | 386 | 1,911 |
| Miscellaneous | 21,536 | 24,035 | 24,948 | 21,978 | 22,171 | 114,668 |
| Female |  |  |  |  |  |  |
| All Sites | 603,971 | 579,604 | 524,477 | 417,510 | 402,356 | 2,527,918 |
| Oral Cavity and Pharynx | 9,719 | 9,012 | 8,031 | 6,399 | 6,993 | 40,154 |
| Lip | 518 | 545 | 608 | 580 | 770 | 3,021 |
| Tongue | 2,664 | 2,548 | 2,163 | 1,608 | 1,688 | 10,671 |
| Salivary Gland | 1,181 | 1,104 | 1,019 | 816 | 1,029 | 5,149 |
| Floor of Mouth | 719 | 632 | 544 | 400 | 315 | 2,610 |
| Gum and Other Mouth | 1,886 | 2,070 | 2,121 | 1,949 | 2,349 | 10,375 |
| Nasopharynx | 449 | 338 | 251 | 199 | 144 | 1,381 |
| Tonsil | 1,156 | 818 | 577 | 337 | 247 | 3,135 |
| Oropharynx | 338 | 277 | 205 | 156 | 156 | 1,132 |
| Hypopharynx | 567 | 459 | 387 | 238 | 170 | 1,821 |
| Other Oral Cavity and Pharynx | 241 | 221 | 156 | 116 | 125 | 859 |
| Digestive System | 104,496 | 111,985 | 116,001 | 105,788 | 117,127 | 555,397 |
| Esophagus | 3,125 | 3,263 | 3,146 | 2,693 | 2,907 | 15,134 |
| Stomach | 7,483 | 8,402 | 8,921 | 8,377 | 9,732 | 42,915 |
| Small Intestine | 2,822 | 2,668 | 2,385 | 1,928 | 1,738 | 11,541 |
| Colon and Rectum | 56,924 | 61,707 | 65,739 | 61,809 | 69,737 | 315,916 |
| Colon excluding Rectum | 41,695 | 47,313 | 51,767 | 49,908 | 57,211 | 247,894 |
| Cecum | 9,794 | 11,893 | 14,016 | 13,865 | 16,055 | 65,623 |
| Appendix | 1,069 | 840 | 651 | 425 | 343 | 3,328 |
| Ascending Colon | 7,755 | 9,511 | 10,623 | 10,726 | 12,225 | 50,840 |
| Hepatic Flexure | 1,976 | 2,438 | 2,745 | 2,730 | 3,200 | 13,089 |
| Transverse Colon | 3,941 | 4,701 | 5,337 | 5,374 | 6,297 | 25,650 |
| Splenic Flexure | 1,289 | 1,420 | 1,513 | 1,474 | 1,569 | 7,265 |
| Descending Colon | 2,491 | 2,668 | 2,665 | 2,346 | 2,486 | 12,656 |
| Sigmoid Colon | 11,678 | 11,827 | 11,784 | 10,038 | 9,948 | 55,275 |
| Large Intestine, NOS | 1,702 | 2,015 | 2,433 | 2,930 | 5,088 | 14,168 |
| Rectum and Rectosigmoid Junction | 15,229 | 14,394 | 13,972 | 11,901 | 12,526 | 68,022 |
| Rectosigmoid Junction | 4,360 | 4,300 | 4,248 | 3,599 | 3,554 | 20,061 |
| Rectum | 10,869 | 10,094 | 9,724 | 8,302 | 8,972 | 47,961 |
| Anus, Anal Canal and Anorectum | 3,015 | 2,477 | 1,977 | 1,584 | 1,625 | 10,678 |
| Liver and Intrahepatic Bile Duct | 6,768 | 6,659 | 6,315 | 4,960 | 4,567 | 29,269 |
| Liver | 5,368 | 5,270 | 4,929 | 3,866 | 3,414 | 22,847 |
| Intrahepatic Bile Duct | 1,400 | 1,389 | 1,386 | 1,094 | 1,153 | 6,422 |
| Gallbladder | 2,343 | 2,627 | 2,721 | 2,453 | 2,644 | 12,788 |
| Other Biliary | 2,199 | 2,466 | 2,606 | 2,507 | 2,976 | 12,754 |
| Pancreas | 16,949 | 18,751 | 19,504 | 17,413 | 19,304 | 91,921 |
| Retroperitoneum | 473 | 496 | 394 | 283 | 210 | 1,856 |
| Peritoneum, Omentum and Mesentery | 1,677 | 1,663 | 1,426 | 911 | 600 | 6,277 |
| Other Digestive Organs | 718 | 806 | 867 | 870 | 1,087 | 4,348 |
| Respiratory System | 92,062 | 98,612 | 89,521 | 64,166 | 48,033 | 392,394 |
| Nose, Nasal Cavity and Middle Ear | 679 | 666 | 689 | 553 | 657 | 3,244 |
| Larynx | 2,396 | 1,990 | 1,364 | 846 | 625 | 7,221 |
| Lung and Bronchus | 88,844 | 95,816 | 87,323 | 62,650 | 46,655 | 381,288 |
| Pleura | 32 | 25 | 29 | 29 | 28 | 143 |
| Trachea, Mediastinum and Other Respiratory Organs | 111 | 115 | 116 | 88 | 68 | 498 |
| Bones and Joints | 507 | 470 | 365 | 358 | 370 | 2,070 |
| Soft Tissue including Heart | 2,780 | 2,633 | 2,469 | 2,135 | 2,300 | 12,317 |
| Skin excluding Basal and Squamous | 19,352 | 17,410 | 15,250 | 12,926 | 14,675 | 79,613 |
| Melanoma of the Skin | 17,997 | 15,841 | 13,514 | 11,186 | 12,152 | 70,690 |
| Other Non-Epithelial Skin | 1,355 | 1,569 | 1,736 | 1,740 | 2,523 | 8,923 |
| Breast | 185,007 | 160,606 | 129,852 | 92,002 | 78,859 | 646,326 |
| Female Genital System | 75,850 | 62,582 | 48,714 | 35,142 | 30,729 | 253,017 |
| Cervix Uteri | 6,152 | 4,660 | 3,525 | 2,564 | 2,353 | 19,254 |
| Corpus and Uterus, NOS | 46,348 | 35,936 | 25,563 | 16,639 | 12,589 | 137,075 |
| Corpus Uteri | 45,417 | 35,092 | 24,860 | 16,014 | 11,711 | 133,094 |
| Uterus, NOS | 931 | 844 | 703 | 625 | 878 | 3,981 |
| Ovary | 17,311 | 15,962 | 13,857 | 10,881 | 9,822 | 67,833 |
| Vagina | 1,054 | 995 | 1,020 | 872 | 1,017 | 4,958 |
| Vulva | 2,993 | 3,160 | 3,337 | 3,161 | 4,041 | 16,692 |
| Other Female Genital Organs | 1,992 | 1,869 | 1,412 | 1,025 | 907 | 7,205 |
| Male Genital System | 0 | 0 | 0 | 0 | 0 | 0 |
| Prostate | 0 | 0 | 0 | 0 | 0 | 0 |
| Testis | 0 | 0 | 0 | 0 | 0 | 0 |
| Penis | 0 | 0 | 0 | 0 | 0 | 0 |
| Other Male Genital Organs | 0 | 0 | 0 | 0 | 0 | 0 |
| Urinary System | 30,382 | 31,187 | 30,247 | 25,397 | 25,868 | 143,081 |
| Urinary Bladder | 13,670 | 15,453 | 16,293 | 14,987 | 16,786 | 77,189 |
| Kidney and Renal Pelvis | 15,816 | 14,639 | 12,807 | 9,267 | 7,914 | 60,443 |
| Ureter | 597 | 754 | 845 | 828 | 788 | 3,812 |
| Other Urinary Organs | 299 | 341 | 302 | 315 | 380 | 1,637 |
| Eye and Orbit | 1,020 | 848 | 699 | 585 | 538 | 3,690 |
| Brain and Other Nervous System | 5,960 | 5,730 | 4,945 | 3,575 | 2,908 | 23,118 |
| Brain | 5,670 | 5,481 | 4,721 | 3,435 | 2,766 | 22,073 |
| Cranial Nerves Other Nervous System | 290 | 249 | 224 | 140 | 142 | 1,045 |
| Endocrine System | 12,838 | 9,134 | 6,089 | 3,474 | 2,369 | 33,904 |
| Thyroid | 12,080 | 8,515 | 5,646 | 3,168 | 2,167 | 31,576 |
| Other Endocrine including Thymus | 758 | 619 | 443 | 306 | 202 | 2,328 |
| Lymphoma | 24,246 | 25,214 | 25,049 | 20,917 | 19,835 | 115,261 |
| Hodgkin Lymphoma | 1,172 | 1,131 | 1,059 | 768 | 619 | 4,749 |
| Hodgkin - Nodal | 1,130 | 1,091 | 1,024 | 741 | 593 | 4,579 |
| Hodgkin - Extranodal | 42 | 40 | 35 | 27 | 26 | 170 |
| Non-Hodgkin Lymphoma | 23,074 | 24,083 | 23,990 | 20,149 | 19,216 | 110,512 |
| NHL - Nodal | 15,576 | 16,251 | 16,349 | 13,545 | 12,743 | 74,464 |
| NHL - Extranodal | 7,498 | 7,832 | 7,641 | 6,604 | 6,473 | 36,048 |
| Myeloma | 8,697 | 8,982 | 8,799 | 7,160 | 5,889 | 39,527 |
| Leukemia | 12,989 | 13,801 | 14,245 | 12,869 | 14,587 | 68,491 |
| Lymphocytic Leukemia | 6,478 | 6,753 | 6,874 | 5,968 | 6,774 | 32,847 |
| Acute Lymphocytic Leukemia | 559 | 436 | 420 | 321 | 321 | 2,057 |
| Chronic Lymphocytic Leukemia | 5,682 | 6,050 | 6,167 | 5,373 | 6,096 | 29,368 |
| Other Lymphocytic Leukemia | 237 | 267 | 287 | 274 | 357 | 1,422 |
| Myeloid and Monocytic Leukemia | 5,819 | 6,305 | 6,519 | 5,977 | 6,344 | 30,964 |
| Acute Myeloid Leukemia | 3,876 | 4,321 | 4,349 | 3,936 | 4,090 | 20,572 |
| Acute Monocytic Leukemia | 225 | 216 | 231 | 191 | 201 | 1,064 |
| Chronic Myeloid Leukemia | 1,544 | 1,580 | 1,738 | 1,640 | 1,795 | 8,297 |
| Other Myeloid/Monocytic Leukemia | 174 | 188 | 201 | 210 | 258 | 1,031 |
| Other Leukemia | 692 | 743 | 852 | 924 | 1,469 | 4,680 |
| Other Acute Leukemia | 246 | 295 | 378 | 404 | 702 | 2,025 |
| Aleukemic, Subleukemic and NOS | 446 | 448 | 474 | 520 | 767 | 2,655 |
| Mesothelioma | 569 | 668 | 771 | 622 | 546 | 3,176 |
| Kaposi Sarcoma | 58 | 102 | 139 | 199 | 278 | 776 |
| Miscellaneous | 17,439 | 20,628 | 23,291 | 23,796 | 30,452 | 115,606 |

^a^ Incidence data for 1975-1999 are from the Surveillance, Epidemiology and End Results (SEER) program: Incidence - SEER Research Data, 8 Registries, Nov 2022 Sub (1975-2020) - Linked To County Attributes. Incidence data for 2000-2020 are from the SEER program: Incidence - SEER Research Data, 17 Registries, Nov 2022 Sub (2000-2020) - Linked To County Attributes.
