# Supplementary material for: Trends analysis of cancer incidence, mortality, and survival for the elderly in the United States, 1975–2020
Source: Cancer Med. 2024 Jul 31;13(15):e70062. doi: 10.1002/cam4.70062 (PMC11289898; doi:10.1002/cam4.70062)
Supplement: Supplementary file 1 — Appendix S1. [file CAM4-13-e70062-s001.zip › Supplementary Table 9 All cancer mortality distrib.docx]

**Supplementary Table 9** All cancer mortality distribution by sex and age, United States, 1975-2020^a^

|  | 65-69 years | 70-74 years | 75-79 years | 80-84 years | 85+ years | Total |
| --- | --- | --- | --- | --- | --- | --- |
| Male |  |  |  |  |  |  |
| All Sites | 1,814,294 | 1,979,018 | 1,878,131 | 1,515,650 | 1,427,714 | 8,614,807 |
| Oral Cavity and Pharynx | 41,176 | 35,544 | 27,626 | 19,518 | 17,726 | 141,590 |
| Lip | 352 | 459 | 523 | 431 | 594 | 2,359 |
| Tongue | 9,863 | 8,293 | 6,347 | 4,235 | 3,484 | 32,222 |
| Salivary Gland | 2,701 | 2,843 | 2,908 | 2,680 | 3,437 | 14,569 |
| Floor of Mouth | 1,327 | 1,037 | 658 | 421 | 254 | 3,697 |
| Gum and Other Mouth | 5,412 | 4,844 | 3,988 | 3,103 | 3,347 | 20,694 |
| Nasopharynx | 2,854 | 2,326 | 1,676 | 1,057 | 755 | 8,668 |
| Tonsil | 3,906 | 2,990 | 1,960 | 1,220 | 831 | 10,907 |
| Oropharynx | 3,493 | 2,848 | 2,102 | 1,426 | 1,115 | 10,984 |
| Hypopharynx | 2,868 | 2,394 | 1,730 | 1,045 | 641 | 8,678 |
| Other Oral Cavity and Pharynx | 8,400 | 7,510 | 5,734 | 3,900 | 3,268 | 28,812 |
| Digestive System | 478,958 | 502,179 | 465,768 | 369,764 | 336,371 | 2,153,040 |
| Esophagus | 66,929 | 63,180 | 51,595 | 36,221 | 27,704 | 245,629 |
| Stomach | 48,557 | 53,563 | 51,767 | 41,563 | 38,662 | 234,112 |
| Small Intestine | 3,636 | 3,958 | 3,698 | 2,969 | 2,582 | 16,843 |
| Colon and Rectum | 174,849 | 193,872 | 189,218 | 159,356 | 159,875 | 877,170 |
| Colon excluding Rectum | 142,544 | 160,850 | 159,165 | 134,878 | 135,624 | 733,061 |
| Rectum and Rectosigmoid Junction | 32,305 | 33,022 | 30,053 | 24,478 | 24,251 | 144,109 |
| Anus, Anal Canal and Anorectum | 1,229 | 1,127 | 900 | 776 | 755 | 4,787 |
| Liver and Intrahepatic Bile Duct | 62,939 | 57,865 | 49,936 | 36,878 | 28,512 | 236,130 |
| Liver | 52,860 | 47,283 | 40,127 | 29,222 | 21,852 | 191,344 |
| Intrahepatic Bile Duct | 10,079 | 10,582 | 9,809 | 7,656 | 6,660 | 44,786 |
| Gallbladder | 3,980 | 5,029 | 5,074 | 3,978 | 3,649 | 21,710 |
| Other Biliary | 5,202 | 6,306 | 6,401 | 5,359 | 5,348 | 28,616 |
| Pancreas | 107,228 | 112,446 | 102,687 | 78,711 | 65,308 | 466,380 |
| Retroperitoneum | 987 | 1,078 | 927 | 745 | 580 | 4,317 |
| Peritoneum, Omentum and Mesentery | 888 | 874 | 700 | 491 | 368 | 3,321 |
| Other Digestive Organs | 2,534 | 2,881 | 2,865 | 2,717 | 3,028 | 14,025 |
| Respiratory System | 687,599 | 713,931 | 610,228 | 415,883 | 282,053 | 2,709,694 |
| Nose, Nasal Cavity and Middle Ear | 1,823 | 1,757 | 1,604 | 1,212 | 1,139 | 7,535 |
| Larynx | 23,194 | 20,920 | 16,885 | 11,943 | 9,469 | 82,411 |
| Lung and Bronchus | 659,432 | 687,890 | 588,596 | 400,461 | 269,768 | 2,606,147 |
| Pleura | 1,823 | 2,154 | 2,097 | 1,555 | 1,124 | 8,753 |
| Trachea, Mediastinum and Other Respiratory Organs | 1,327 | 1,210 | 1,046 | 712 | 553 | 4,848 |
| Bones and Joints | 2,966 | 3,141 | 2,959 | 2,534 | 2,540 | 14,140 |
| Soft Tissue including Heart | 9,036 | 9,492 | 8,928 | 7,228 | 6,665 | 41,349 |
| Skin | 33,239 | 34,317 | 33,647 | 29,690 | 35,369 | 166,262 |
| Melanoma of the Skin | 23,934 | 24,514 | 23,444 | 19,474 | 19,367 | 110,733 |
| Non-Melanoma Skin | 9,305 | 9,803 | 10,203 | 10,216 | 16,002 | 55,529 |
| Breast | 2,277 | 2,390 | 2,299 | 1,962 | 2,050 | 10,978 |
| Female Genital System | 0 | 0 | 0 | 0 | 0 | 0 |
| Cervix Uteri | 0 | 0 | 0 | 0 | 0 | 0 |
| Corpus and Uterus, NOS | 0 | 0 | 0 | 0 | 0 | 0 |
| Corpus Uteri | 0 | 0 | 0 | 0 | 0 | 0 |
| Uterus, NOS | 0 | 0 | 0 | 0 | 0 | 0 |
| Ovary | 0 | 0 | 0 | 0 | 0 | 0 |
| Vagina | 0 | 0 | 0 | 0 | 0 | 0 |
| Vulva | 0 | 0 | 0 | 0 | 0 | 0 |
| Other Female Genital Organs | 0 | 0 | 0 | 0 | 0 | 0 |
| Male Genital System | 137,359 | 203,223 | 253,777 | 265,008 | 343,410 | 1,202,777 |
| Prostate | 135,148 | 200,997 | 251,700 | 263,092 | 341,106 | 1,192,043 |
| Testis | 620 | 522 | 404 | 388 | 415 | 2,349 |
| Penis | 1,337 | 1,433 | 1,354 | 1,263 | 1,523 | 6,910 |
| Other Male Genital Organs | 254 | 271 | 319 | 265 | 366 | 1,475 |
| Urinary System | 93,108 | 108,378 | 113,142 | 103,700 | 118,589 | 536,917 |
| Urinary Bladder | 44,737 | 58,867 | 67,995 | 68,570 | 86,179 | 326,348 |
| Kidney and Renal Pelvis | 46,422 | 47,023 | 42,297 | 32,551 | 29,610 | 197,903 |
| Ureter | 1,094 | 1,438 | 1,603 | 1,376 | 1,416 | 6,927 |
| Other Urinary Organs | 855 | 1,050 | 1,247 | 1,203 | 1,384 | 5,739 |
| Eye and Orbit | 904 | 878 | 769 | 697 | 655 | 3,903 |
| Brain and Other Nervous System | 42,249 | 38,561 | 30,364 | 19,324 | 12,719 | 143,217 |
| Endocrine System | 5,558 | 5,551 | 5,060 | 3,925 | 3,377 | 23,471 |
| Thyroid | 3,603 | 3,719 | 3,589 | 2,919 | 2,623 | 16,453 |
| Other Endocrine including Thymus | 1,955 | 1,832 | 1,471 | 1,006 | 754 | 7,018 |
| Lymphoma | 59,554 | 69,601 | 71,290 | 61,524 | 56,722 | 318,691 |
| Hodgkin Lymphoma | 3,575 | 3,767 | 3,241 | 2,490 | 1,962 | 15,035 |
| Non-Hodgkin Lymphoma | 55,979 | 65,834 | 68,049 | 59,034 | 54,760 | 303,656 |
| Myeloma | 33,138 | 39,041 | 39,155 | 32,705 | 27,974 | 172,013 |
| Leukemia | 59,586 | 74,196 | 79,205 | 70,083 | 69,104 | 352,174 |
| Lymphocytic Leukemia | 16,330 | 20,387 | 22,515 | 21,391 | 25,869 | 106,492 |
| Acute Lymphocytic Leukemia | 2,359 | 2,447 | 2,324 | 1,880 | 1,744 | 10,754 |
| Chronic Lymphocytic Leukemia | 12,590 | 16,102 | 18,129 | 17,544 | 21,664 | 86,029 |
| Other Lymphocytic Leukemia | 1,381 | 1,838 | 2,062 | 1,967 | 2,461 | 9,709 |
| Myeloid and Monocytic Leukemia | 30,502 | 36,687 | 37,695 | 31,047 | 25,270 | 161,201 |
| Acute Myeloid Leukemia | 23,463 | 28,222 | 28,513 | 22,824 | 17,583 | 120,605 |
| Acute Monocytic Leukemia | 459 | 609 | 692 | 587 | 486 | 2,833 |
| Chronic Myeloid Leukemia | 4,760 | 5,395 | 5,520 | 4,785 | 4,539 | 24,999 |
| Other Myeloid/Monocytic Leukemia | 1,820 | 2,461 | 2,970 | 2,851 | 2,662 | 12,764 |
| Other Leukemia | 12,754 | 17,122 | 18,995 | 17,645 | 17,965 | 84,481 |
| Other Acute Leukemia | 6,265 | 8,071 | 8,779 | 7,526 | 6,599 | 37,240 |
| Aleukemic, Subleukemic and NOS | 6,489 | 9,051 | 10,216 | 10,119 | 11,366 | 47,241 |
| Miscellaneous Malignant Cancer | 127,587 | 138,595 | 133,914 | 112,105 | 112,390 | 624,591 |
| Female |  |  |  |  |  |  |
| All Sites | 1,404,978 | 1,570,458 | 1,589,285 | 1,426,630 | 1,729,675 | 7,721,026 |
| Oral Cavity and Pharynx | 15,588 | 16,003 | 15,339 | 14,075 | 20,232 | 81,237 |
| Lip | 70 | 87 | 124 | 182 | 417 | 880 |
| Tongue | 3,864 | 3,916 | 3,742 | 3,545 | 4,731 | 19,798 |
| Salivary Gland | 1,300 | 1,426 | 1,555 | 1,574 | 2,808 | 8,663 |
| Floor of Mouth | 526 | 514 | 402 | 332 | 389 | 2,163 |
| Gum and Other Mouth | 2,698 | 3,168 | 3,528 | 3,865 | 6,842 | 20,101 |
| Nasopharynx | 1,267 | 1,177 | 1,006 | 821 | 877 | 5,148 |
| Tonsil | 1,259 | 1,042 | 909 | 641 | 581 | 4,432 |
| Oropharynx | 1,130 | 1,167 | 1,126 | 928 | 1,254 | 5,605 |
| Hypopharynx | 741 | 693 | 535 | 347 | 317 | 2,633 |
| Other Oral Cavity and Pharynx | 2,733 | 2,813 | 2,412 | 1,840 | 2,016 | 11,814 |
| Digestive System | 312,362 | 376,171 | 415,487 | 406,900 | 548,429 | 2,059,349 |
| Esophagus | 16,268 | 17,839 | 17,564 | 15,787 | 19,436 | 86,894 |
| Stomach | 24,913 | 31,071 | 35,818 | 36,341 | 49,794 | 177,937 |
| Small Intestine | 2,870 | 3,166 | 3,324 | 3,106 | 3,733 | 16,199 |
| Colon and Rectum | 133,532 | 162,822 | 185,372 | 190,664 | 284,128 | 956,518 |
| Colon excluding Rectum | 113,612 | 140,309 | 160,540 | 166,320 | 246,289 | 827,070 |
| Rectum and Rectosigmoid Junction | 19,920 | 22,513 | 24,832 | 24,344 | 37,839 | 129,448 |
| Anus, Anal Canal and Anorectum | 1,933 | 1,893 | 1,715 | 1,590 | 2,164 | 9,295 |
| Liver and Intrahepatic Bile Duct | 27,891 | 32,021 | 34,430 | 31,386 | 36,011 | 161,739 |
| Liver | 19,197 | 22,297 | 24,498 | 22,442 | 25,927 | 114,361 |
| Intrahepatic Bile Duct | 8,694 | 9,724 | 9,932 | 8,944 | 10,084 | 47,378 |
| Gallbladder | 8,738 | 10,681 | 11,716 | 10,697 | 11,823 | 53,655 |
| Other Biliary | 4,599 | 5,897 | 6,731 | 6,825 | 9,189 | 33,241 |
| Pancreas | 86,314 | 104,597 | 112,098 | 104,034 | 123,287 | 530,330 |
| Retroperitoneum | 819 | 905 | 924 | 798 | 859 | 4,305 |
| Peritoneum, Omentum and Mesentery | 2,803 | 3,236 | 3,151 | 2,689 | 2,407 | 14,286 |
| Other Digestive Organs | 1,682 | 2,043 | 2,644 | 2,983 | 5,598 | 14,950 |
| Respiratory System | 389,068 | 426,791 | 394,549 | 299,650 | 260,619 | 1,770,677 |
| Nose, Nasal Cavity and Middle Ear | 998 | 1,132 | 1,206 | 1,184 | 1,728 | 6,248 |
| Larynx | 5,525 | 5,259 | 4,383 | 2,928 | 2,596 | 20,691 |
| Lung and Bronchus | 381,366 | 419,032 | 387,623 | 294,363 | 255,017 | 1,737,401 |
| Pleura | 487 | 549 | 609 | 533 | 554 | 2,732 |
| Trachea, Mediastinum and Other Respiratory Organs | 692 | 819 | 728 | 642 | 724 | 3,605 |
| Bones and Joints | 2,076 | 2,441 | 2,681 | 2,628 | 3,674 | 13,500 |
| Soft Tissue including Heart | 8,683 | 9,068 | 8,889 | 7,615 | 8,656 | 42,911 |
| Skin | 14,918 | 16,319 | 17,214 | 17,218 | 28,973 | 94,642 |
| Melanoma of the Skin | 12,008 | 12,874 | 13,087 | 12,380 | 16,539 | 66,888 |
| Non-Melanoma Skin | 2,910 | 3,445 | 4,127 | 4,838 | 12,434 | 27,754 |
| Breast | 219,351 | 216,996 | 203,862 | 178,218 | 236,849 | 1,055,276 |
| Female Genital System | 161,080 | 164,662 | 153,905 | 130,299 | 139,584 | 749,530 |
| Cervix Uteri | 19,891 | 17,505 | 15,034 | 12,375 | 13,213 | 78,018 |
| Corpus and Uterus, NOS | 51,017 | 52,700 | 47,482 | 39,712 | 44,576 | 235,487 |
| Corpus Uteri | 27,157 | 27,613 | 24,073 | 18,859 | 19,362 | 117,064 |
| Uterus, NOS | 23,860 | 25,087 | 23,409 | 20,853 | 25,214 | 118,423 |
| Ovary | 82,231 | 85,296 | 81,180 | 67,633 | 65,270 | 381,610 |
| Vagina | 1,880 | 2,186 | 2,464 | 2,624 | 4,192 | 13,346 |
| Vulva | 3,337 | 4,143 | 5,208 | 6,050 | 10,224 | 28,962 |
| Other Female Genital Organs | 2,724 | 2,832 | 2,537 | 1,905 | 2,109 | 12,107 |
| Male Genital System | 0 | 0 | 0 | 0 | 0 | 0 |
| Prostate | 0 | 0 | 0 | 0 | 0 | 0 |
| Testis | 0 | 0 | 0 | 0 | 0 | 0 |
| Penis | 0 | 0 | 0 | 0 | 0 | 0 |
| Other Male Genital Organs | 0 | 0 | 0 | 0 | 0 | 0 |
| Urinary System | 39,851 | 50,287 | 57,162 | 59,776 | 88,062 | 295,138 |
| Urinary Bladder | 15,125 | 21,609 | 27,440 | 31,852 | 53,641 | 149,667 |
| Kidney and Renal Pelvis | 23,426 | 26,955 | 27,678 | 25,666 | 31,329 | 135,054 |
| Ureter | 649 | 979 | 1,159 | 1,313 | 1,712 | 5,812 |
| Other Urinary Organs | 651 | 744 | 885 | 945 | 1,380 | 4,605 |
| Eye and Orbit | 702 | 814 | 811 | 729 | 1,117 | 4,173 |
| Brain and Other Nervous System | 33,346 | 33,202 | 28,828 | 21,252 | 17,905 | 134,533 |
| Endocrine System | 5,800 | 6,782 | 7,346 | 6,812 | 8,452 | 35,192 |
| Thyroid | 4,045 | 4,993 | 5,758 | 5,638 | 7,293 | 27,727 |
| Other Endocrine including Thymus | 1,755 | 1,789 | 1,588 | 1,174 | 1,159 | 7,465 |
| Lymphoma | 45,522 | 58,719 | 68,627 | 68,339 | 80,516 | 321,723 |
| Hodgkin Lymphoma | 2,600 | 2,937 | 3,296 | 2,874 | 2,661 | 14,368 |
| Non-Hodgkin Lymphoma | 42,922 | 55,782 | 65,331 | 65,465 | 77,855 | 307,355 |
| Myeloma | 26,390 | 33,143 | 36,518 | 34,099 | 35,643 | 165,793 |
| Leukemia | 37,145 | 48,788 | 57,535 | 59,354 | 83,656 | 286,478 |
| Lymphocytic Leukemia | 8,320 | 11,663 | 15,055 | 17,183 | 31,160 | 83,381 |
| Acute Lymphocytic Leukemia | 1,946 | 2,075 | 2,205 | 1,922 | 2,323 | 10,471 |
| Chronic Lymphocytic Leukemia | 5,707 | 8,574 | 11,552 | 13,705 | 26,220 | 65,758 |
| Other Lymphocytic Leukemia | 667 | 1,014 | 1,298 | 1,556 | 2,617 | 7,152 |
| Myeloid and Monocytic Leukemia | 20,394 | 25,583 | 28,055 | 26,409 | 28,925 | 129,366 |
| Acute Myeloid Leukemia | 15,971 | 19,917 | 21,415 | 19,377 | 19,621 | 96,301 |
| Acute Monocytic Leukemia | 355 | 415 | 491 | 516 | 573 | 2,350 |
| Chronic Myeloid Leukemia | 3,086 | 3,769 | 4,263 | 4,481 | 5,994 | 21,593 |
| Other Myeloid/Monocytic Leukemia | 982 | 1,482 | 1,886 | 2,035 | 2,737 | 9,122 |
| Other Leukemia | 8,431 | 11,542 | 14,425 | 15,762 | 23,571 | 73,731 |
| Other Acute Leukemia | 4,394 | 5,808 | 6,846 | 6,909 | 8,913 | 32,870 |
| Aleukemic, Subleukemic and NOS | 4,037 | 5,734 | 7,579 | 8,853 | 14,658 | 40,861 |
| Miscellaneous Malignant Cancer | 93,096 | 110,272 | 120,532 | 119,666 | 167,308 | 610,874 |

^a^ Mortality data are from the Surveillance, Epidemiology, and End Results (SEER) database: Mortality - All COD, Aggregated Total U.S. (1969-2020) <Katrina/Rita Population Adjustment>, National Cancer Institute, DCCPS, Surveillance Research Program, released June 2022.
