# Supplementary figures and images for: Trends analysis of cancer incidence, mortality, and survival for the elderly in the United States, 1975–2020
Source: Cancer Med. 2024 Jul 31;13(15):e70062. doi: 10.1002/cam4.70062 (PMC11289898; doi:10.1002/cam4.70062)

**Supplementary Figure 6** Relative 1-, 3- and 5-year survival trends for the rest cancers, United States, 1975-2019.


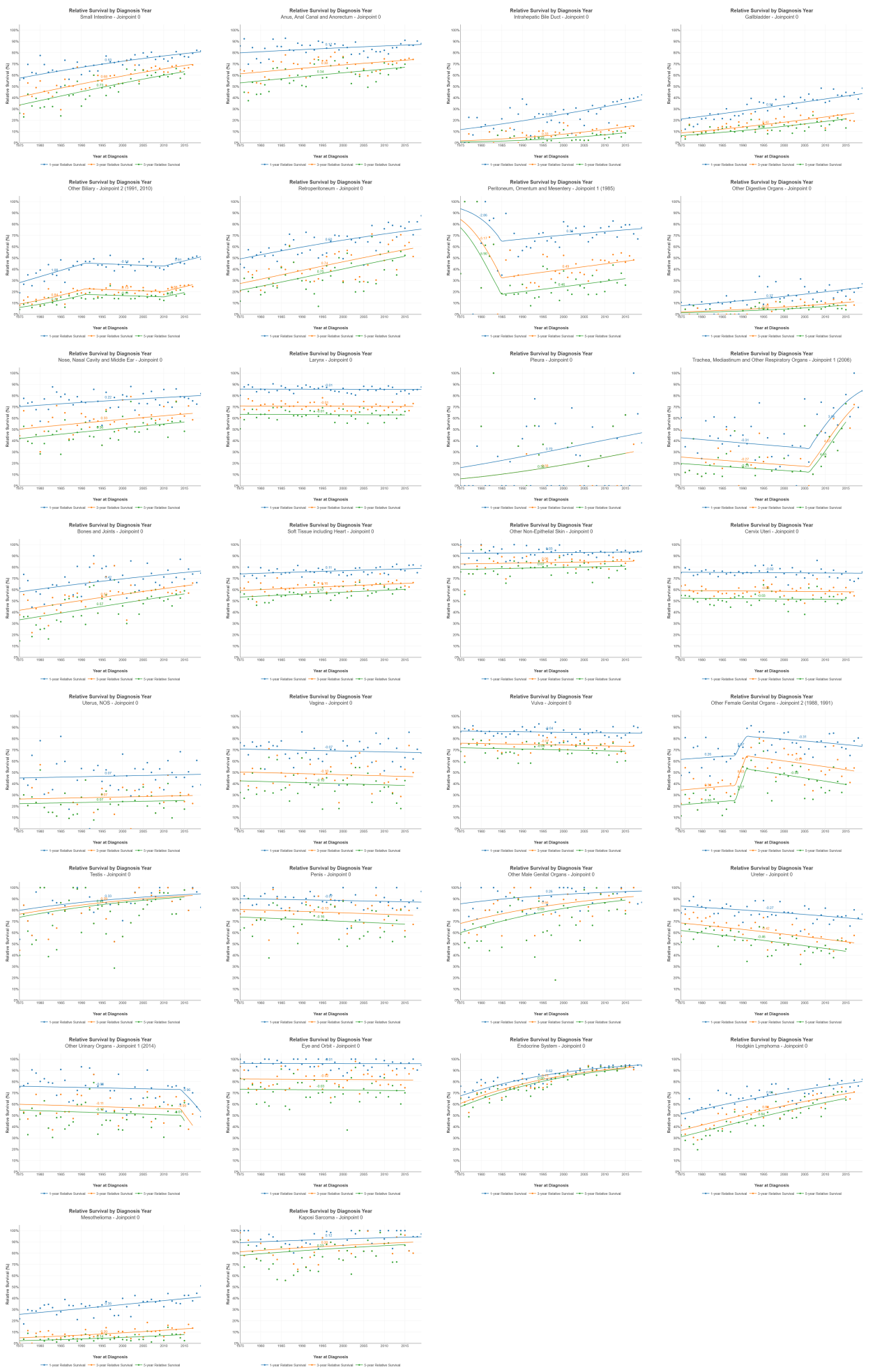

Supplement: Supplementary file 1 — Appendix S1. [file CAM4-13-e70062-s001.zip › Supplementary Figure 6 Relative 1.docx]
